# Temporal Stretch‐Induced Nuclear Mechanosensing Coordinates Early Chromatin Accessibility and Genome Protection

**DOI:** 10.1002/advs.202510554

**Published:** 2025-10-07

**Authors:** Hye‐Won Shim, Ji‐Young Yoon, Hwalim Lee, Shanika Karunasagara, Cheng Ji Li, Yongdae Shin, Sukbum Hong, Dong‐Joon Lee, Jung‐Hwan Lee, Kam W. Leong, Hae‐Won Kim

**Affiliations:** ^1^ Institute of Tissue Regeneration Engineering (ITREN) Dankook University Cheonan 31116 South Korea; ^2^ Department of Nanobiomedical Science & BK21 NBM Global Research Center for Regenerative Medicine Dankook University Cheonan 31116 Republic of Korea; ^3^ Mechanobiology Dental Medicine Research Center Dankook University Cheonan 31116 Republic of Korea; ^4^ Institute for Stem Cell and Matter Cell & Matter Corporation Cheonan 31116 Republic of Korea; ^5^ Cell and Matter Institute Dankook University Cheonan 31116 Republic of Korea; ^6^ Department of Biomaterials Science College of Dentistry Dankook University Cheonan 31116 Republic of Korea; ^7^ Department of Mechanical Engineering Seoul National University Seoul 08826 Republic of Korea; ^8^ Interdisciplinary Program in Bioengineering Seoul National University Seoul 08826 Republic of Korea; ^9^ Department of Oral Histology College of Dentistry Dankook University Cheonan 31116 Republic of Korea; ^10^ Department of Biomedical Engineering Columbia University New York NY 10027 USA

**Keywords:** chromatin modifications, fibroblasts, mechanosensitive molecules, nuclear mechanosensing, nuclear protection, temporal stretch

## Abstract

Cells respond to mechanical stimuli by transmitting forces to the nucleus, activating mechanosensitive molecules that alter chromatin organization and gene expression. While force‐induced changes in cell fate are recognized, the spatiotemporal dynamics of nuclear mechanosensing remain unclear. Here, nuclear responses are investigated to temporal cyclic stretching in human dermal fibroblasts, uncovering a cascade of mechanosensitive events linking cytoskeletal remodeling, chromatin accessibility, and gene expression. Brief cyclic stretch induces rapid chromatin decondensation and nuclear softening, marked by reduced H3K9me3 levels. The stretch reinforces perinuclear actin assembly from globular actins, activated by Ca^2+^ release from the nucleus/endoplasmic reticulum. Notably, perinuclear actin remodeling correlated with decreased H3K9me3 coordinates through emerin translocation at the nuclear envelope. Genome‐wide profiling reveals increased accessibility of loci associated with mechanotransduction and DNA damage repair. Failure to coordinate these events results in DNA damage due to impaired chromatin decondensation, demonstrating a biophysical mechanism protecting genomic integrity. These nuclear events are further evidenced in vivo using a skin tissue model, where spatial transcriptomics confirm mechanosensitive chromatin reorganization through actin‐dependent pathways in dermal fibroblasts. This study illuminates mechanisms by which temporally regulated mechanical forces elicit nuclear mechanosensing responses, linking perinuclear mechanosensitive molecules to epigenetic remodeling and downstream regulation of cell fate.

## Graphical abstract

Cyclic stretching of fibroblasts triggers coordinated nuclear mechanosensing events, including calcium ion release, perinuclear actin assembly, emerin translocation, and H3K9me3 loss, increasing chromatin accessibility for specific genes related to mechanotransduction and repair.

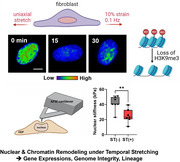

## 1 Introduction

Mechanical forces profoundly influence various pathophysiological processes at the biomolecular, cellular, and tissue levels.^[^ ^1^, ^2^, ^3^, ^4^ ^]^ Exploring biological phenomena through the lens of cell–force interactions helps decipher how cells sustain damage, restore function, and maintain tissue homeostasis.^[^ ^5^, ^6^, ^7^, ^8^ ^]^ External forces transmit through the cytoskeleton to the nucleus, activating mechanosensitive molecules and altering nuclear mechanics.^[^ ^9^, ^10^, ^11^, ^12^ ^]^ These mechanotransmission and mechanosensing processes regulate diverse cellular events, such as proliferation, migration, metabolic activity, apoptosis, and differentiation.^[^ ^13^, ^14^, ^15^, ^16^, ^17^ ^]^ In particular, mechanical forces mediate epigenetic alterations, notably histone modifications, which affect the accessibility of genomic loci and gene expression.^[^ ^16^, ^18^, ^19^, ^20^, ^21^, ^22^ ^]^

Skin tissue, composed primarily of epithelial cells and fibroblasts, functions as a load‐bearing structure that undergoes substantial force‐driven, anisotropic deformation.^[^ ^23^ ^]^ Stretching, in particular, is a key biophysical force in clinical settings that causes hypertrophic scar formation and regulates regeneration processes.^[^ ^24^, ^25^, ^26^ ^]^ Chronic mechanical stress can compromise tissue integrity,^[^ ^27^ ^]^ yet epithelial sheets can endure extreme stretching without damage due to heterochromatin‐driven nuclear softening and remodeling of the linker of nucleoskeleton and cytoskeleton (LINC) complex.^[^ ^16^, ^28^ ^]^ Despite these findings, stretch‐induced mechanosensing at the nucleus and the biological mechanisms related to epigenetic alterations and gene expression remain largely unexplored in skin fibroblasts.

In this study, we investigate the impact of stretching as a biophysical cue in skin fibroblasts, focusing on the role of nuclear‐associated mechanosensitive molecules and epigenetic changes in compensating for the force. We demonstrate that transient stretch stimuli, on the order of tens of minutes, induce rapid chromatin modification and alteration in nuclear mechanics. Stretching also induces reorganization of the perinuclear actin cytoskeleton, which closely correlates with changes in chromatin state. These events are orchestrated by immediate calcium release from the endoplasmic reticulum and dynamic modulation of emerin at the nuclear envelope. Through genome‐wide profiling of accessible chromatin regions, we identify increased accessibility of genomic loci associated with mechanotransduction and DNA damage repair processes. We further validate these mechanobiological cellular events in an in vivo skin model.

Our study elucidates the mechanisms by which mechanically stressed cells sense and respond to forces within and near the nucleus, linking mechanosensitive nuclear‐associated molecules, epigenetic changes, and cellular protection processes.

## 2 Results and Discussion

### 2.1 Temporal Cyclic Stretch Rapidly Induces Epigenetic Remodeling toward Euchromatin

**Figure 1:**
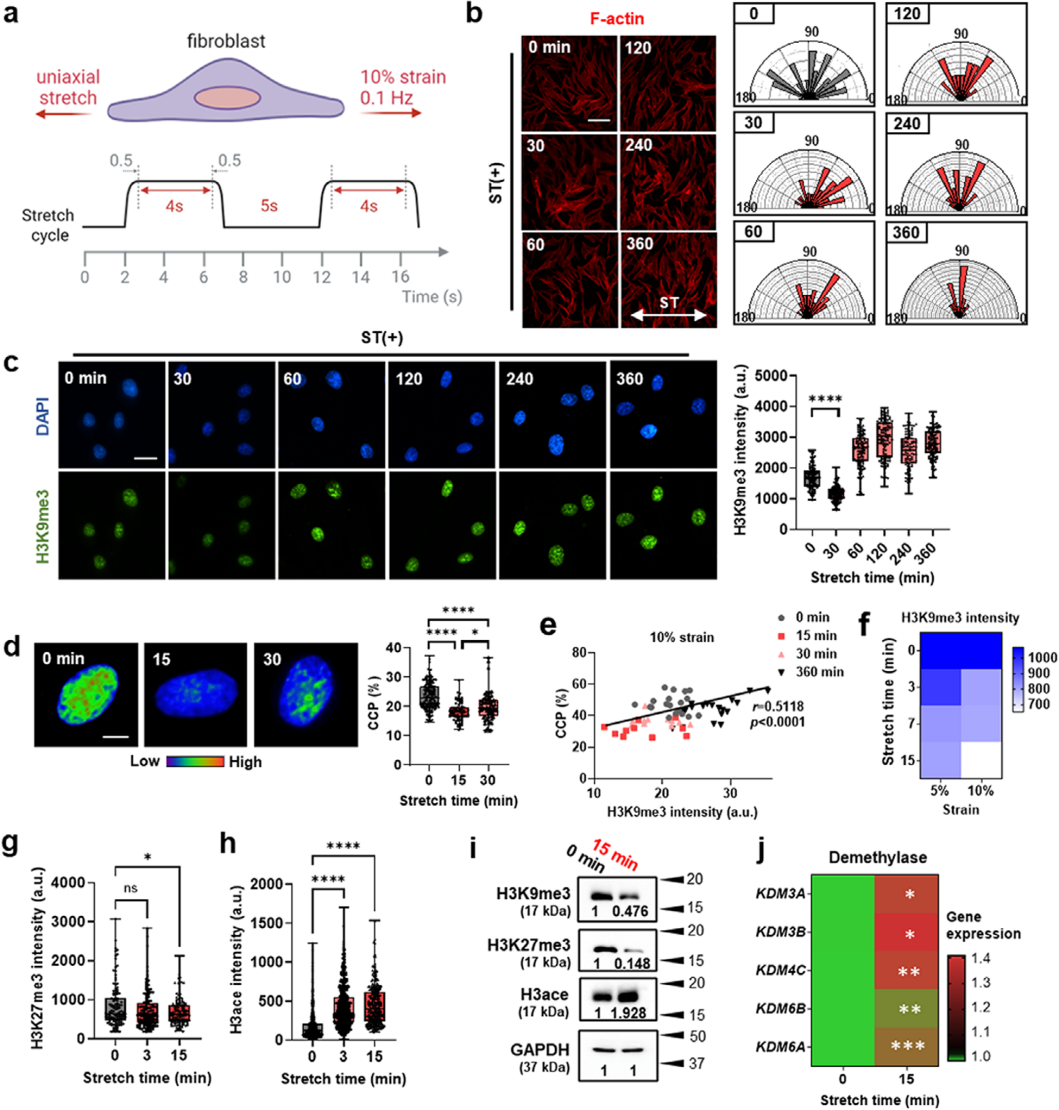
Cyclic stretch induces rapid and transient epigenetic remodeling toward euchromatin in dermal fibroblasts. a) A schematic depicts the application of uniaxial cyclic stretch conditions (denoted as “ST”), characterized by a 0.1 Hz frequency with a 4 s stretch phase, a 5 s rest phase, and a 0.5 s offset between phases at 10% strain. b) Phalloidin‐stained cell images after stretching (10% strain, 0.1 Hz, 0–360 mins) reveal cellular alignment perpendicular to the direction of stretch, indicative of mechanosensitivity to the applied force. Scale bar: 100 µm. c) Immunofluorescence staining images and semi‐quantitative analysis of H3K9me3 demonstrate a rapid reduction at 30 min post‐stretch (*n *= 110). Scale bar: 20 µm. d) 4',6‐diamidino‐2‐phenylindole (DAPI)‐stained nuclei rendered in rainbow channel and analyzed for chromatin condensation parameter (CCP) show pronounced chromatin decondensation, most evident at 15 min post‐stretch (*n *> 74). Scale bar: 5 µm. e) Positive correlation between H3K9me3 intensity and DAPI CCP (%) (Pearson's correlation value *r *= 0.5118, *p *< 0.0001), supporting the link between H3K9me3 loss and chromatin decondensation. f) Heatmap depicts H3K9me3 expression intensity across various stretch conditions, highlighting maximal reduction at 10% strain and 15 min duration (*n *> 100). g,h) Semi‐quantitative analysis of immunofluorescence staining images reveals changes in H3K27me3 and H3ace levels at 15 min post‐stretch (*n *= 125 for H3K27me3, *n *> 279 for H3ace). i) Western blot and semi‐quantitative analysis indicate a stretch‐induced increase in H3ace and decreases in H3K9me3 and H3K27me3, indicative of global epigenetic transition toward euchromatin. j) Heatmap representation of gene expressions in histone lysine demethylases by quantitative polymerase chain reaction (qPCR), comparing 0 min versus 15 min of stretch (*n *= 3), suggesting potential involvement of epigenetic modifiers at the gene level in response to cyclic stretch. Statistical significance is denoted as *p < 0.5, **p < 0.01, ***p < 0.001, and ****p < 0.0001, determined by one‐way ANOVA with Dunnett's post hoc test. The data represent three independent biological experiments. All experiments were analyzed immediately after specific stretch timepoints.

To investigate the impact of temporal cyclic stretch on human dermal fibroblasts (HDFs), we cultured cells on thin, stretchable polydimethylsiloxane (PDMS) with collagen type I coating following synchronization of cell cycles under serum‐free medium on conventional plastic culture plates. Employing previously optimized conditions that resulted in epigenetic changes,^[^ ^15^ ^]^ we applied cyclic uniaxial stretch (10%, 0.1 Hz), consisting of a 4 s stretch phase, a 5 s rest phase, and a 0.5 s offset between phases, for up to 6 h (**Figure** **1**a; Figure S1, Supporting Information). Cells exhibited a perpendicular alignment against the stretch direction over time without any evidence of cell death or detachment, as previously reported (Figure 1b).^[^ ^29^, ^30^ ^]^ Stretching also induced dynamic epigenetic remodeling. We first analyzed a well‐known mechanosensitive epigenetic marker histone 3 lysine 9 trimethylation (H3K9me3), associated with gene silencing and heterochromatin formation.^[^ ^28^, ^31^, ^32^, ^33^, ^34^ ^]^ H3K9me3 level displayed a rapid decrease at 30 min of cyclic stretch, followed by an increase over the subsequent 6 h (Figure 1c). Chromatin intensity analysis revealed concurrent chromatin decondensation, most prominently at 15 min (Figure 1d; Figure S2, Supporting Information), and these changes were strongly correlated with H3K9me3 reduction (Figure 1e). A systematic analysis across varying durations (0–15 min) and strain magnitudes (0–10%) identified 10% strain for 15 min as the most effective condition for H3K9me3 reduction and chromatin decondensation (Figure 1f; Figure S3a, Supporting Information). To examine whether this epigenetic response is a general phenomenon across different stretch conditions, we further tested multiple frequencies (0.05, 0.1, 0.2, and 0.5 Hz) with fixed strain (10%) and multiple strain levels (5%, 10%, and 20%) with fixed frequency (0.1 Hz). These comprehensive screening experiments confirmed that while 0.1 Hz frequency and 10% strain provided optimal epigenetic responses, other conditions also showed tendencies toward chromatin decondensation and H3K9me3 reduction, albeit to a lesser extent, demonstrating the general applicability of stretch‐induced epigenetic remodeling. (Figure S3b,c, Supporting Information)

In addition to H3K9me3, we examined other histone modifications by immunofluorescence imaging and Western blotting. A marked decrease in heterochromatin‐associated H3K27me3 and an increase in euchromatin‐associated H3 acetylation (H3ace) were observed, indicating a global transition toward euchromatin upon stretch^[^ ^16^, ^18^ ^]^ (Figure 1g–i; Figure S3d, Supporting Information). Furthermore, we analyzed the involvement of histone methylation writers (histone methyltransferases; HMTs) and erasers (histone demethylases; HDMs) targeting H3K9 and H3K27. The mRNA levels of *HDMs* (*KDM3A*, *KDM3B*, *KDM4C*, *KDM6B*, and *KDM6A*) were profoundly upregulated upon stretch (Figure 1j; Figure S4, Supporting Information), whereas those of HMTs did not significantly alter (Figure S5, Supporting Information). This rapid transcriptional response is consistent with previous studies demonstrating that stretching stimuli can induce gene expression changes within minutes to tens of minutes, including chromatin modifiers and mechanosensitive genes.^[^ ^28^, ^35^, ^36^ ^]^ Additionally, we examined nuclear morphology to determine whether mechanical stretch caused physical changes in nuclear architecture that could influence chromatin structure.^[^ ^37^ ^]^ Analysis of 2D‐projection and 3D‐constructed images of the nucleus revealed no significant alterations in nuclear morphology (Figure S6, Supporting Information). While 3D volume reconstruction revealed no significant differences in nuclear morphology, we acknowledge the inherent limitations of confocal‐based volume measurements, including potential artifacts from refractive index mismatches and limited z‐resolution. Intriguingly, the stretch‐induced decrease in H3K9me3 was transient, with levels returning to baseline within hours of rest (Figure S7, Supporting Information), indicating the reversible nature of this epigenetic response. This rapid chromatin remodeling likely reflects our higher strain magnitude (10%) compared to studies using lower strains, where actin reorganization occurred over longer timescales with hours of resting period.^[^ ^38^ ^]^ The transient nature of these changes indicates that sustained mechanical input is required to maintain chromatin accessibility, representing an adaptive mechanism for rapid mechanosensing.

Taken together, these findings suggest that temporal cyclic stretch triggers a rapid, transient shift in chromatin state toward euchromatin, marked by reductions in H3K9me3 and H3K27me3 and increased H3 acetylation. These changes likely involve the activation of specific epigenetic modifiers in response to mechanical stimuli.

### 2.2 Cyclic Stretch Leads to Loss of Perinuclear Heterochromatin and Nuclear Softening

**Figure 2:**
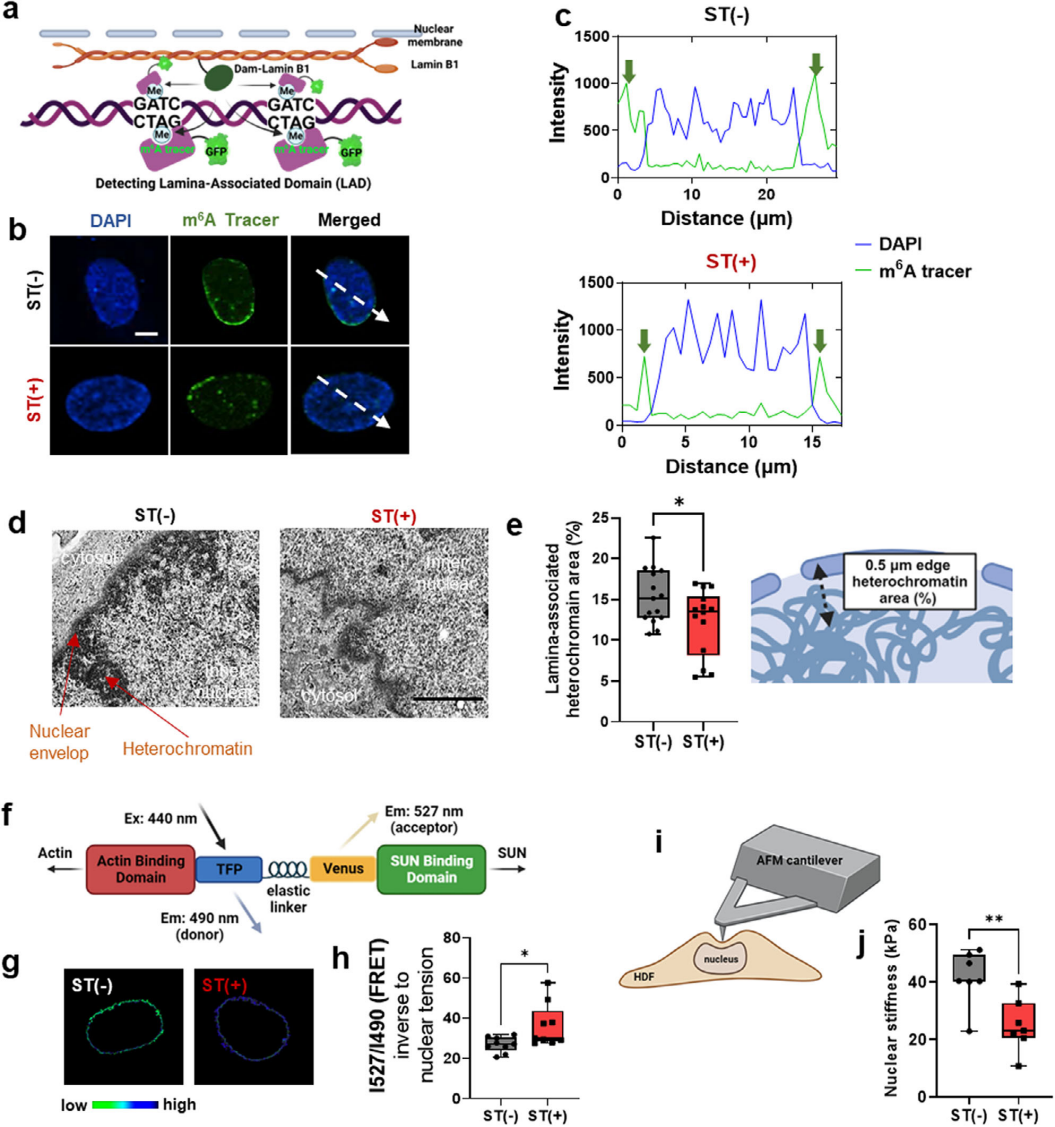
Cyclic stretch disrupts perinuclear heterochromatin and reduces nuclear stiffness. a) Schematic illustrating the DNA adenine methyltransferase identification (DamID) technique for visualizing lamina‐associated domains (LADs). The Dam‐Lamin B1 fusion protein introduces N^6^‐methyladenine (m^6^A) marks at GATC motifs in genomic regions near the nuclear lamina. These methylation marks are specifically recognized by the m^6^A‐Tracer and could be detected by a GFP‐tagged DNA‐binding protein to visualize lamina‐associated domains. b) Representative confocal images of cells co‐transfected with m^6^A‐Tracer and Dam‐LMNB1, showing a reduction in LAD‐associated heterochromatin upon cyclic stretch. (Scale bar: 5 µm) c) Fluorescence intensity profiles of the m^6^A‐Tracer and DAPI along the nuclear axis. d) Representative transmission electron microscopy (TEM) images of nuclear periphery with (ST(+)) and without stretch (ST(−)). e) Quantification of heterochromatin area at the nuclear periphery based on TEM images, corresponding to LAD‐associated regions, with a schematic illustrating the measurement area. Heterochromatin loss was consistently observed across all analyzed cells. Quantification was performed within a 0.5 µm band from the nuclear edge, covering the entire nuclear periphery of each cell. Stretch‐induced loss of peripheral heterochromatin and increased nuclear wrinkling. (Scale bar: 2 µm; *n *= 15). f) Schematic depiction of the fluorescence resonance energy transfer (FRET)‐based Nesprin‐2 tension sensor (TS), a critical element of the LINC complex. g,h) FRET analysis shows increased FRET efficiency in stretched cells, indicating reduced membrane tension. Scale bar: 5 µm. (*n *= 9). i,j) Atomic force microscopy (AFM) force indentation experiments reveal softening of the nucleus in the ST(+) group compared to ST(−). (*n *= 7). Statistical significance is denoted as *p < 0.5 and **p < 0.01, determined by Student's *t*‐test. All experiments were conducted right after 15 min of stretching.

To further interrogate how heterochromatin unpacks at the nuclear edges upon stretch, we visualized lamina‐associated domains (LADs) in heterochromatin using the m^6^A Tracer system^[^ ^39^, ^40^, ^41^, ^42^ ^]^ (**Figure** **2**a). Cells co‐transfected with Dam‐LMNB1 and m^6^A‐Tracer plasmids enable fluorescent labeling of LADs by introducing Dam‐mediated N^6^‐methyladenine (m^6^A) marks at GATC motifs within heterochromatic DNA regions located near the nuclear lamina. Upon stretch, m^6^A intensity was reduced at the nuclear periphery, directly demonstrating the loss of LAD‐associated heterochromatin (Figure 2b,c). These results support that H3K9me3, a major heterochromatin at the nuclear periphery, is significantly reduced by stretch.^[^ ^43^ ^]^ (Figure 1f). Examination of the nuclear periphery by transmission electron microscopy (TEM) also revealed the loss of LADs‐heterochromatin area in the stretched group (Figure 2d,e; Figure S8, Supporting Information). These nuclear envelope structural changes align with previous findings that mechanical stress triggers rapid and reversible responses at the nuclear envelope, including ATR relocalization to nuclear membranes to modulate nuclear envelope plasticity and chromatin association.^[^ ^44^ ^]^ The coordinated nuclear envelope remodeling observed in our study may represent a similar mechanosensitive checkpoint that enables cells to withstand mechanical strain while maintaining genomic integrity.

We further observed an increase in nuclear envelope (NE) wrinkling in stretched cells, implying a loss of nuclear membrane tension under stretch.^[^ ^28^, ^45^ ^]^ Furthermore, fluorescence resonance energy transfer (FRET)‐based nesprin‐2 tension sensor (Figure 2f) revealed a significant increase in FRET signal in stretched cells, indicating that perpendicular tension exerted by the cytoskeleton on the nuclear envelope was reduced by stretch (Figure 2g,h). A decrease in nuclear stiffness (≈50%), measured by atomic force microscopy, supported the loss of nuclear membrane tension (Figure 2i,j). The observed nuclear softening, despite increased perinuclear actin, can be attributed to the dominant influence of chromatin organization on nuclear mechanics. Specifically, the loss of H3K9me3, reduction of LADs‐heterochromatin, and increased nuclear envelope wrinkling collectively decrease nuclear stiffness to a greater extent than perinuclear actin can reinforce it, with the latter serving primarily as a protective scaffold.^[^ ^28^, ^46^ ^]^ Collectively, these findings demonstrate that cyclic stretch elicits coordinated nuclear responses, including peripheral heterochromatin unpacking, reduced cytoskeletal‐nuclear envelope tension, and overall nuclear softening.

### 2.3 Stretch Reinforces Perinuclear Actin Assembly Originating from Nuclear G‐Actin

**Figure 3:**
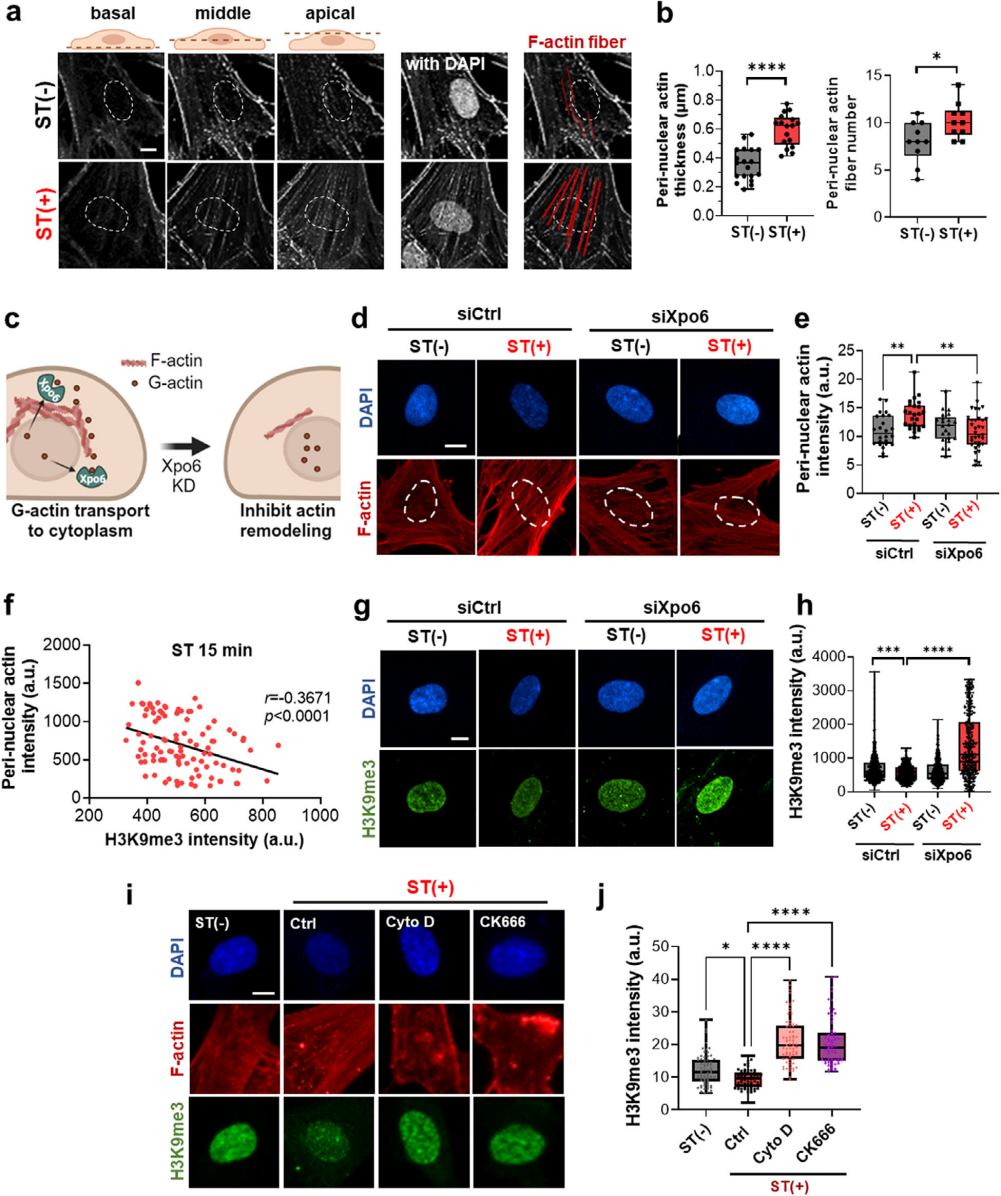
Nuclear‐to‐cytoplasmic actin redistribution mediates stretch‐induced perinuclear remodeling and H3K9me3 reduction. a) Confocal microscopy of phalloidin‐stained images at different cellular height levels (basal, middle, and apical level), demonstrating notable perinuclear actin formation upon stretch (Scale bar: 5 µm), and b) semi‐quantification of perinuclear actins in terms of fiber thickness and number at/near the nucleus, revealing increased fiber thickness (*n *> 18; ****p < 0.0001, Student's *t‐*test) and number (*n *= 10; *p < 0.05, Student's *t*‐test). c–e) Inhibition study of exportin‐6 (Xpo6) transport to the cytoplasm, demonstrating the stretch‐induced perinuclear actin formation is prevented; illustration depicting the role of Xpo6 in perinuclear actin remodeling in (c), phalloidin‐stained confocal images upon genetic silencing of Xpo6 (siXpo6) in (d), and semi‐quantification of perinuclear actin intensity in (e) (*n *> 25; **p < 0.01). Scale bars: 5 µm. f) Negative linear correlation between perinuclear actin intensity and H3K9me3 (Pearson's correlation coefficient *r *= −0.3671, *p *< 0.0001). g,h) Inhibition of perinuclear actin remodeling through Xpo6 silencing counteracts the stretch effects on H3K9me3 downregulation, as visualized by immunofluorescence‐stained H3K9me3 images in (g) and semi‐quantification in (h) (*n *> 400; ****p < 0.0001, ***p < 0.001). Scale bar: 5 µm. i) Immunofluorescence staining and j) semi‐quantification of H3K9me3 and F‐actin in cells treated with F‐actin polymerization inhibitor cytochalasin D (Cyto D) and Arp2/3 inhibitor CK666 (*n *= 70; *p < 0.05, ****p < 0.0001). Scale bar: 5 µm. For multiple groups comparison, one‐way ANOVA with Tukey's post hoc test.

To further investigate the mechanotransduction axis underlying perinuclear heterochromatin liberation and nuclear softening, we examined the dynamics of perinuclear filamentous actins (F‐actins). Recent studies have highlighted the structural and functional coupling between perinuclear actin networks and the nuclear envelope, with perinuclear F‐actins serving as key mediators of mechanical signal transmission to the nucleus.^[^ ^47^, ^48^, ^49^, ^50^ ^]^ Of note, we observed that perinuclear actin formation peaked at 7 min post‐stretch, preceding the maximal reduction in H3K9me3 at 15 min (Figure S9, Supporting Information), suggesting that actin remodeling may initiate upstream of stretch‐induced epigenetic changes. We conducted a detailed analysis of perinuclear actins by dissecting two regions: those overlapping with the DAPI area and those near the edge (a 0.5 µm perinuclear shell) (Figure S10a, Supporting Information). Stretch significantly increased perinuclear F‐actin levels in both regions (Figure S10b, Supporting Information), reinforcing the role of actin remodeling as a hallmark of cytoskeletal mechanosensing under mechanical loading.^[^ ^28^ ^]^ Further 3D imaging at basal, middle, and apical planes revealed that both the thickness and number of perinuclear actin fibers significantly increased upon stretch (**Figure** **3**a,b).

To understand how perinuclear actin is promptly formed upon stretch, we focused on the translocation of nuclear globular actins (G‐actins) to the cytoplasm (as depicted in Figure 3c), and investigated this process by genetically silencing exportin‐6 (Xpo6), a key nuclear export receptor that is specific for actin formation in a complex with profilin (Figure S11, Supporting Information).^[^ ^51^ ^]^ The depletion of Xpo6 resulted in the inhibition of perinuclear actin formation, suggesting that perinuclear actin formation in response to stretch utilizes nuclear G‐actins as a primary resource (Figure 3d,e). Given the short 15‐min timeframe, the increased perinuclear actin likely reflects redistribution rather than de novo synthesis of actin, indicating that nuclear actin depletion may be the primary driver of H3K9me3 reduction, with perinuclear actin accumulation serving as a readout of this nuclear export process. To establish a link between perinuclear actin formation and H3K9me3 loss under stretch, we performed a correlation analysis between them, observing a significant negative correlation between H3K9me3 and perinuclear actin formation (*r *= −0.3671) (Figure 3f). We further tested whether the inhibition of Xpo6 could influence the H3K9me3 level. The depletion of Xpo6 counteracted the stretch‐induced downregulation of H3K9me3, demonstrating the essential role of perinuclear actin formation in mediating H3K9me3 downregulation under stretched conditions (Figure 3g,h). Furthermore, when we blocked the F‐actin remodeling using cytochalasin D (Cyto D) and CK666, the stretch effect on decreasing H3K9me3 was profoundly inhibited (Figure 3i,j).

### 2.4 Stretch‐Induced Perinuclear Actin Formation and Epigenetic Change are Mediated by Immediate Calcium Release from the Endoplasmic Reticulum

**Figure 4:**
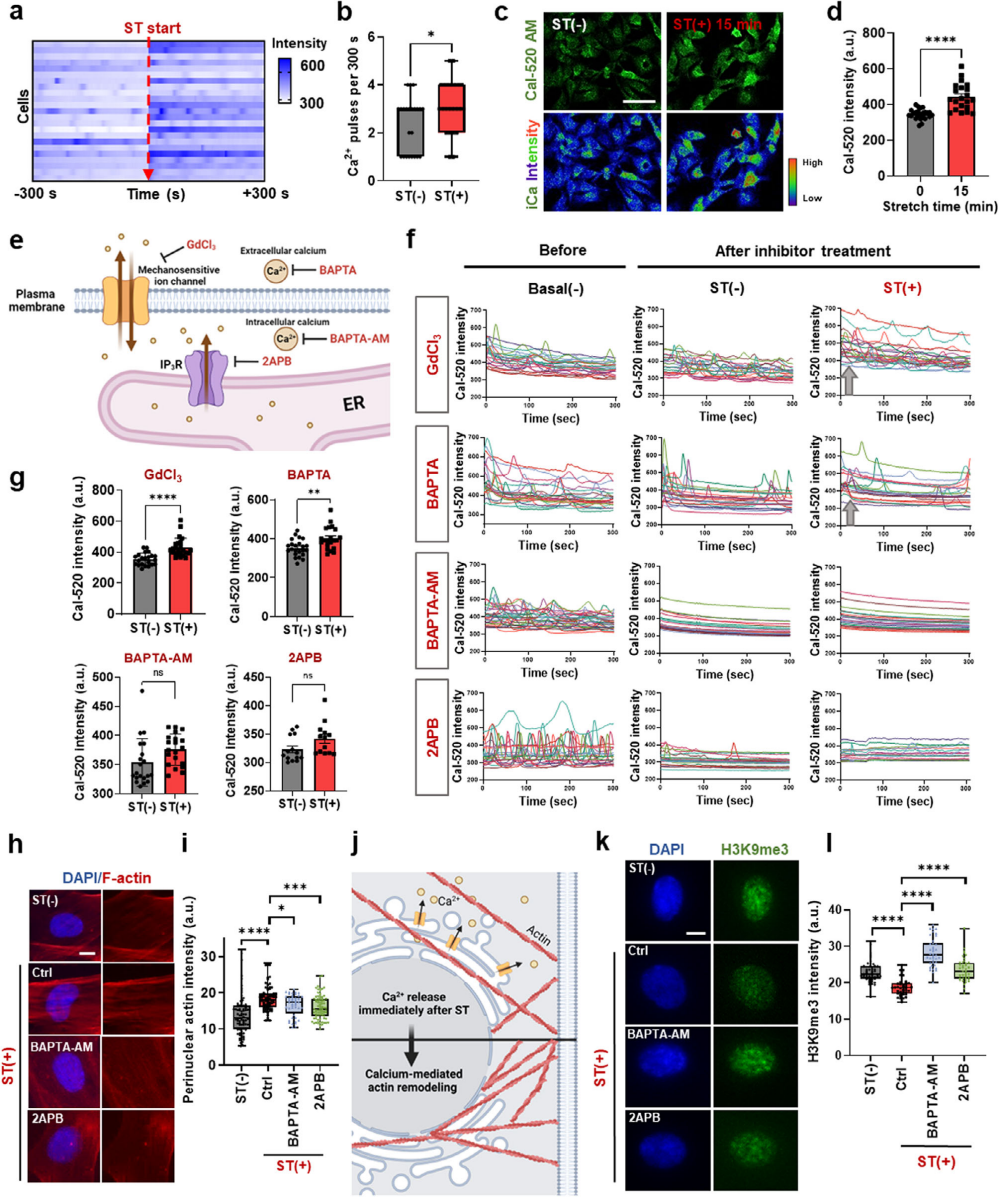
Stretch causes immediate calcium release from the endoplasmic reticulum, mediating perinuclear actin formation and epigenetic changes. a) Representative heatmap of intracellular Ca^2+^ sensor Cal‐520 AM live imaging intensity, showing increased Ca^2+^ intensity in stretched cells, and b) quantification of the heatmap intensity (n=23; *p < 0.05, Student's *t*‐test), revealing a significantly increased frequency of Ca^2+^ release under stretched conditions. c) Representative images of cells positive for Cal‐520 AM at 15 min, and d) quantification of the intensity, showing significantly increased Ca^2+^ intensity upon stretching (*n *> 23; ****p < 0.0001, Student's *t*‐test). Scale bar: 100 µm. e) Illustration of the actions of calcium channel inhibitors and Ca^2+^ chelators: stretch‐activated calcium channel blocker (gadolinium chloride, GdCl_3_), extracellular calcium chelator (BAPTA), intracellular calcium chelator (BAPTA‐AM), and inhibitor of IP3R in endoplasmic reticulum (ER) (2APB). f) Quantitative graphs of Cal‐520 AM live imaging before (Basal(−)) and after treatment of inhibitor prior to stretching (ST(−)), and followed by stretching under each inhibitor (ST(+)), and g) quantification of the intensity. Ca^2+^ pulses disappeared in the intracellular calcium inhibitor groups (BAPTA‐AM and 2APB), unlike the extracellular calcium inhibitor groups (GdCl3 and BAPTA, *n *> 20, **p < 0.01, ****p < 0.0001, Student's *t‐*test). h,i) Effect of the intracellular calcium inhibitor (BAPTA‐AM, 2APB) treatment on the perinuclear actin formation; DAPI (blue) and phalloidin (red) staining images in (h) and semi‐quantification in (i), showing reduced stretch‐induced perinuclear actin formation in the inhibitor groups (*n *> 77; *p < 0.5, ***p < 0.001, ****p < 0.0001). Scale bar: 5 µm. j) Illustration depicting that Ca^2+^ released from the ER after stretch mediates actin remodeling and plays a crucial role in stretch‐induced perinuclear actin formation. k,l) Effect of the intracellular calcium inhibitor (BAPTA‐AM, 2APB) treatment on the H3K9me3 change; DAPI (blue) and H3K9me3 (green) staining images in (k) and semi‐quantification in (l), revealing the stretch‐induced H3K9me3 decrease was significantly recovered with inhibitor treatment (*n *> 40; ****p < 0.0001). Scale bar: 5 µm. For multiple groups comparison, one‐way ANOVA with Tukey's post hoc test.

Considering the well‐established role of intracellular calcium ions (Ca^2+^) in response to mechanical stress and their connections to actin remodeling,^[^ ^52^, ^53^, ^54^, ^55^, ^56^ ^]^ we hypothesized that calcium ions might be involved in the stretch‐induced events around the nuclear space. To prove this, we live‐imaged cells using the intracellular Ca^2+^ sensor Cal‐520 AM. The heatmap of live imaging intensity showed a significant increase in Ca^2+^ intensity under stretched conditions (**Figure** **4**a). Quantitative analysis revealed a significant increase of Ca^2+^ pulses upon stretching (Figure 4b; Figure S12, Supporting Information). Representative images of cells positive for Cal‐520 AM at 15 min and the quantification of the intensity further confirmed significantly increased Ca^2+^ intensity upon stretching (Figure 4c,d).

To identify the source of the calcium increase, we treated cells with various inhibitors targeting calcium influx and release pathways, as illustrated in Figure 4e. These included: stretch‐activated calcium channel blocker (gadolinium chloride, GdCl_3_), extracellular calcium chelator (BAPTA), intracellular calcium chelator (BAPTA‐AM), or inhibitor of endoplasmic reticulum (ER)‐resident inositol 1,4,5‐triphosphate receptors (IP3Rs) (2APB). Live imaging of Cal‐520 AM signals (Figure 4f) and intensity quantification (Figure 4g) before and after treatment showed that inhibitors of extracellular calcium influx (GdCl_3_, BAPTA) did not prevent stretch‐induced Ca^2^⁺ elevation. In contrast, blocking intracellular calcium release from the ER with 2‐APB or chelating intracellular calcium with BAPTA‐AM abolished Ca^2^⁺ pulses in response to stretch (Figure 4f,g). These results indicate that the stretch‐induced increase in intracellular calcium is both immediate and primarily mediated by ER calcium release.

We next investigated whether this calcium signaling cascade is necessary for perinuclear actin formation and associated epigenetic changes. Inhibition of intracellular calcium signaling (via BAPTA‐AM or 2‐APB) significantly reduced perinuclear F‐actin assembly under stretch conditions (Figure 4h,i), suggesting that calcium release is required for actin remodeling (Figure 4j). In addition, stretch‐induced downregulation of H3K9me3 was markedly attenuated by calcium inhibition, indicating that epigenetic remodeling is similarly dependent on ER‐mediated calcium signaling (Figure 4k,l). Thus, we can establish a mechanistic sequence that cyclic stretch triggers immediate calcium release from the ER, which in turn promotes perinuclear actin assembly and epigenetic reprogramming through H3K9me3 downregulation.

### 2.5 Emerin Dynamics at the Nuclear Envelope Links Perinuclear Actin Assembly to Heterochromatin Unpacking upon Stretch

**Figure 5:**
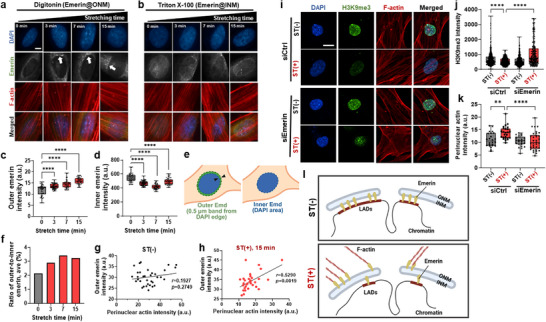
Emerin translocation at the nuclear envelope connects the events of perinuclear actin formation and heterochromatin liberation upon stretch. a,b) Immunofluorescence staining images of emerins at the outer nuclear membrane (ONM) and inner nuclear membrane (INN), respectively at different timepoints during stretch; emerins accumulated at the ONM are visualized via digitonin‐permeabilization in which the INM is inaccessible to antibodies, whereas those at the INM are identified via Triton‐X‐permeabilization. Immunostaining was performed after fixation at the indicated stretch timepoints. Scale bar: 5 µm. White arrow indicating distinguished ONM over‐stretching time. c,d) Semi‐quantification of emerin intensities at the ONM and INM, respectively, recorded over stretch periods of up to 15 min, along with e) the schematic showing the analysis method (ONM emerin: *n *= 47; ****p < 0.0001 and INM emerin: *n *= 53; ****p < 0.0001). f) The ratio of emerin intensities at ONM‐to‐INM shows a gradual accumulation at ONM at the expense of INM. g,h) A linear correlation is noted between ONM emerin intensity and perinuclear actin intensity in stretched cells (Pearson's correlation value; in 0 min graph, *r *= 0.1927 and *p *= 0.2749 versus in 15 min graph, *r *= 0.5290 and *p *= 0.0019). i–k) Effects of emerin depletion on perinuclear actin formation and H3K9me3 change; immunofluorescence staining images of H3K9me3 and F‐actin with (siEmerin) or without (siCtrl) genetic silencing of emerin (shown in (i)), revealing that depletion of emerin abolishes the stretch effects on H3K9me3 decrease (shown in (j)) and perinuclear actin formation (shown in (k)). Scale bar: 10 µm. (for H3K9me3, *n *> 300, ****p < 0.0001, and for perinuclear actin, *n *> 25; **p < 0.01, ****p < 0.0001). l) Illustration showing the stretch‐induced accumulation of emerins at ONM at the expense of those at INM, leading to the liberation of LADs‐heterochromatin, mainly H3K9me3, and concurrently forming a complex with perinuclear actins. For multiple groups comparison, one‐way ANOVA with Tukey's or Dunnett's post hoc test.

The concurrent events of actin remodeling at the nuclear periphery and heterochromatin change prompted us to consider the emerin molecule as the possible mediator, given its role as a key mechanosensitive LINC complex protein. Emerin bridges the cytoskeleton and LADs in heterochromatin as a communication channel^[^ ^16^, ^57^, ^58^ ^]^ by translocating between the inner nucleus membrane (INM) and outer nucleus membrane (ONM). To visualize emerins at ONM and INM separately, we employed digitonin‐permeabilization for emerins at ONM where the INM is inaccessible to antibodies, while those at the INM were identified via Triton‐X‐permeabilization (**Figure** **5**a,b).^[^ ^16^ ^]^ Semi‐quantitative analysis of emerin localization was performed over a 15‐min stretching period in regions corresponding to the inner nuclear membrane (INM, within DAPI‐stained nucleus) and outer nuclear membrane (ONM, within 0.5 µm from the nuclear periphery) (Figure 5c–e). Of note, emerin progressively accumulated at the ONM, with a corresponding decrease at the INM, beginning as early as 3 min post‐stretch (Figure 5c,d). The ONM‐to‐INM emerin intensity ratio further confirmed this redistribution (Figure 5f). This stretch‐induced translocation of emerin to the ONM positively correlated with perinuclear actin intensity at 15 min (Figure 5g,h), indicating a spatial relationship between emerin redistribution and cytoskeletal remodeling at the nuclear periphery during mechanical stress.^[^ ^28^ ^]^

To functionally assess the role of emerin in mediating perinuclear actin assembly and chromatin remodeling, we performed genetic silencing of emerin. Knockdown of emerin abolished the stretch‐induced increase in perinuclear actin and prevented the reduction of H3K9me3 (Figure 5i–k). These results establish that emerin is essential for the coordination of cytoskeletal and chromatin responses to mechanical stretch. Taken together, our findings demonstrate that stretch‐induced translocation of emerin from the INM to the ONM mediates two key processes: 1) the release of LAD‐associated heterochromatin, particularly H3K9me3, and 2) the recruitment and stabilization of perinuclear actin structures. These results position emerin as a critical mechanotransducer linking nuclear envelope remodeling to cytoskeletal and epigenetic reprogramming during mechanical stress (Figure 5l).

### 2.6 Genome‐Wide Profiling Reveals Stretch‐Induced Changes in Chromatin Accessibility

To determine which genomic regions are affected by stretch‐induced heterochromatin loss, we performed ATAC‐seq (assay for transposase‐accessible chromatin followed by sequencing) to profile genome‐wide chromatin accessibility.^[^ ^59^ ^]^ The data showed characteristic fragment size distributions with periodic peaks at ≈100, 200, and 400 bp, and high enrichment at transcription start sites, confirming successful ATAC‐seq execution (Figure S14, Supporting Information). Notably, 20% more ATAC‐seq peaks were detected in the stretched group compared to the control (Figure S15, Supporting Information), consistent with a shift toward euchromatin.

**Figure 6:**
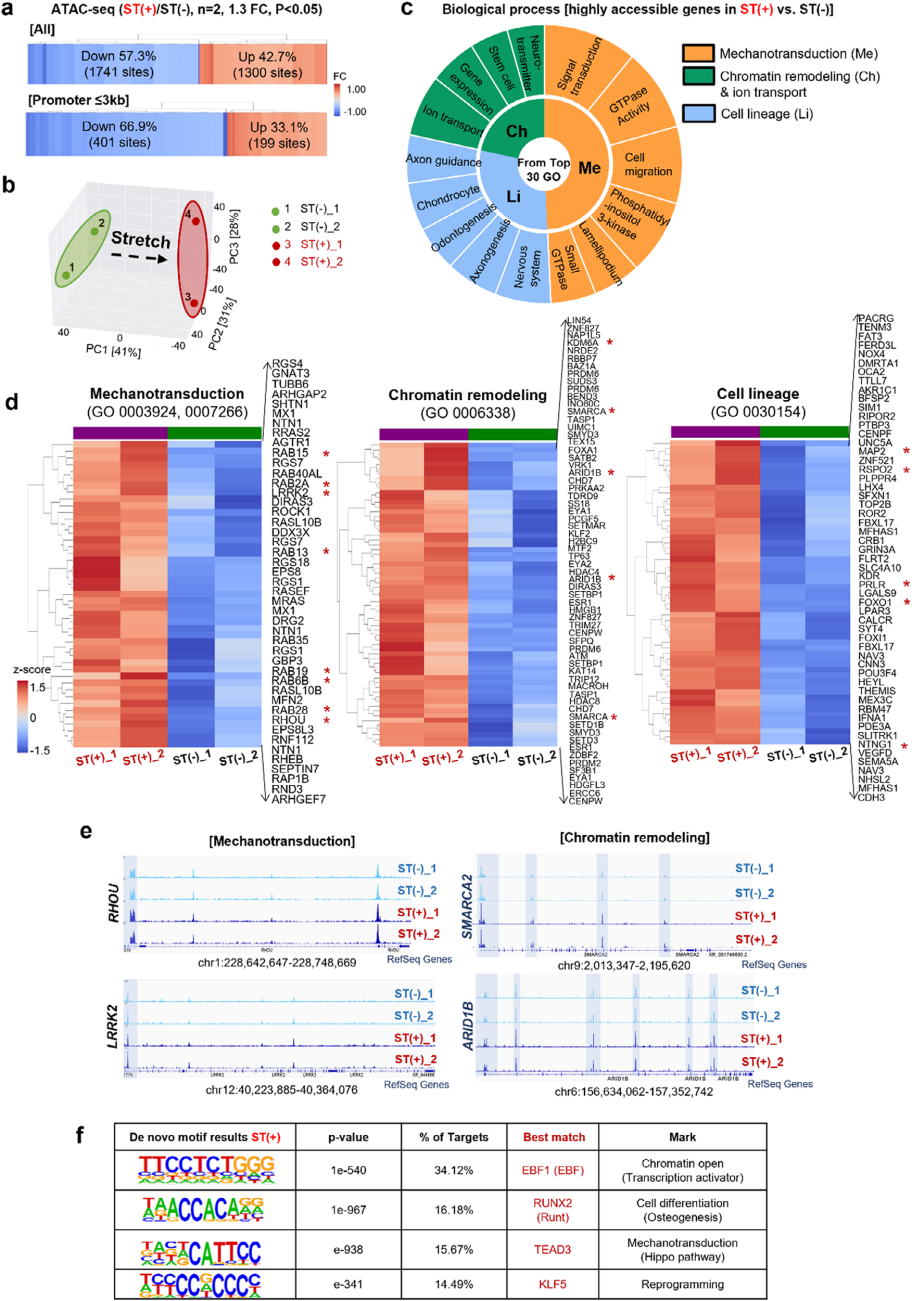
Analysis of open chromatin regions by ATAC‐sequencing reveals increased accessibility of genomic loci linked to mechanotransduction, chromatin remodeling, and multipotency. a) Heatmap of chromatin accessibility profiles, showing differences greater than 1.3‐fold between ST(−) and ST(+) (*n *= 2, 1.3 FC, *p *< 0.05). b) Principal component analysis (PCA) of chromatin accessibility was measured by ATAC‐seq in 4 samples, indicating that the largest variance in this dataset is caused by stretch. c) Gene ontology (GO) analysis of biological processes associated with upregulated differentially accessible regions in ST(+), identifying pathways related to mechanotransduction, cell lineage, and chromatin opening (and ion transport). d) Heatmap analysis of GO terms related to mechanotransduction, cell lineage, and chromatin remodeling, showing increased accessibility in ST(+) compared to ST(−) (1.2 FC). Representative genes are highlighted with asterisks. e) Representative transcription factor genome browser tracks of regions associated with mechanotransduction (*RHOU*, *LRRK2*) and chromatin remodeling (*SMARCA2*, *ARID1B*), showing higher accessibility in ST(+) versus ST(−). f) HOMER de novo motif prediction of ST(+) regions enriched for chromatin opening, cell differentiation, mechanotransduction, and reprogramming.

Genomic annotation revealed a similar distribution of accessible regions in both groups: over half of the peaks were located in enhancer regions (distal intergenic and intronic), and ≈23% in promoter regions (Figure S15, Supporting Information). A heatmap of differential chromatin accessibility identified 3041 total regions and 600 promoter‐associated regions with a fold change > 1.3 between stretched and unstretched conditions (**Figure** **6**a). Surprisingly, 33–43% of the increased accessibility occurred in previously unannotated regions, potentially reflecting novel regulatory elements activated by mechanical cues.^[^ ^60^ ^]^ Principal component analysis consistently demonstrates a significant alteration in chromatin accessibility in stretched cells (Figure 6b).

Gene ontology (GO) and KEGG pathways analysis (DAVID (v2022q3)) associated with the upregulated “differentially accessible regions” in stretched cells revealed enrichment of mechanotransduction (including signal transduction and positive regulation of GTPase activity), multipotency to different cell lineages, and chromatin opening and calcium transport (Figure 6c; Figure S16, Supporting Information).^[^ ^61^ ^]^ Heatmap analysis further identified more accessible genes related to mechanotransduction (*RHOU*, *LRRK2*, *RAB* family), chromatin remodeling (*SMARCA*, *ARID1B*, *KAT14*, *KDM6A*), and cell lineage (*MAP2*, *NTNG1*, *Foxo1*, *RSPO2*, *KDR*) under stretch (Figure 6d). Genome browser tracks confirmed increased accessibility of representative gene regions associated with mechanotransduction (*RHOU* and *LRRK2*, mediating actin cytoskeleton regulation as Ras family) and chromatin remodeling (*SMARCA2* and *ARID1B* that modulate chromatin remodeling complex for euchromatin structure as *SWI/SNF* family) (Figure 6e). Motif analysis of differentially accessible regions revealed enrichment of transcription factors such as *EBF1*, *RUNX2*, *TEAD3*, and *KLF5*, which are associated with chromatin remodeling, mechanotransduction, and lineage differentiation (Figure 6f).^[^ ^62^, ^63^ ^]^

To assess whether stretch‐induced chromatin remodeling might prime fibroblasts for alternative cell fates, we examined the spatial localization of lineage‐specific genes using oligonucleotide fluorescence in situ hybridization (FISH). S100A4, a marker of fibroblast activation, was more frequently localized to the nuclear periphery in stretched cells, a positioning typically associated with gene repression^[^ ^64^ ^]^ (Figure S17, Supporting Information). However, *FUT4*, a stemness‐associated gene, did not show significant regional differences. This raises the intriguing possibility that mechanical stretch may repress fibroblast‐specific gene programs while enhancing accessibility at loci related to multipotency and other lineage identities.

Indeed, GO analysis of the terms related to ossification, cartilage, and muscle cell differentiation explained well the stretched cells exhibited more accessible regions than unstretched cells (Figure S18, Supporting Information). However, targeted qPCR analysis at 15 min post‐stretch (Figure S19a, Supporting Information) showed that lineage‐specific gene expression changes were limited without additional biochemical cues (Figure S19b, Supporting Information), indicating that chromatin accessibility alone is insufficient for lineage specification. Based on this, we further tested if the stretched fibroblasts could exhibit the traits of other lineage cells with proper biochemical cues. Cells were cultured in specific‐lineage differentiation medium for 3 days under stretching for 15 min per day (Figure S19a, Supporting Information). Of note, the lineage‐specific (neuro‐, myo‐, osteo‐genic) genes were significantly upregulated by the stretch (Figure S19c, Supporting Information). Consistent with ATAC‐seq findings showing increased accessibility at neural‐specific genes such as *LHX4* and *ADCY1* (Figure S20a, Supporting Information), we observed elevated expression of *Tuj1*, an early neuronal marker, in stretched fibroblasts by day 5 (Figure S20b, Supporting Information).

These findings reveal that cyclic stretching of fibroblasts induces a genome‐wide, transient increase in chromatin accessibility in fibroblasts, particularly at loci associated with alternate lineage potential and chromatin remodeling. Simultaneously, genes related to fibroblasts are repressed. These findings suggest that mechanical stimuli can reprogram fibroblasts toward a more plastic or regenerative state, potentially contributing to tissue repair.^[^ ^65^, ^66^ ^]^ Furthermore, these results align with recent studies showing that cyclic stretching enhances the efficiency of fibroblast reprogramming into pluripotent cells.^[^ ^15^ ^]^

### 2.7 Stretch‐Induced Perinuclear Mechanosensing Protects the Genome from DNA Damage

**Figure 7:**
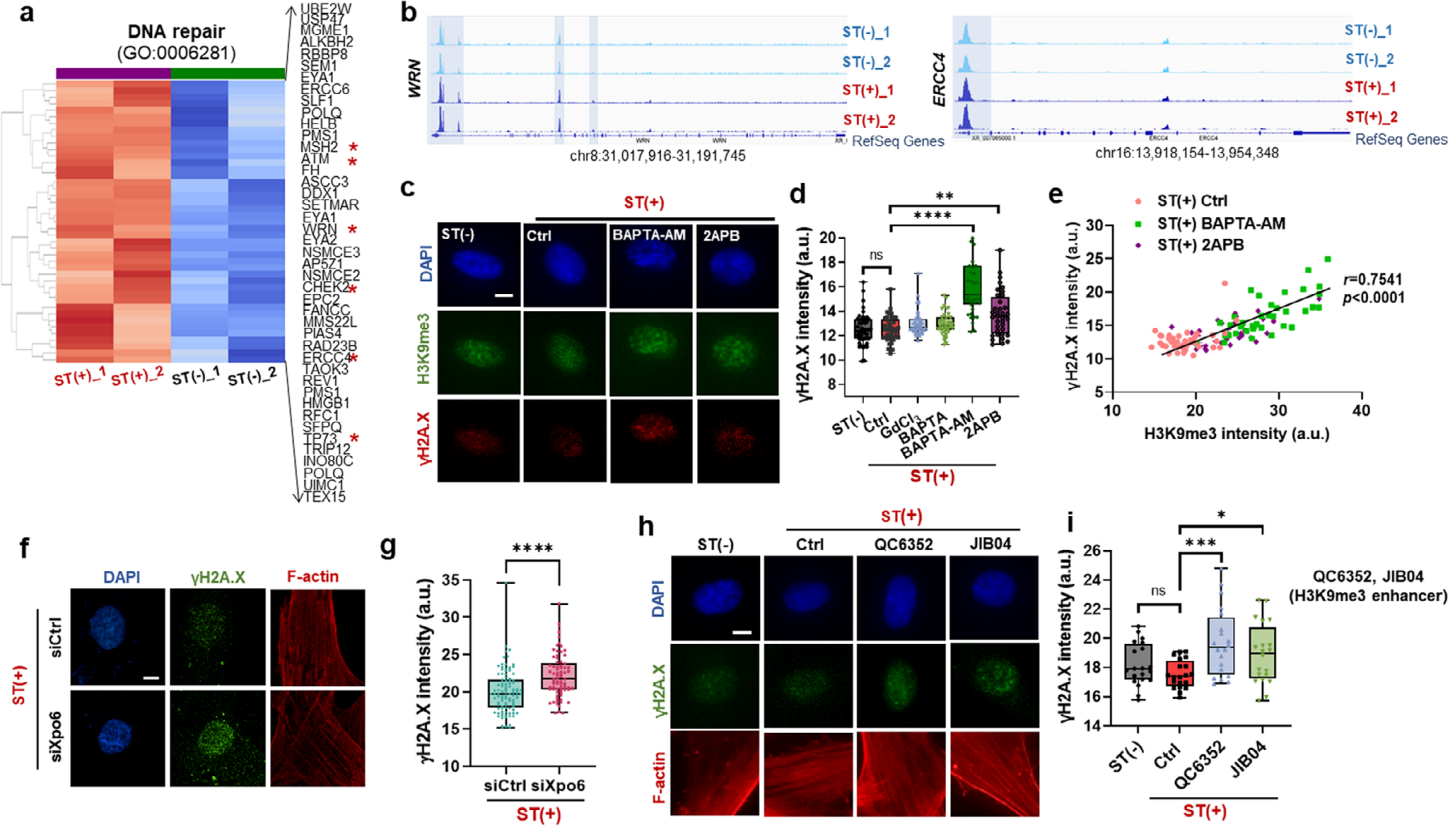
Stretch‐induced perinuclear mechanosensing events protect the genome from DNA damage. a) Heatmap analysis of GO terms related to DNA repair shows that stretched cells (ST(+)) exhibit more accessible regions compared to unstretched cells (ST(−)). b) Representative transcription factor genome browser tracks for DNA repair genes (*WRN, ERCC4*), demonstrating higher accessibility in ST(+) versus ST(−). c,d) Effects of blocking intracellular calcium release on DNA damage: immunofluorescence staining images of H3K9me3 and γH2A.X in (c), and semi‐quantification of γH2A.X intensity in (d), revealing that blocking intracellular calcium release (BAPTA‐AM, 2‐APB) induces DNA damage under stretch. Scale bar: 5 µm. (*n *> 40; **p < 0.01, ****p < 0.0001, by one‐way ANOVA with Tukey's post hoc test). e) A linear correlation is noted between H3K9me3 and γH2A.X intensity (Pearson's correlation value *r *= 0.7541, *p *< 0.0001). f,g) Effects of Xpo6 depletion on DNA damage: immunofluorescence staining images of γH2A.X and F‐actin in (f), and semi‐quantification of γH2A.X intensity in (g), showing that RNAi‐mediated depletion of Xpo6 inhibits stretch‐induced perinuclear actin formation and causes DNA damage. Scale bar: 5 µm. (*n *> 78; ****p < 0.0001, by Student's *t*‐test). h,i) Effects of H3K9me3 overexpression on DNA damage: immunofluorescence staining images of γH2A.X and F‐actin in (h), and semi‐quantification of γH2A.X in (i), showing stretch‐induced DNA damage in the group with overexpressed H3K9me3 following treatment with H3K9me3 enhancers (QC6352 and JIB04, histone lysine demethylase inhibitors). Scale bar: 5 µm. (*n *= 20; *p < 0.05, ***p < 0.001, by one‐way ANOVA with Tukey's post hoc test).

As shown by our ATAC‐seq data, mechanical stretch induces marked changes in chromatin accessibility, particularly in the perinuclear region. One significant implication of these changes is their role in protecting the genome from mechanical damage. Previous studies have reported that epidermal stem and progenitor cells undergo heterochromatin loss and nuclear softening in response to mechanical strain (5–40%), serving as a protective adaptation against nuclear damage.^[^ ^28^ ^]^ From our ATAC‐seq analysis, we noticed increased accessibility of key genes related to DNA repair (*WRN*, *ERCC4*, *CHEK2*, *ATM*, *MSH2*, *TP73*, etc.; **Figure** **7**a,b) and DNA damage response (*TP63*, *FoxP1*, *Foxo1*, *RAD23B*, etc.; Figure S21, Supporting Information) upon stretch, hinting at a new role of H3K9me3 loss in stretched fibroblasts, which prompted us to look into the DNA damage and repair mechanisms more deeply.

As evidenced by the expression of DNA damage marker of γH2A.X, the 10% stretch did not induce any detectable genomic damage in fibroblasts (Figure 7c), implying the proper homeostatic actions of cells by means of liberating H3K9me3 while remodeling perinuclear actins. We further tested if cellular protection mechanisms would work properly when the cellular homeostatic actions were interrupted. When the increase of intracellular Ca^2+^ was interrupted via BAPTA‐AM and 2APB – both of which do not directly relate to the regulation of extracellular Ca^2+^ (GdCl_3_, BAPTA), DNA damage was noticed upon stretch (Figure 7c,d; Figure S22, Supporting Information), suggesting the intracellular Ca^2+^ increase is an essential cellular homeostatic event to prevent DNA damage.

We found a strong positive correlation between H3K9me3 levels and DNA damage under stretch (*r* = 0.7541; Figure 7e), suggesting that loss of heterochromatin is protective. To explore the contribution of perinuclear actin, we depleted exportin‐6 (Xpo6) via siRNA to prevent nuclear G‐actin export and perinuclear F‐actin formation. Xpo6 knockdown resulted in significantly elevated γH2A.X signals upon stretch (Figure 7f,g), reinforcing the notion that actin remodeling is critical for genome protection. Next, we tested whether chemically preventing H3K9me3 loss using histone demethylase inhibitors (JIB04 and QC6352; Figure S23, Supporting Information) would compromise DNA integrity under stretch. Indeed, pharmacological inhibition of H3K9me3 demethylation led to increased γH2A.X expression (Figure 7h,i). Importantly, these treatments also abrogated perinuclear actin formation under stretch (Figure S24, Supporting Information), demonstrating that heterochromatin loss and actin remodeling are interdependent processes necessary for maintaining genomic stability during mechanical stress.

**Figure 8:**
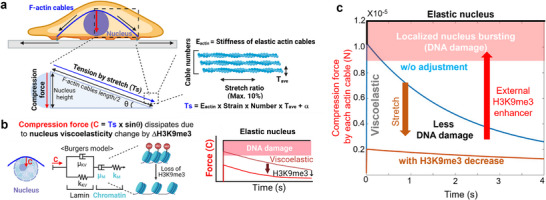
Physical model of F‐actin‐derived compression force on viscoelastic nucleus under stretch inducing DNA damage. a) Physical model of compression force on the nucleus exerted by F‐actin cables and its dissipation in a viscoelastic nucleus with changes in chromatin status. The model assumes that elastic F‐actin cables form a cap above the nucleus and are anchored to the cell membrane and substrate at both ends. As the substrate is stretched, the F‐actin cables elongate, leading to increased tension and compression forces on the nucleus. The above compression force to the nucleus under stretch could be calculated by F‐actin cables’ number and their thickness. b) The viscoelastic properties of the nucleus, described by the 4‐element Burgers model, allow for compression force dissipation. c) Model prediction shows that both elastic and viscoelastic nuclei without physical adjustment experience DNA damage immediately after stretch‐induced compression forces. The loss of H3K9me3 alters the viscoelastic properties of the nucleus, enabling compression force dissipation below the localized nuclear bursting force, thus preventing DNA damage. Blocking H3K9me3 loss with external chemicals under stretch leads to detectable DNA damage. See also Methods S1,S2 (Supporting Information).

We next developed a theoretical model and performed computational simulations^[^ ^67^, ^68^ ^]^ to elucidate the role of F‐actin cables in transmitting stretch‐induced compression forces to the nucleus and the viscoelastic properties of the nucleus in dissipating these forces to prevent DNA damage (**Figure** **8**). The model assumes that elastic F‐actin cables form a cap above the nucleus and are anchored to the cell membrane and substrate at both ends (Figure 8a). As the substrate stretches, the elastic F‐actin cables elongate, increasing tension and compression forces on the nucleus. Simulations were performed across a range of strains, relative F‐actin cable numbers, and cable thicknesses. The results, visualized with 3D surface plots, identify F‐actin cable parameters that likely amplify stretch‐induced compression force, potentially causing DNA damage without a feedback response from the nucleus^[^ ^69^, ^70^ ^]^ (Methods S1,S2, Supporting Information). Next, we applied a viscoelastic model to accurately predict the nucleus behaviors using the 4‐element Burgers model, allowing for compression force dissipation^[^ ^71^ ^]^ (Figure 8b, Method S2, Supporting Information). Both elastic and viscoelastic nuclei without additional physical alteration by chromatin feedback changes are predicted to experience DNA damage immediately after stretch‐induced compression forces. However, the loss of H3K9me3 alters the viscoelastic properties of the nucleus, enabling compression force dissipation below the threshold for nuclear bursting, thus preventing DNA damage (Figure 8c). This finding aligns with our experimental observations, where blocking H3K9me3 loss with external chemicals under stretch resulted in detectable DNA damage. In summary, our computational model provides a mechanistic framework for understanding how chromatin modifications protect the genome against mechanical damage under stretch through perinuclear actin remodeling.^[^ ^52^ ^]^ While our current model focuses on immediate mechanosensing responses occurring within 15 min of stretch, we acknowledge that cells can undergo geometric adaptation over longer time periods. As observed in Figure 1b, cells eventually align perpendicular to the stretch direction, which likely minimizes F‐actin cable length and associated compression forces on the nucleus. This cellular reorientation represents a secondary adaptive mechanism that could transit from the immediate mechanosensing response characterized here to longer‐term cellular adaptation, and warrants consideration in future comprehensive modeling approaches.

### 2.8 Physiological Stretch in Skin Tissue Triggers Nuclear Mechanosensing via Actin Recruitment and Heterochromatin Remodeling

To determine whether stretch‐induced perinuclear mechanosensing observed in vitro also occurs in vivo, we examined mouse dermal fibroblasts under conditions of differential physiological mechanical tension. A surgical undermining model was employed to reduce mechanical tension in localized areas of skin (“less‐ST”) by cutting the attachments between the skin and underlying muscle tissues, compared to regions under normal physiological stretch (“ST”) with intact tissue attachments (**Figure** **9**a; Figure S25, Supporting Information). This surgical undermining procedure, commonly used in clinical practice to reduce skin tension during wound closure, effectively created differential mechanical environments for comparative analysis within the same animal.^[^ ^72^ ^]^ Specifically, we made a midline dorsal skin incision and performed either undermining (less‐ST) or a sham procedure (ST) on randomly assigned left and right sides before closing with sutures, with both conditions analyzed simultaneously after 24 h to eliminate temporal variables. After 24 h, fibroblasts from physiological stretched regions exhibited significantly reduced H3K9me3 levels compared to those from less‐ST regions (Figure 9b). Consistent with our in vitro findings, fibroblasts in stretched dermis exhibited increased total and perinuclear F‐actin accumulation without notable changes in nuclear size or morphology (Figure 9c,d; Figures S26,S27, Supporting Information). Furthermore, a significant negative correlation was observed between perinuclear actin levels and H3K9me3 expression (Figure 9e), with a similar trend noted for nuclear‐localized F‐actin (Figure S28, Supporting Information).

Figure 9In vivo skin fibroblasts under stretching exhibit H3K9me3 loss with increased perinuclear actin compared to non‐stretched counterparts. a) Schematic showing the in vivo experimental design. Mouse dorsal skin fibroblasts were analyzed from two groups within the same animal: normal, physiologically stretched skin (“ST”) and less‐stretched skin (following surgical incision detaching skin from muscle: “Less‐ST”) with random assignment of ST and Less‐ST conditions to left and right sides. b) H3K9me3 expression levels. Immunofluorescence staining for H3K9me3 (magenta) and DAPI (blue) with semi‐quantification of H3K9me3 intensity, revealing significantly reduced levels in ST fibroblasts compared to Less‐ST (*n *= 30). c) F‐actin expression levels. Immunofluorescence staining for F‐actin (green) and semi‐quantification normalized to the number of nuclei, showing significantly increased F‐actin in ST fibroblasts compared to Less‐ST (*n *= 13). d) Perinuclear actin expression. Analysis of perinuclear actin (within a 1 µm boundary from the nucleus), demonstrating significantly increased levels in ST compared to Less‐ST fibroblasts (*n *= 30). e) Correlation analysis. A moderate negative correlation was observed (*r* = ‐0.6264) between perinuclear actin intensity and H3K9me3 expression. f) Pharmacological enhancement of H3K9me3. Treatment with the H3K9me3 enhancer JIB04 (20 mg kg^−1^) led to a significant increase in H3K9me3 levels in fibroblasts compared to vehicle control (dimethyl sulfoxide, DMSO), as visualized by immunofluorescence staining (*n *= 210). g) Effect on DNA damage. Immunostaining for γH2AX (magenta) showed that forced upregulation of H3K9me3 via JIB04 treatment was associated with increased DNA damage in fibroblasts (*n *= 210). h) Correlation analysis. A strong positive correlation was observed (*r* = 0.8181) between γH2AX and H3K9me2/3 intensity (refer to Figure S29, Supporting Information). i) Spatial transcriptomics workflow. Schematic showing the spatial transcriptomic analysis of in vivo dermal fibroblasts using GeoMx technology. A high magnification image is also provided in Figure S30 (Supporting Information). j) Principal component analysis (PCA). PCA revealed distinct transcriptional states between ST and Less‐ST fibroblasts. k) Gene ontology (GO) analysis. GO analysis of differentially expressed genes indicated significant enrichment of chromatin organization and actin cytoskeleton‐related processes in ST versus Less‐ST fibroblasts. DNA damage‐related processes were enriched in JIB04‐treated (ST+JIB04) versus DMSO‐treated ST fibroblasts (fold change > 1.3, normalized log2 > 2, *p *< 0.05, *n *= 3). Exclusions: Immune cells (CD11b+) and strong α‐SMA‐positive cells (myofibroblasts/myocytes) were excluded from dermal fibroblast analysis. Color coding: Green: α‐SMA, Blue: DNA, Yellow: CD11b, Red: H3K9me3. (*p < 0.05, ****p < 0.0001, by Student's *t‐*test).

**Figure:**
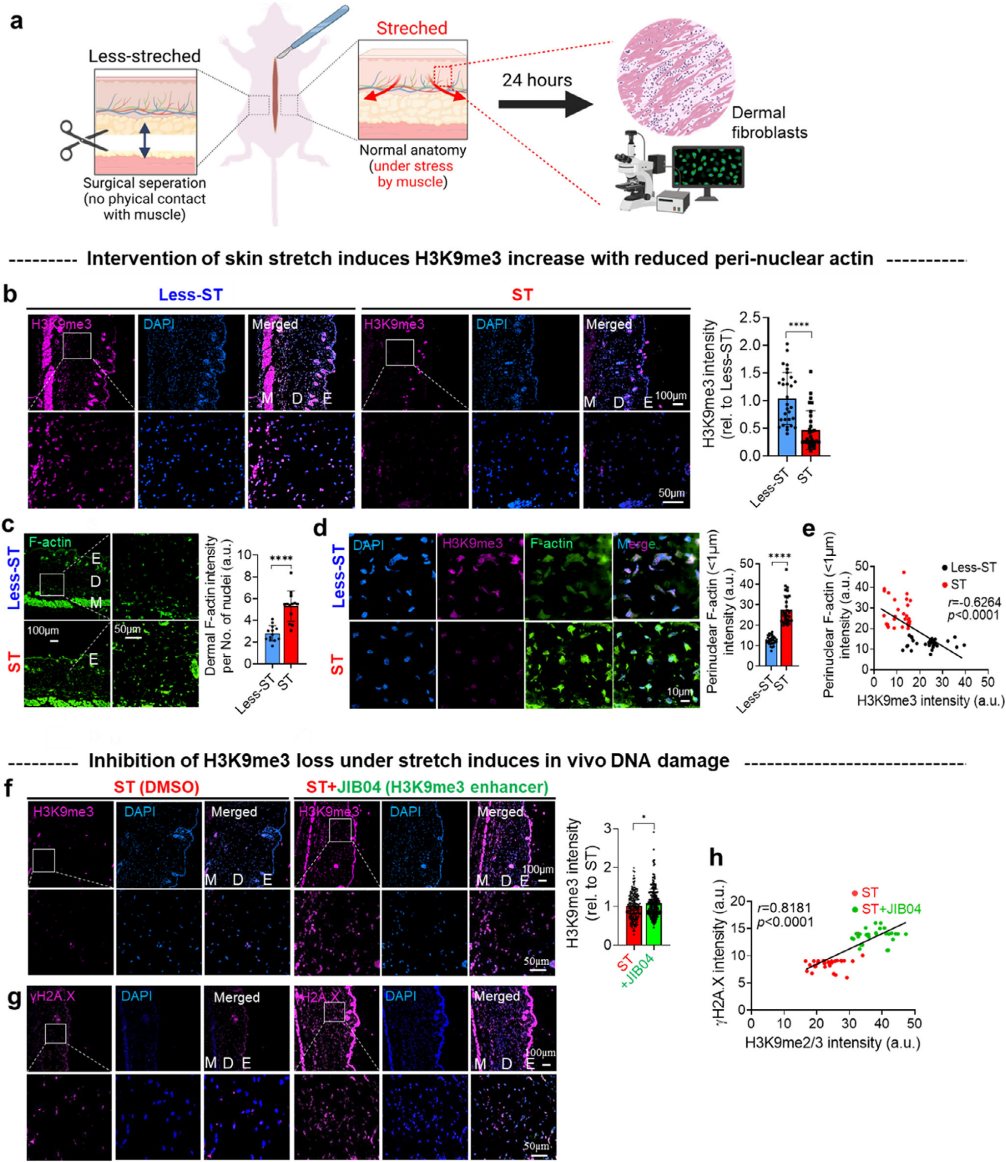

**Figure:**
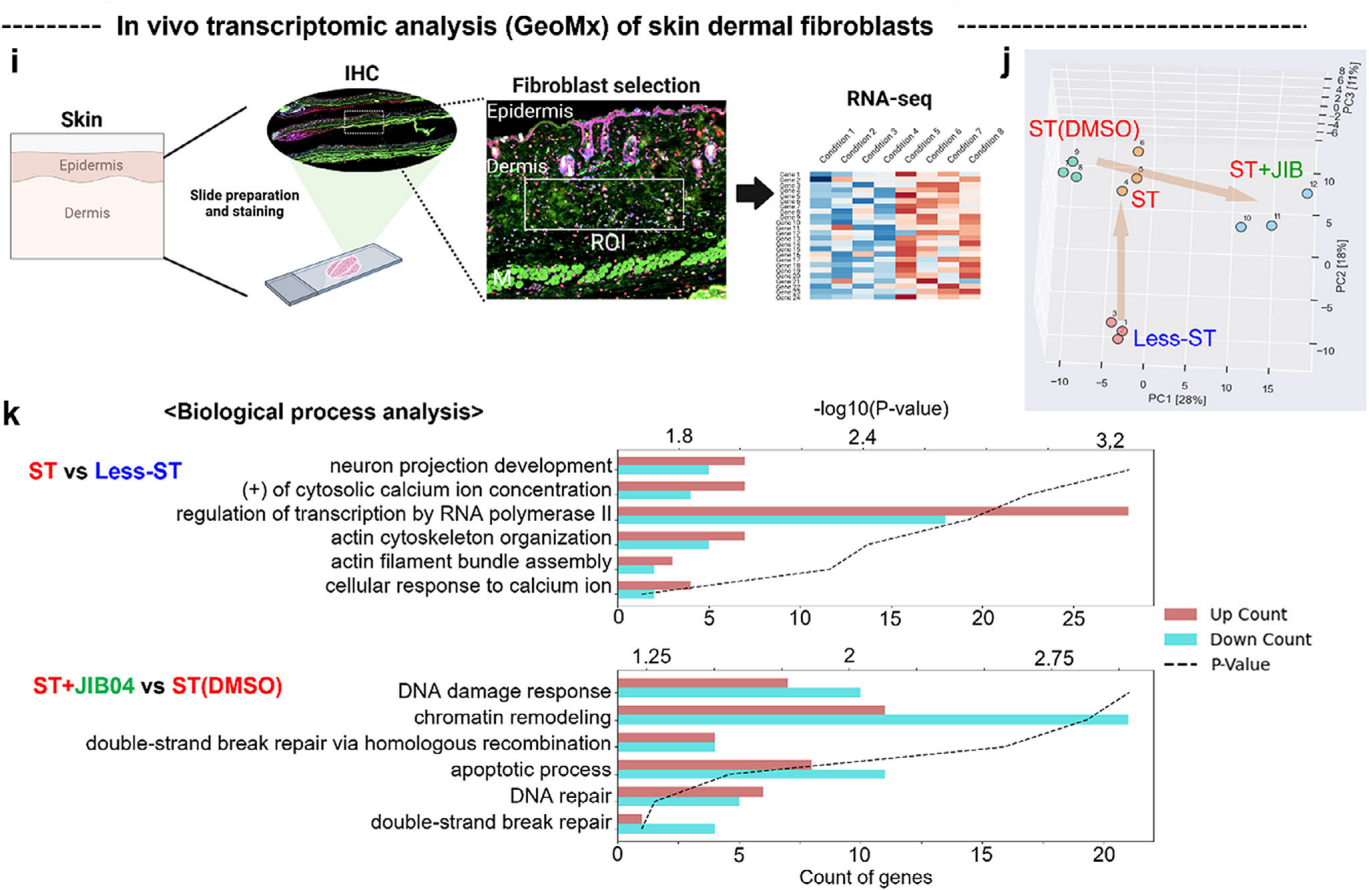

To assess the functional significance of H3K9me3 downregulation under stretch conditions, we treated the dermis with the H3K9me3 enhancer JIB04. This pharmacological intervention prevented stretch‐induced H3K9me3 loss, but significantly increased the expression of the DNA damage marker γH2AX compared to DMSO control (Figure 9f,g; Figure S29, Supporting Information). These results establish a strong positive correlation between perinuclear actin accumulation and H3K9me3 levels in vivo (Figure 9h), reinforcing our in vitro observations where cyclic stretch initiates a nuclear mechanotransduction cascade that mitigates DNA damage through chromatin remodeling and cytoskeletal adaptation.

To uncover genome‐wide molecular signatures associated with these responses, we conducted spatial transcriptomic profiling of dermal fibroblasts using GeoMx technology (Figure 9i). Cell‐type specificity was ensured by excluding immune (CD11b⁺) and myofibroblast/myocyte (α‐SMA⁺) lineages (Figure S30, Supporting Information). Principal component analysis revealed distinct transcriptomic signatures between stretched and less‐stretched dermal regions (Figure 9j). Gene ontology analysis of differentially expressed genes indicated significant enrichment of pathways associated with chromatin organization and actin cytoskeleton regulation in the stretched dermis (Figure 9k; Figure S31, Supporting Information). Key upregulated genes were identified to include regulators of mechanosensitive actin dynamics (*Myo1b, Casrp1, Pfn2*) and chromatin modifiers (*H2az1, Klf2, SMAD5*), while genes critical for heterochromatin maintenance (*Cbx7, Lemd2*) were downregulated. Moreover, transcriptomic comparison between JIB04 and DMSO‐treated stretched skin highlighted enrichment of DNA damage response pathways (Figure S32, Supporting Information), with significant upregulation of DNA repair and stress response genes.

These in vivo findings suggest that mechanosensitive pathways identified in vitro may also operate physiologically. Consistent reductions in H3K9me3 and increased perinuclear actin in stretched dermal regions indicate that nuclear mechanotransduction contributes to fibroblast adaptation and protection from mechanical DNA damage under normal loading. However, the surgical undermining model introduces additional complexity, including wound healing responses, immune infiltration, and heterogeneous cell populations, limiting direct comparison with in vitro conditions. Thus, while not definitive validation, these results support the physiological relevance of H3K9me3 regulation and perinuclear actin dynamics in nuclear mechanosensing, warranting further studies using advanced in vivo loading models to clarify their role in tissue homeostasis and disease.

## 3 Concluding Remarks

**Figure 10:**
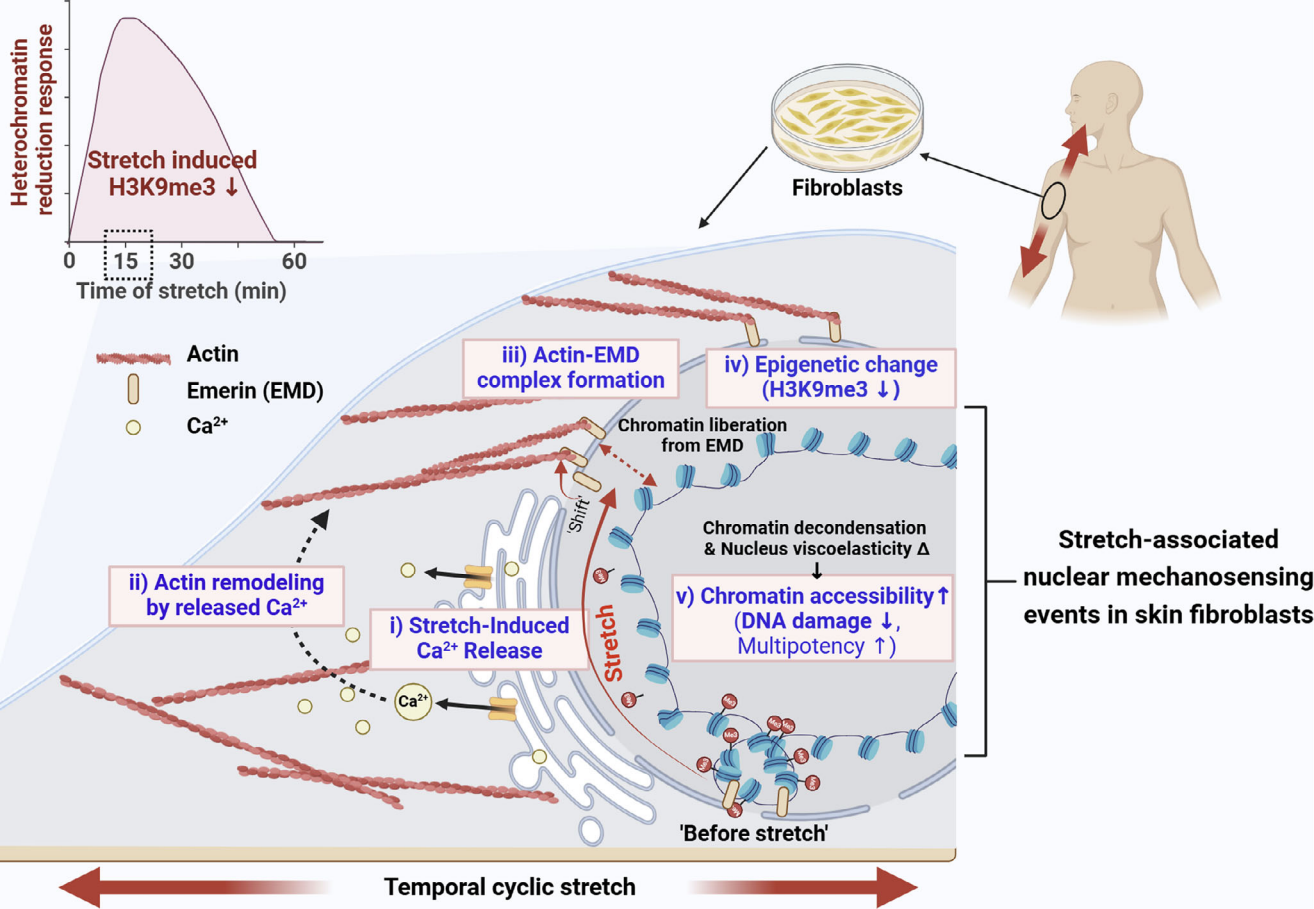
Summary of temporal stretch‐induced nuclear mechanosensing and chromatin accessibility mechanisms protecting against stress‐induced nuclear damage. Upon temporal stretching, fibroblasts exhibit a reduction in heterochromatin structure marked by H3K9me3 downregulation, which is mechanistically demonstrated by a series of nuclear mechanosensing events: i) intracellular Ca^2+^ release from the nucleus and ER, possibly due to elevated nuclear tension, which facilitates ii) increased F‐actin accumulation in the perinuclear region. iii) Perinuclear actin interacts with emerin, which translocates from the inner to the outer nuclear membrane, forming the F‐actin‐emerin complex. The response of these mechanosensitive molecules is accompanied by iv) epigenetic modifications characterized by liberation of heterochromatin (H3K9me3). These molecular and structural adaptations v) enhance the chromatin accessibility of genes associated with multipotency and DNA damage repair pathways, thereby offering fibroblasts enhanced plasticity as well as homeostasis to protect against stress‐induced nuclear damage.

In this study, we demonstrated a series of mechanosensing events within and around the nucleus in human dermal fibroblasts triggered by cyclic mechanical stretching (as summarized in **Figure** **10**). The events involve increased intracellular Ca^2+^ release from the nucleus and ER, perinuclear actin assembly, emerin translocation to the outer nuclear membrane, and downregulation of the heterochromatin marker H3K9me3. Through chromatin–gene interaction analysis, we identified increased accessibility of genes associated with mechanotransduction, chromatin remodeling, multipotency, and DNA damage repair. We further demonstrated that the protective effects against mechanical stress‐induced DNA damage were nullified when these mechanosensing pathways were inhibited, offering new insights into how mechanical forces can modulate epigenetic change and genomic stability in skin fibroblasts.

We further examined the potential physiological relevance of these nuclear mechanosensing events using a rodent model. Skin fibroblasts subjected to physiological stretching exhibited reduced H3K9me3 levels and increased perinuclear actin compared to those relieved of mechanical tension, suggesting that similar mechanotransduction pathways may operate in tissue contexts. While the tissue environment introduces complexity compared to controlled in vitro conditions, these findings suggest the potential significance of maintaining physiological mechanical stress in skin homeostasis and function. Understanding these mechanosensitive pathways may inform clinically recommended procedures, such as “layer‐by‐layer” suturing during surgery, which help preserve native mechanical tension and could potentially protect fibroblasts from mechanical stress‐induced genomic instability, ultimately contributing to improved wound healing outcomes.

This study sheds light on how fibroblasts dynamically regulate nuclear mechanics and epigenetic states to adapt to mechanical stress. Stretch‐induced chromatin remodeling may enhance cellular plasticity, priming fibroblasts for regenerative functions. This aligns with recent evidence showing that mechanical cues can enhance reprogramming efficiency by promoting nuclear deformation and chromatin accessibility.

In summary, this work reveals a mechanoprotective nuclear response to stretch involving coordinated cytoskeletal and epigenetic remodeling, offering insights into tissue homeostasis and potentially new strategies for regenerative medicine.

## 4 Experimental Section

### Cell Culture and Mechanical Stretch

Adult human dermal fibroblasts (aHDFs; PH10605A, Genlantis and GM00726, Coriell Institute for Medical Research) were cultured in high‐glucose Dulbecco's Modified Eagle Medium (DMEM; Welgene, Kyungbook, Republic of Korea) supplemented with 10% fetal bovine serum (FBS; 35‐015‐CV, Corning Incorporated, USA), 0.1 mm nonessential amino acids (MEM‐NEAA; 11 140 050, Gibco, USA), 1% penicillin‐streptomycin (15 140 122, Gibco, USA), and 2 mm GlutaMAX (35050‐061, Gibco, USA). For stretching experiments, polydimethylsiloxane (PDMS) chambers (SC‐0040, STREXCELL, USA) were washed and sterilized using UV treatment. The chambers were plasma‐treated for 15 min with a plasma processor (Vacuum Plasma System, FEMTOSCIENCE, Republic of Korea) to enhance collagen coating efficiency. A 50 µg mL^−1^ collagen type I solution was applied for 1 h at room temperature. After washing the coated chambers three times, they were seeded with aHDFs (passages 7–12) that were pre‐starved for 24 h in serum‐free medium to synchronize the cell cycle on conventional tissue culture plates (stiffness ≈2 GPa, SPL, Republic of Korea).

Stretching was performed 18 h after seeding the cells in the chambers. Cyclic uniaxial tension (4 s tension + 6 s rest) was applied at varying strain, frequency, and duration using an Automated Cell Stretching System (C‐pace EM, ionoptix, USA). When using inhibitors such as EGTA (2 mm, Sigma), blebbistatin (40 µm, Tocris, Bristol, UK), ML‐7 (25 µm, Tocris), Y‐27632 (200 µm, Tocris), cytochalasin D (100 nm, Tocris), CK666 (200 µm, Sigma), QC6352 (2 µm, Merck), and JIB 04 (400 nm, Tocris), each inhibitor was incubated for 1 h prior to and during the 15 min stretching period. No cell lines were used in this study, and experiments were conducted with cells from passages 5 to 11.

### Immunocytochemistry Analysis and Quantification

For cell fixation, chambers were washed with phosphate‐buffered saline (PBS; BPB‐9121‐004LR, T&I, Republic of Korea) or Hank's Balanced Salt Solution (HBSS; LB 003‐02, Welgene, with Ca^2+^ and Mg^2+^ to maintain the focal adhesion complex) at the appropriate time points. Cells were fixed with 4% paraformaldehyde solution (T&I) for 10 min at room temperature. Permeabilization was performed using either 0.2% Triton X‐100 (T8787‐100ML, Sigma Aldrich) or 0.003% digitonin (BN2006, Thermo Fisher) for 10 min. After washing three times with PBS, samples were blocked with 1% Bovine Serum Albumin (BSA; SM‐HEP‐250, Solmate) solution for 1 h at room temperature.^[^ ^73^, ^74^ ^]^

Primary antibodies were diluted in 1% BSA and incubated overnight at 4 °C. After washing three times, FITC or TRITC conjugated secondary antibodies (711‐095‐152, 715‐095‐150, 711‐025‐152, 715‐025‐150, Jackson ImmunoResearch, West Grove, USA) were incubated for 2 h at room temperature. Immunofluorescence images were captured using fluorescence microscopy (Olympus IX71, Olympus, Japan) or confocal microscopy (Nikon AX/AX R confocal, USA). For z‐stack imaging via confocal microscopy, constant slices with identical pinhole sizes were used. Images were acquired using consistent intensity, gain, and pinhole settings for accurate quantification.

Cell nuclei were stained with 4′,6‐diamidino‐2‐phenylindole dihydrochloride (DAPI, 1:100 000, D1306, Invitrogen), and F‐actin was stained with phalloidin (Alexa Fluor 546 Phalloidin, A22283, Invitrogen). The thickness of F‐actin fibers was quantified using the plot profile function of ImageJ (NIH, 1.52a) by measuring the peak width of the average value around the perinuclear region.

DAPI‐stained images were converted to rainbow channel images using NIS‐Elements Viewer (Nikon). Image parameters such as intensity and area were quantified using ImageJ or NIS‐Elements Viewer. The number of cells analyzed was represented as dots. Chromatin condensation parameter (CCP) values were calculated from DAPI images using the following equation:

(1) $$\mathrm{CCP}\;\left(\%\right)=\left(\mathrm{St}.\;\mathrm{Dev}/\mathrm{Mean}\;\mathrm{int}.\right)\times 100\left(\%\right)$$

### Western Blotting

Following stretching, cells were lysed with RIPA buffer (EBA‐1149, ElpisBio) supplemented with phosphatase and protease inhibitors (78 441, Thermo Fisher) on ice for 30 min. The lysates were then centrifuged at 12 000 g for 30 min at 4 °C. Total protein concentration in the cell lysates was determined using the Pierce BCA Protein Assay Kit (23 225, Thermo Fisher). Each sample (20 µg) was mixed with 5× SDS loading buffer and denatured at 95 °C for 5 min. The reduced samples were then loaded onto a 15% SDS‐PAGE gel, followed by transfer to nitrocellulose (NC) membranes (Bio‐Rad).

The membranes were blocked with 5% BSA in TBS‐T for 1 h at room temperature and subsequently incubated overnight at 4 °C with primary antibodies specific for H3K9me3 (1:1000, 1369S, Cell Signaling), H3K27me3 (1:1000, 9733S, Cell Signaling), H3ace (1:1000, 06‐599, Sigma‐Aldrich), and GAPDH (1:1000, SC‐47724, Santa Cruz) in blocking buffer. After washing with TBS‐T, the membranes were incubated with secondary HRP‐linked antibodies (anti‐mouse or anti‐rabbit, Cell Signaling) at a dilution of 1:10 000 for 1 h at room temperature. Protein signals were visualized using SuperSignal West Pico PLUS Chemiluminescent Substrate (34 579, Thermo Fisher Scientific) and captured using the iBright 1500 imaging system (Thermo Fisher Scientific).

### Quantitative Polymerase Chain Reaction (qPCR)

After stretching, total RNA was isolated using Ribospin (GeneAll, Seoul, Korea). RNA quality was assessed with an Agilent 2100 Bioanalyzer (Agilent Technologies, Amstelveen, The Netherlands), and RNA quantification was performed using an ND‐2000 Spectrophotometer (Thermo Inc., DE, USA). A total of 2 µg of RNA was reverse transcribed into cDNA using a 2720 Thermal Cycler (Applied Biosystems) with a pre‐mixture (AccuPower RT PreMix, Bioneer, Korea), oligo‐dT (Qiagen, Venlo, Netherlands), and Random Hexamers (Qiagen, Venlo, Netherlands).^[^ ^75^ ^]^

Quantitative mRNA expression levels (*n* = 3) were measured using a real‐time PCR machine (StepOnePlus, Applied Biosystems) with SYBR Green (Applied Biosystems), following the manufacturer's instructions. After confirming qPCR efficiency, mRNA expression levels in each group were calculated as relative fold change over the control, normalized to GAPDH, using the 2*^−ΔΔCt^* method from StepOne software v2.3. Data are presented as means ± SD from independent triplicate experiments. *Ct*‐values were extracted from the linear range of amplification.

### Plasmid Transfection and Live Imaging

For live‐cell imaging of lamina‐associated heterochromatin during stretch experiments, cells were transfected with the DD‐DamWT‐LMNB1‐IRES2‐mCherry plasmid (Dam‐LMNB1; addgene, #159601) and m^6^A‐Tracer‐NES plasmid (m^6^A‐Tracer; addgene, #159 607) using the Xfect Transfection Reagent (Clontech, PT5003‐2).^[^ ^41^ ^]^ Plasmid amplification followed an established protocol.^[^ ^76^ ^]^ Briefly, bacteria were streaked onto an LB plate containing the appropriate antibiotic. DNA plasmids were amplified and isolated using Plasmid Midi‐Prep (Qiagen, 12 145) from cultured bacteria grown in Luria‐Bertani (LB) medium. Cells were transfected 18 h after seeding in the chambers according to the manufacturer's instructions. Specifically, 5 µg of plasmid DNA was mixed with the Xfect polymer and Xfect reaction buffer, followed by a 15‐min incubation at room temperature. This mixture was added to the cell growth medium and incubated at 37 °C for 4 h, after which the cells were washed and the medium was replaced. Live imaging was performed 24 h post‐transfection. Stretch live imaging was conducted using a Microscope Mountable Uniaxial Cell Stretching System (STREXCELL, ST‐1500, USA) connected to a confocal microscope (Nikon AX/AX R confocal, USA).

### RNA Interference Study

Small interfering RNAs (siRNAs) targeting emerin, Xpo6, and LMNA, as well as a negative control siRNA (SN‐1003), were obtained from Bioneer (Republic of Korea). Transfections were performed using Lipofector‐EZ (AB‐LF‐ez150, Aptabio, Republic of Korea) following the manufacturer's instructions. After 4 h of transfection, the medium was replaced, and the cells were incubated for an additional 24 h.

### Ca^2+^ Live Imaging

For live imaging of intracellular Ca^2+^, Cal‐520 AM (5 µm, ab171868, Abcam) was applied to the cells for 1 h at 37 °C, followed by washing twice with HBSS. Imaging was performed in fresh medium containing 2% FBS and phenol red‐free DMEM. Cal‐520 AM staining was carried out 24 h after the cells were placed in the stretch chamber. Live images were captured using a confocal microscope (Nikon AX/AX R confocal, USA) for 5 min before and after stretching.

Calcium inhibitors were administered 1 h before imaging. The inhibitors used were BAPTA‐AM (10 µm, 2787, Tocris), BAPTA (2 mm, 2786, Tocris), gadolinium chloride (25 µm, 4741, Tocris), and 2APB (100 µm, 1224, Tocris).

### DNA Oligonucleotide Fluorescence In Situ Hybridization (FISH)

Following washing three times with HBSS, the stretch chamber was fixed using a 4% paraformaldehyde solution for 10 min at room temperature. Subsequently, the samples were permeabilized with 0.5% Triton X‐100 (Cayman Chemical, 600 217) for 15 min at room temperature, followed by another washing three times. Treatment with 0.1 m hydrochloric acid (Sigma, H9892) solution for 5 min at room temperature and subsequent triple washing ensued. A solution of 10 µg mL^−1^ RNase A (Thermo Fisher, EN0531) was applied, followed by incubation at 37 °C for 2 h. Following this, samples were washed with 2× Saline Sodium Citrate (SSC; Thermo Fisher, AM9763) and incubated with hybridization buffer (2× SSC with 0.1% Tween (Sigma, P‐7949), 50% formamide (Invitrogen, 15 515 026), 10% dextran sulfate (Merck Millipore, 3730) for 1 h at 47 °C. Introduction of 25 µg mL^−1^ DNA primary oligo probe to the hybridization buffer ensued, with samples treated at 90 °C for 3 min, followed by incubation for 16 h at 47 °C. Thereafter, samples were washed twice with 2× SSC at 47 °C for 10 min. Finally, DAPI staining was conducted for 10 min at room temperature, and imaging was performed using confocal microscopy.

### Fluorescence Resonance Energy Transfer (FRET) Study

To assess cell membrane tension, the plasmid pcDNA Nesprin tension sensor (TS, Addgene, #68127) was employed, harboring TFP, an elastin linker, and Venus. The midi prep procedure for transfection was detailed in the preceding section “Plasmid transfection and live imaging”. Transfections were carried out 18 h post‐seeding of cells onto stretch chambers, utilizing Xfect Transfection Reagent (Clontech, PT5003‐2) as per the manufacturer's instructions. Following transfection, cells were allowed to incubate for 24 h before fixation during stretching. Confocal microscopy (Nikon AX/AX R confocal, USA) equipped with a ×60 objective facilitated image capture. Laser excitation at 458 nm and emission from mTFP (494 nm) and yellow (527 nm) were employed for image acquisition. FRET analysis was conducted using the FRET Analyzer plugin within Fiji ImageJ (2.3/1.53q), employing a customized methodology.^[^ ^77^, ^78^, ^79^ ^]^

### Atomic Force Microscopy (AFM)

To quantify nuclear stiffness, cells were seeded within a stretch chamber and allowed to incubate for 18 h prior to stretching. Subsequently, nuclear stiffness measurements were precisely assessed using cell nuclei mounted onto an atomic force microscope (PAVONE, OPTICS11life, USA) immediately following stretching, employing irradiation through a cantilever tip. Targeting of the cell nucleus was facilitated by a ×20 phase contrast objective integrated with the AFM setup. The stiffness of the probe was calibrated to 0.470 k (N m^−1^), and a tip radius of 10.5 µm was utilized for the measurements.

### Transmission Electron Microscopy (TEM)

Specimens were fixed for 12 h in a solution comprising 2% glutaraldehyde and 2% paraformaldehyde in 0.1 m phosphate buffer (pH 7.4), followed by washing in 0.1 m phosphate buffer. Post‐fixation was carried out using 1% osmium tetroxide (OsO_4_) in 0.1 m phosphate buffer for 2 h. Subsequent steps involved dehydration with an ascending ethanol series (50, 60, 70, 80, 90, 95, 100, and 100%) for 10 min each, followed by infiltration with propylene oxide for 10 min. Specimens were embedded utilizing a Poly/Bed 812 kit (Polysciences), polymerized in an electron microscope oven (TD‐700, DOSAKA, Japan) at 65 °C for 12 h. The resulting block was sectioned using a diamond knife in the Ultra‐microtome (UC7, Leica Microsystems Ltd, Austria) into 200 nm semi‐thin sections, which were stained with toluidine blue for observation under an optical microscope. Subsequently, the region of interest was further sectioned into 80 nm thin sections using the ultra‐microtome, placed onto copper grids, and double‐stained with 5% uranyl acetate for 20 min and 3% lead citrate for 7 min. Imaging was performed using a transmission electron microscope (HT7800, HITACHI, Tokyo, Japan) operated at an acceleration voltage of 90 kV, equipped with an RC camera.

### Assay for Transposase‐Accessible Chromatin with Sequencing (ATAC‐Seq)

For the ATAC‐seq experiment, cells were seeded into the stretch chamber and allowed to incubate for 18 h. Subsequently, following a 15‐min stretching period, cells were washed twice with ice‐cold HBSS. The stretched and non‐stretched groups were then lysed and centrifuged at 500 g for 10 min at 4 °C. Following supernatant removal, the transposition reaction mix (FC‐121‐1030, Illumina) was added and incubated at 37 °C for 30 min. Post‐transposition, DNA fragments were purified using a Qiagen MinElute PCR purification kit according to the manufacturer's instructions.

Subsequent procedures were performed at Magrogen (South Korea). Briefly, transposed DNA fragments underwent amplification using the Nextera DNA Flex kit. To mitigate potential biases related to GC content and fragment size during PCR amplification, the optimal number of cycles was determined using qPCR. An additional cycle determination was conducted via qPCR side reaction, identifying the cycle number corresponding to 1/4 of the maximum fluorescent intensity by plotting linear Rn against the cycle. The remaining PCR reaction was then performed using the determined cycle number from qPCR. Following amplification, libraries were purified and quantified according to the qPCR Quantification Protocol Guide (KAPA) and evaluated for quality using Bioanalyzer (Agilent Technologies). Subsequently, the libraries underwent sequencing on the HiSeq platform (Illumina). Raw data obtained from ATAC sequencing were analyzed using ExPADA software (Ebiogen Inc., Republic of Korea) to identify regions of significant accessibility.

### Cell Differentiation Study

Cells were seeded into stretch chambers and incubated in growth medium for 18 h. Following a 15‐min stretching period, the growth medium was immediately replaced with differentiation medium. This process was repeated for three consecutive days. qRT‐PCR was then employed to measure gene expression. The compositions of the differentiation media were as follows. Neurogenic differentiation medium: Neurobasal Plus media (Gibco, A3582901), 2% B27 (Gibco, A3582801), 2 mm L‐GlutaMAX, 2% Stempro Neural Supplement (Gibco, 15 140 122), 20 ng mL^−1^ epidermal growth factor (Peprotech, AF‐100‐15), 20 ng mL^−1^ basic fibroblast growth factor (Peprotech, 100‐18B), and 1% PS. Myogenic differentiation medium: DMEM/F‐12 (Thermo Fisher, 11 320 033), 1 ng mL^−1^ TGF‐β1 (Peprotech, 100‐21C), 0.1 mm nonessential amino acids (Gibco, 11 140 050), 1% Insulin‐Transferrin‐Selenium (Thermo Fisher, 41 400 045). Osteogenic differentiation medium: high glucose DMEM, 10% FBS, 1% P/S, 10 mm β‐glycerophosphate, 50 µg mL^−1^ ascorbic acid, 10 nm dexamethasone, and 10 µm phenamil.

### Animal Study

In vivo experiments were conducted using mice. All surgical procedures adhered to the guidelines approved by the Institutional Animal Care and Use Committee at Dankook University, Republic of Korea (approval no. DKU‐24‐047). Healthy male C57BL/6 wild‐type mice, aged nine weeks, were housed in a specific pathogen‐free facility with controlled atmospheric and humidity conditions. They had ad libitum access to water and food and were exposed to a light/dark cycle, receiving appropriate care according to the animal care protocol. Mice were anesthetized by intraperitoneal (IP) injection using 10 mg kg^−1^ of Rompun and 80 mg kg^−1^ of Ketamine and cleaned for surgery before according to the protocol.^[^ ^80^, ^81^ ^]^ After a midline incision of the dorsal skin, surgical scissors were used to dissect the subcutaneous tissue and muscle layers horizontally on one side (*n* = 3). Following closure with surgical sutures, the animals were euthanized after a 24‐hour period. For the inhibition assay, an incision was made in the middle of the dorsal skin, followed by closure with surgical sutures. After 24 hours, DMSO was injected on one side and the inhibitor (20 mg kg^−1^ of JIB‐04 or 10 mg kg^−1^ of QC) on the other side (*n* = 3). The animals were euthanized 24 hours after injection. Tissue samples were collected and immediately fixed in 10% neutral buffered formalin under normal conditions. For further analysis, samples were embedded in paraffin, sectioned, and stained using standard histological protocols, including H&E and immunofluorescence staining.

For spatial transcriptomics analysis, tissue sections were processed using the GeoMx Digital Spatial Profiling platform (NanoString Technologies) according to the manufacturer's protocol (*n* = 3).^[^ ^82^, ^83^ ^]^ Sections were stained with a multiplexed antibody panel including α‐SMA‐FITC (Thermo, #53‐9760‐82, FITC/525 nm) for myofibroblasts and myocytes exclusion by strong intensity, Syto83 for DNA visualization (Thermo, S11364, Cy3/568 nm), CD11b‐Texas Red (BioLegend, #101 254, Texas Red/615 nm) for immune cell exclusion, and H3K9me3‐Cy5 (Abcam, ab288339, Cy5/666 nm) for heterochromatin state assessment. Regions of interest were selected based on cell‐type‐specific markers, specifically targeting dermal fibroblasts while excluding CD11b+ immune cells and high α‐SMA+ myofibroblasts/myocytes. Digital counting of RNA transcripts was performed using the GeoMx DSP instrument, and data were analyzed using the GeoMx NGS Pipeline and GeoMx Data Analysis Suite.^[^ ^84^ ^]^

### Statistical Analysis

Statistical analyses were conducted using GraphPad Prism 8.3.0 software (GraphPad Software, San Diego, USA). Data distribution was visualized as box‐and‐whisker plots (minimum to maximum) with all data points shown. All experiments were performed with at least three independent biological replicates. Comparisons between two groups were analyzed using an unpaired two‐tailed Student's *t*‐test. For multiple groups were analyzed using one‐way ANOVA followed by Dunnett's or Tukey's post hoc tests, as specified in the figure legends. For quantitative PCR data presented in the Supporting information, results are shown as mean ± standard deviation (SD). Statistical significance was defined as *p* < 0.05.

### Information Summary

Details of antibodies, primer sequences, DNA probe sequences, chemicals, and analysis time points under stretching conditions are provided in Tables S1–S5 (Supporting Information), respectively.

## Conflict of Interest

The authors declare no conflict of interest.

## Author Contributions

H.‐W.S. and J.‐Y.Y. contributed equally to this work as co‐first authors. H.‐W.S., J.‐Y.Y, J.‐H.L., and H.‐W.K. conceptualized the study and designed the experiments. J.‐H.L. and H.‐W.K. supervised the study. H.‐W.S., J.‐Y.Y., S.K., C.J.L., S.‐M.P., Y.S., S.H., and D.‐J.L. performed characterized the samples and analyzed the data. H.‐W.S., J.‐Y.Y., S.K., C.J.L., and D.‐J.L. performed the in vitro cell and in vivo studies, and H.L. performed modeling analysis. H.‐W.S. and J.‐Y.Y. drafted the manuscript. K.W.L. commented and edited the manuscript. J.‐H.L. and H.‐W.K. wrote, edited, and approved the manuscript.

## Supporting information



Supporting Information

## Data Availability

The data that support the findings of this study are available from the corresponding author upon reasonable request.

## References

[1] T. Panciera , L. Azzolin , M. Cordenonsi , S. Piccolo , Nat. Rev. Mol. Cell Biol. 2017, 18, 758. 28951564 10.1038/nrm.2017.87 PMC6192510

[2] K. H. Vining , D. J. Mooney , Nat. Rev. Mol. Cell Biol. 2017, 18, 728. 29115301 10.1038/nrm.2017.108 PMC5803560

[3] S. Chanet , A. C. Martin , Prog. Mol. Biol. Transl. Sci. 2014, 126, 317. 25081624 10.1016/B978-0-12-394624-9.00013-0 PMC4412274

[4] S. Dupont , S. A. Wickstrom , Nat. Rev. Genet. 2022, 23, 624. 35606569 10.1038/s41576-022-00493-6

[5] L. Li , J. Eyckmans , C. S. Chen , Nat. Mater. 2017, 16, 1164. 29170549 10.1038/nmat5049 PMC7001850

[6] B. Ladoux , R. M. Mege , Nat. Rev. Mol. Cell Biol. 2017, 18, 743. 29115298 10.1038/nrm.2017.98

[7] R. C. Ransom , A. C. Carter , A. Salhotra , T. Leavitt , O. Marecic , M. P. Murphy , M. L. Lopez , Y. Wei , C. D. Marshall , E. Z. Shen , R. E. Jones , A. Sharir , O. D. Klein , C. K. F. Chan , D. C. Wan , H. Y. Chang , M. T. Longaker , Nature 2018, 563, 514. 30356216 10.1038/s41586-018-0650-9 PMC6481292

[8] J. S. Silver , K. A. Günay , A. A. Cutler , T. O. Vogler , T. E. Brown , B. T. Pawlikowski , O. J. Bednarski , K. L. Bannister , C. J. Rogowski , A. G. Mckay , F. W. DelRio , B. B. Olwin , K. S. Anseth , Sci. Adv. 2021, 7, abe4501. 10.1126/sciadv.abe4501 PMC7954458 33712460

[9] C. J. Walker , C. Crocini , D. Ramirez , A. R. Killaars , J. C. Grim , B. A. Aguado , K. Clark , M. A. Allen , R. D. Dowell , L. A. Leinwand , K. S. Anseth , Nat. Biomed. Eng. 2021, 5, 1485. 33875841 10.1038/s41551-021-00709-w PMC9102466

[10] S. M. Schreiner , P. K. Koo , Y. Zhao , S. G. Mochrie , M. C. King , Nat. Commun. 2015, 6, 7159. 26074052 10.1038/ncomms8159 PMC4490570

[11] S. Jiao , C. Li , F. Guo , J. Zhang , H. Zhang , Z. Cao , W. Wang , W. Bu , M. Lin , J. Lü , Z. Zhou , Nat. Commun. 2023, 14, 6416. 37828059 10.1038/s41467-023-42187-5 PMC10570371

[12] C. R. Hsia , J. McAllister , O. Hasan , J. Judd , S. Lee , R. Agrawal , C.‐Y. Chang , P. Soloway , J. Lammerding , iScience 2022, 25, 104978. 36117991 10.1016/j.isci.2022.104978 PMC9474860

[13] A. J. Engler , S. Sen , H. L. Sweeney , D. E. Discher , Cell 2006, 126, 677. 16923388 10.1016/j.cell.2006.06.044

[14] P. Romani , L. Valcarcel‐Jimenez , C. Frezza , S. Dupont , Nat. Rev. Mol. Cell Biol. 2021, 22, 22. 33188273 10.1038/s41580-020-00306-w

[15] S.‐M. Park , J.‐H. Lee , K. S. Ahn , H. W. Shim , J.‐Y. Yoon , J. Hyun , J. H. Lee , S. Jang , K. H. Yoo , Y.‐K. Jang , T.‐J. Kim , H. K. Kim , M. R. Lee , J.‐H. Jang , H. Shim , H.‐W. Kim , Adv. Sci. 2023, 10, 2303395. 10.1002/advs.202303395 PMC10646259 37727069

[16] H. Q. Le , S. Ghatak , C.‐Y. C. Yeung , F. Tellkamp , C. Günschmann , C. Dieterich , A. Yeroslaviz , B. Habermann , A. Pombo , C. M. Niessen , S. A. Wickström , Nat. Cell Biol. 2016, 18, 864. 27398909 10.1038/ncb3387

[17] C. Yang , F. W. DelRio , H. Ma , A. R. Killaars , L. P. Basta , K. A. Kyburz , K. S. Anseth , Proc. Natl. Acad. Sci. U. S. A. 2016, 113, E4439. 27436901 10.1073/pnas.1609731113 PMC4978284

[18] K. Damodaran , S. Venkatachalapathy , F. Alisafaei , A. V. Radhakrishnan , D. Sharma Jokhun , V. B. Shenoy , G. V. Shivashankar , Mol. Biol. Cell 2018, 29, 3039. 30256731 10.1091/mbc.E18-04-0256 PMC6333178

[19] S.‐J. Heo , S. Thakur , X. Chen , C. Loebel , B. Xia , R. McBeath , J. A. Burdick , V. B. Shenoy , R. L. Mauck , M. Lakadamyali , Nat. Biomed. Eng. 2023, 7, 177. 35996026 10.1038/s41551-022-00910-5 PMC10053755

[20] Y. Song , J. Soto , B. Chen , T. Hoffman , W. Zhao , N. Zhu , Q. Peng , L. Liu , C. Ly , P. K. Wong , Y. Wang , A. C. Rowat , S. K. Kurdistani , S. Li , Nat. Mater. 2022, 21, 1191. 35927431 10.1038/s41563-022-01312-3 PMC9529815

[21] N. Jain , J. M. Lord , V. V. Mechanoimmunology , APL Bioeng. 2022, 6, 031502. 36051106 10.1063/5.0087699 PMC9427154

[22] S. W. Crowder , V. Leonardo , T. Whittaker , P. Papathanasiou , M. M. Stevens , Cell Stem Cell 2016, 18, 39. 26748755 10.1016/j.stem.2015.12.012 PMC5409508

[23] A. A. Anlas , C. M. Nelson , Curr. Opin. Cell Biol. 2018, 54, 98. 29890398 10.1016/j.ceb.2018.05.012 PMC6214752

[24] L. Huang , L. Wenzhi , Am. Surg. 2014, 80, 587. 24887797

[25] X. Fu , A. Taghizadeh , M. Taghizadeh , C. J. Li , N. K. Lim , J.‐H. Lee , H. S. Kim , H.‐W. Kim , Adv. Sci. 2024, 11, 2308253. 10.1002/advs.202308253 PMC11022731 38353381

[26] M. Topaz , N. N. Carmel , G. Topaz , M. Li , Y. Z. Li , Medicine 2014, 93, 234. 10.1097/MD.0000000000000234 PMC4603089 25526444

[27] L. Casares , R. Vincent , D. Zalvidea , N. Campillo , D. Navajas , M. Arroyo , X. Trepat , Nat. Mater. 2015, 14, 343. 25664452 10.1038/nmat4206 PMC4374166

[28] M. M. Nava , Y. A. Miroshnikova , L. C. Biggs , D. B. Whitefield , F. Metge , J. Boucas , H. Vihinen , E. Jokitalo , X. Li , J. M. García Arcos , B. Hoffmann , R. Merkel , C. M. Niessen , K. N. Dahl , S. A. Wickström , Cell 2020, 181, 800. 32302590 10.1016/j.cell.2020.03.052 PMC7237863

[29] A. Livne , E. Bouchbinder , B. Geiger , Nat. Commun. 2014, 5, 3938. 24875391 10.1038/ncomms4938 PMC4066201

[30] K. Chen , A. Vigliotti , M. Bacca , R. M. McMeeking , V. S. Deshpande , J. W. Holmes , Proc. Natl. Acad. Sci. U. S. A. 2018, 115, 986. 29343646 10.1073/pnas.1715059115 PMC5798351

[31] C. R. Hsia , D. P. Melters , Y. Dalal , J. Mol. Biol. 2023, 435, 168019. 37330288 10.1016/j.jmb.2023.168019 PMC10567996

[32] D. Nicetto , K. S. Zaret , Curr. Opin. Genet. Dev. 2019, 55, 1. 31103921 10.1016/j.gde.2019.04.013 PMC6759373

[33] M. Ninova , K. Fejes Toth , A. A. Aravin , Development 2019, 146, dev181180. 31540910 10.1242/dev.181180 PMC6803365

[34] W. Liu , A. Padhi , X. Zhang , J. Narendran , M. A. Anastasio , A. S. Nain , J. Irudayaraj , ACS Nano 2022, 16, 10754. 35803582 10.1021/acsnano.2c02660 PMC9332347

[35] F. Rashid , W. Liu , Q. Wang , B. Ji , J. Irudayaraj , N. Wang , Proc. Natl. Acad. Sci. U. S. A. 2023, 120, 2221432120. 10.1073/pnas.2221432120 PMC10068814 36943889

[36] A. Tajik , Y. Zhang , F. Wei , J. Sun , Q. Jia , W. Zhou , R. Singh , N. Khanna , A. S. Belmont , N. Wang , Nat. Mater. 2016, 15, 1287. 27548707 10.1038/nmat4729 PMC5121013

[37] F. Alisafaei , D. S. Jokhun , G. V. Shivashankar , V. B. Shenoy , Proc. Natl. Acad. Sci. U. S. A. 2019, 116, 13200. 31209017 10.1073/pnas.1902035116 PMC6613080

[38] B. Sen , C. Guilluy , Z. Xie , N. Case , M. Styner , J. Thomas , I. Oguz , C. Rubin , K. Burridge , J. Rubin , Stem Cells 2011, 29, 1829. 21898699 10.1002/stem.732 PMC3588570

[39] M. Borsos , S. M. Perricone , T. Schauer , J. Pontabry , K. L. de Luca , S. S. de Vries , E. R. Ruiz‐Morales , M.‐E. Torres‐Padilla , J. Kind , Nature 2019, 569, 729. 31118510 10.1038/s41586-019-1233-0 PMC6546605

[40] N. Briand , P. Collas , Genome Biol. 2020, 21, 85. 32241294 10.1186/s13059-020-02003-5 PMC7114793

[41] N. Altemose , A. Maslan , C. Rios‐Martinez , A. Lai , J. A. White , S. A. µDamID , Cell Syst. 2020, 11, 354. 33099405 10.1016/j.cels.2020.08.015 PMC7588622

[42] S. J. Shin , B. Bayarkhangai , K. Tsogtbaatar , M. Yuxuan , S.‐H. Kim , Y.‐J. Kim , A. Taghizadeh , D. Kim , D.‐H. Kim , J.‐H. Lee , J. Hyun , H.‐W. Kim , Adv. Sci. 2025, 12, 2403409. 10.1002/advs.202403409 PMC11848612 39828979

[43] B. van Steensel , A. S. Belmont , Cell 2017, 169, 780. 28525751 10.1016/j.cell.2017.04.022 PMC5532494

[44] A. Kumar , M. Mazzanti , M. Mistrik , M. Kosar , G. V. Beznoussenko , A. A. Mironov , M. Garrè , D. Parazzoli , G. V. Shivashankar , G. Scita , J. Bartek , M. Foiani , Cell 2014, 158, 633. 25083873 10.1016/j.cell.2014.05.046 PMC4121522

[45] W. Tang , X. Chen , X. Wang , M. Zhu , G. Shan , T. Wang , W. Dou , J. Wang , J. Law , Z. Gong , S. Hopyan , X. Huang , Y. Sun , Proc. Natl. Acad. Sci. U. S. A. 2023, 120, 2307356120. 10.1073/pnas.2307356120 PMC10483616 37639585

[46] Y. A. Miroshnikova , S. A. Wickström , Cold Spring Harbor Perspect. Biol. 2022, 14, a039685. 10.1101/cshperspect.a039685 PMC8725626 34187806

[47] S. B. Khatau , C. M. Hale , P. J. Stewart‐Hutchinson , M. S. Patel , C. L. Stewart , P. C. Searson , D. Hodzic , D. Wirtz , Proc. Natl. Acad. Sci. U. S. A. 2009, 106, 19017. 19850871 10.1073/pnas.0908686106 PMC2776434

[48] M. Maninova , J. Caslavsky , T. Vomastek , Protoplasma 2017, 254, 1207. 28101692 10.1007/s00709-017-1077-0

[49] J. K. Kim , A. Louhghalam , G. Lee , B. W. Schafer , D. Wirtz , D. H. Kim , Nat. Commun. 2017, 8, 2123. 29242553 10.1038/s41467-017-02217-5 PMC5730574

[50] H.‐R. Thiam , P. Vargas , N. Carpi , C. L. Crespo , M. Raab , E. Terriac , M. C. King , J. Jacobelli , A. S. Alberts , T. Stradal , A.‐M. Lennon‐Dumenil , M. Piel , Nat. Commun. 2016, 7, 10997. 26975831 10.1038/ncomms10997 PMC4796365

[51] T. Stuven , E. Hartmann , D. Gorlich , EMBO J. 2003, 22, 5928. 14592989 10.1093/emboj/cdg565 PMC275422

[52] Y. Kalukula , A. D. Stephens , J. Lammerding , S. Gabriele , Nat. Rev. Mol. Cell Biol. 2022, 23, 583. 35513718 10.1038/s41580-022-00480-z PMC9902167

[53] Y. A. Miroshnikova , S. Manet , X. Li , S. A. Wickstrom , E. Faurobert , Mol. Biol. Cell 2021, 32, 1724. 34081532 10.1091/mbc.E21-03-0106 PMC8684738

[54] F. Lehne , S. Bogdan , Front. Cell Dev. Biol. 2023, 11, 1171930. 37025173 10.3389/fcell.2023.1171930 PMC10070769

[55] R. J. Gasperini , M. Pavez , A. C. Thompson , C. B. Mitchell , H. Hardy , K. M. Young , J. K. Chilton , L. Foa , Mol. Cell. Neurosci. 2017, 84, 29. 28765051 10.1016/j.mcn.2017.07.006

[56] X. Li , Y. Cheng , Z. Wang , J. Zhou , Y. Jia , X. He , L. Zhao , Y. Dong , Y. Fan , X. Yang , B. Shen , X. Wu , J. Wang , C. Xiong , L. Wei , X. Li , J. Wang , Cell Death Dis. 2020, 11, 1009. 33230171 10.1038/s41419-020-03181-7 PMC7683721

[57] D. Ranade , R. Pradhan , M. Jayakrishnan , S. Hegde , K. Sengupta , BMC Mol. Cell Biol. 2019, 20, 11. 31117946 10.1186/s12860-019-0192-5 PMC6532135

[58] P. Nastały , D. Purushothaman , S. Marchesi , A. Poli , T. Lendenmann , G. R. Kidiyoor , G. V. Beznoussenko , S. Lavore , O. M. Romano , D. Poulikakos , M. C. Lagomarsino , A. A. Mironov , A. Ferrari , P. Maiuri , Nat. Commun. 2020, 11, 2122. 32358486 10.1038/s41467-020-15910-9 PMC7195445

[59] J. D. Buenrostro , P. G. Giresi , L. C. Zaba , H. Y. Chang , W. J. Greenleaf , Nat. Methods 2013, 10, 1213. 24097267 10.1038/nmeth.2688 PMC3959825

[60] F. Yan , D. R. Powell , D. J. Curtis , N. C. Wong , Genome Biol. 2020, 21, 22. 32014034 10.1186/s13059-020-1929-3 PMC6996192

[61] W. da Huang , B. T. Sherman , R. A. Lempicki , Nat. Protoc. 2009, 4, 44. 19131956 10.1038/nprot.2008.211

[62] M. Kinisu , Y. J. Choi , C. Cattoglio , K. Liu , H. Roux de Bezieux , R. Valbuena , N. Pum , S. Dudoit , H. Huang , Z. Xuan , S. Y. Kim , L. He , Cell Rep. 2021, 37, 109982. 34758315 10.1016/j.celrep.2021.109982 PMC8711565

[63] E. E. Kim , A. Shekhar , J. Ramachandran , A. Khodadadi‐Jamayran , F.‐Y. Liu , J. Zhang , G. I. Fishman , Development 2023, 150, dev202054. 37787076 10.1242/dev.202054 PMC10652039

[64] K. Shiraishi , P. P. Shah , M. P. Morley , C. Loebel , G. T. Santini , J. Katzen , M. C. Basil , S. M. Lin , J. D. Planer , E. Cantu , D. L. Jones , A. N. Nottingham , S. Li , F. L. Cardenas‐Diaz , S. Zhou , J. A. Burdick , R. Jain , E. E. Morrisey , Cell 2023, 186, 1478. 36870331 10.1016/j.cell.2023.02.010 PMC10065960

[65] X. Jia , W. Lin , W. Wang , Cell Regener. 2023, 12, 19. 10.1186/s13619-023-00162-x PMC10232395 37259007

[66] J. S. Becker , D. Nicetto , K. S. Zaret , Trends Genet. 2016, 32, 29. 26675384 10.1016/j.tig.2015.11.001 PMC4698194

[67] D. Indana , A. Zakharov , Y. Lim , A. R. Dunn , N. Bhutani , V. B. Shenoy , O. Chaudhuri , Cell Stem Cell 2024, 31, 640. 38701758 10.1016/j.stem.2024.03.016 PMC11323070

[68] M. Versaevel , T. Grevesse , S. Gabriele , Nat. Commun. 2012, 3, 671. 22334074 10.1038/ncomms1668

[69] A. J. Lomakin , C. J. Cattin , D. Cuvelier , Z. Alraies , M. Molina , G. P. F. Nader , N. Srivastava , P. J. Sáez , J. M. Garcia‐Arcos , I. Y. Zhitnyak , A. Bhargava , M. K. Driscoll , E. S. Welf , R. Fiolka , R. J. Petrie , N. S. De Silva , J. M. González‐Granado , N. Manel , A. M. Lennon‐Duménil , D. J. Müller , M. Piel , Science 2020, 370, aba2894. 10.1126/science.aba2894 PMC8059074 33060332

[70] V. Venturini , F. Pezzano , F. Català Castro , H.‐M. Häkkinen , S. Jiménez‐Delgado , M. Colomer‐Rosell , M. Marro , Q. Tolosa‐Ramon , S. Paz‐López , M. A. Valverde , J. Weghuber , P. Loza‐Alvarez , M. Krieg , S. Wieser , V. Ruprecht , Science 2020, 370, aba2644. 10.1126/science.aba2644 33060331

[71] O. Wintner , N. Hirsch‐Attas , M. Schlossberg , F. Brofman , R. Friedman , M. Kupervaser , D. Kitsberg , A. Buxboim , Adv. Sci. 2020, 7, 1901222. 10.1002/advs.201901222 PMC7175345 32328409

[72] J. Joo , A. Pourang , C. N. Tchanque‐Fossuo , A. W. Armstrong , D. M. Tartar , T. H. King , R. K. Sivamani , D. B. Eisen , Arch. Dermatol. Res. 2022, 314, 697. 34546436 10.1007/s00403-021-02280-5 PMC9307554

[73] M.‐J. Kim , H.‐J. Ahn , D. Kong , S. Lee , D.‐H. Kim , K.‐S. Kang , J. Tissue Eng. 2024, 15, 20417314241248753. 38725732 10.1177/20417314241248753 PMC11080775

[74] W. Wan , X. Wang , R. Zhang , Y. Li , H. Wu , Y. Liu , F. Zhang , J. Liu , G. Liu , L. Zhou , Z. Wu , H. Mao , J. Yang , J. Tissue Eng. 2025, 16, 20417314251328128. 40171244 10.1177/20417314251328128 PMC11960185

[75] D.‐Y. Li , Y.‐M. Li , D.‐Y. Lv , T. Deng , X. Zeng , L. You , Q.‐Y. Pang , Y. Li , B.‐M. Zhu , J. Tissue Eng. 2024, 15, 20417314241268917. 39329066 10.1177/20417314241268917 PMC11425747

[76] W. Kang , D. S. Lee , J. H. Jang , PLoS One 2015, 10, 0123402. 10.1371/journal.pone.0123402 PMC4406711 25901352

[77] P. T. Arsenovic , I. Ramachandran , K. Bathula , R. Zhu , J. D. Narang , N. A. Noll , C. A. Lemmon , G. G. Gundersen , D. E. Conway , Biophys. J. 2016, 110, 34. 26745407 10.1016/j.bpj.2015.11.014 PMC4805861

[78] J. S. Suh , H. S. Kim , T. J. Kim , Sens. Actuators, B 2021, 334, 129663. 10.1016/j.snb.2021.129663 PMC7885701 33612970

[79] B. N. Moeun , F. Lemaire , A. M. Smink , H. Ebrahimi Orimi , R. L. Leask , P. de Vos , C. A. Hoesli , J. Tissue Eng. 2025, 16, 20417314241284826. 39866963 10.1177/20417314241284826 PMC11758540

[80] N. Mandakhbayar , Y. Ji , A. El‐Fiqi , K. D. Patel , D. S. Yoon , K. Dashnyam , O. Bayaraa , G. Jin , K. Tsogtbaatar , T.‐H. Kim , J.‐H. Lee , H.‐W. Kim , Bioact. Mater. 2024, 31, 298. 37637079 10.1016/j.bioactmat.2023.08.014 PMC10458956

[81] S.‐Y. Park , J. H. Jung , D.‐S. Kim , J.‐K. Lee , B. G. Song , H. E. Shin , J.‐W. Jung , S.‐W. Baek , S. You , I. Han , D. K. Han , J. Tissue Eng. 2024, 15, 20417314231226105. 38333057 10.1177/20417314231226105 PMC10851718

[82] A. Robles‐Remacho , R. M. Sanchez‐Martin , J. J. Diaz‐Mochon , Anal. Chem. 2023, 95, 15450. 37814884 10.1021/acs.analchem.3c02029 PMC10603609

[83] C. R. Merritt , G. T. Ong , S. E. Church , K. Barker , P. Danaher , G. Geiss , M. Hoang , J. Jung , Y. Liang , J. McKay‐Fleisch , K. Nguyen , Z. Norgaard , K. Sorg , I. Sprague , C. Warren , S. Warren , P. J. Webster , Z. Zhou , D. R. Zollinger , D. L. Dunaway , G. B. Mills , J. M. Beechem , Nat. Biotechnol. 2020, 38, 586. 32393914 10.1038/s41587-020-0472-9

[84] N. Liu , D. D. Bhuva , A. Mohamed , M. Bokelund , A. Kulasinghe , C. W. Tan , M. J. Davis , Nucleic Acids Res. 2023, 52, 2. 10.1093/nar/gkad1026 PMC10783521 37953397

