# Supplementary material for: Temporal Stretch‐Induced Nuclear Mechanosensing Coordinates Early Chromatin Accessibility and Genome Protection
Source: Adv Sci (Weinh). 2025 Oct 7;13(5):e10554. doi: 10.1002/advs.202510554 (PMC12850466; doi:10.1002/advs.202510554)
Supplement: Supplementary file 1 — Supporting Information [file ADVS-13-e10554-s001.docx]

**Supplementary Data**

**I. Supplementary Figures (Fig. S1~ S32)**

**II. Supplementary Methods (Method S1~S2)**

**III. Supplementary Table (Table S1~S5)**

**I. Supplementary Figures (Fig. S1~Fig. S32)**


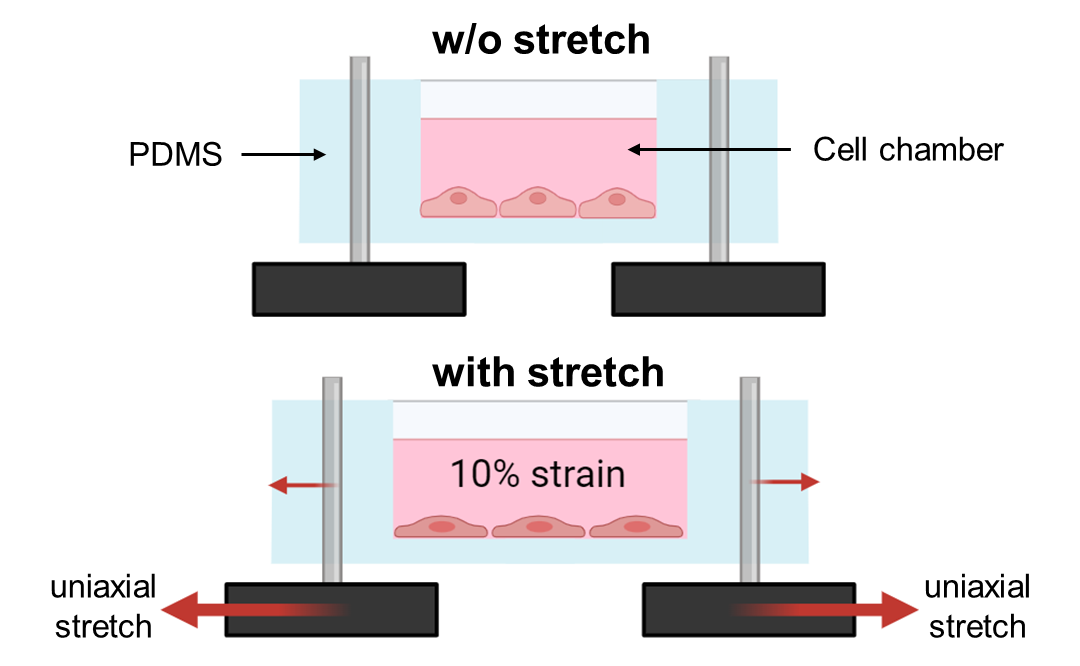


**Fig. S1.** Illustration showing the stretching setup utilized in the experiment. Cells are seeded within a PDMS chamber, and uniaxial stretch is administered by exerting force on the chamber from opposing sides.


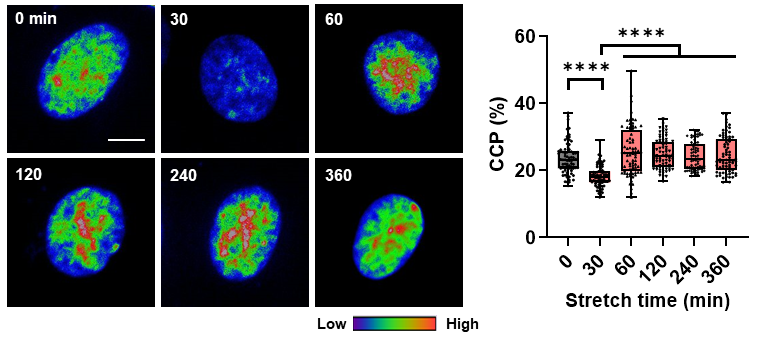


**Fig. S2.** Nuclei stained with DAPI (from Fig. 1c) were visualized using a rainbow channel, and the corresponding chromatin condensation parameter (CCP) was analyzed. Within 30 min of stretching, temporary chromatin decondensation was observed. Scale bar: 5 µm (*n*>70; *****p*<0.0001, by ANOVA with Tukey’s post hoc test).


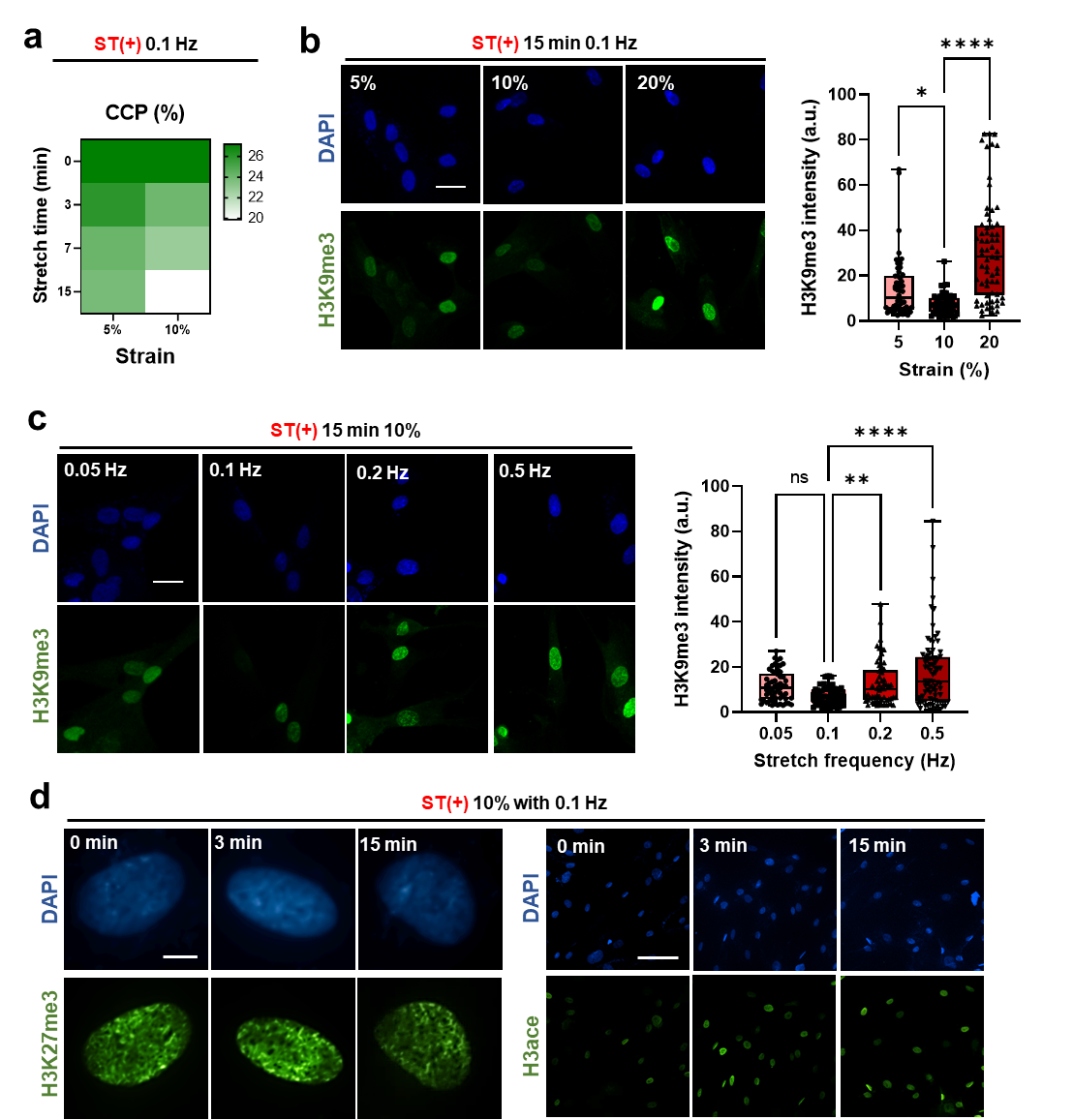


**Fig. S3.** Analysis across a range of durations and intensities of strain demonstrates the general applicability of stretch-induced epigenetic remodeling. (a) Heatmap depicts chromatin condensation parameter (CCP) across various stretching conditions (strain and time) with 0.1 Hz, highlighting maximal reduction at 10% strain and 15 min duration (*n*>40). (b, c) Quantitative analysis of immunofluorescence staining images revealed optimal H3K9me3 reduction at 10% strain amplitude (range tested: 5–20% with 0.1 Hz) and 0.1 Hz frequency (range tested: 0.05-0.5 Hz with 10%) following 15 min mechanical stretching (*n*>50; *****p*<0.0001, ns: not significant *p*>0.05, by ANOVA with Tukey’s post hoc test). Scale bar: 20 µm. (d) Representative immunofluorescence staining images, showing the changes in H3K27me3 and H3ace levels at 3 and 15 min post-stretch (10% strain and 0.1 Hz). Scale bar: 5 µm. Scale bar: 5 µm (left), 100 µm (right).


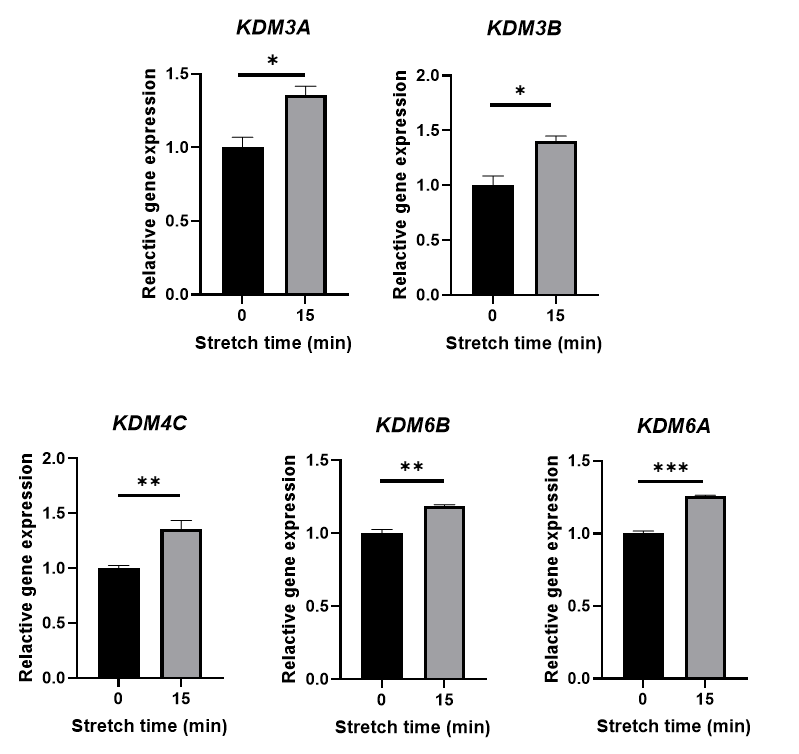


**Fig. S4.** Gene expressions in histone lysine demethylases by qPCR, before and after stretch for 15 min (*n*=3, **p*<0.05, ***p*<0.01, ****p*<0.001, by Student’s t-test).

**Fig. S5.** Heatmap representation of gene expressions in histone methyl transferases by qPCR, comparing 0 min versus 15 min of stretch (*n*=3), shows little notable difference.


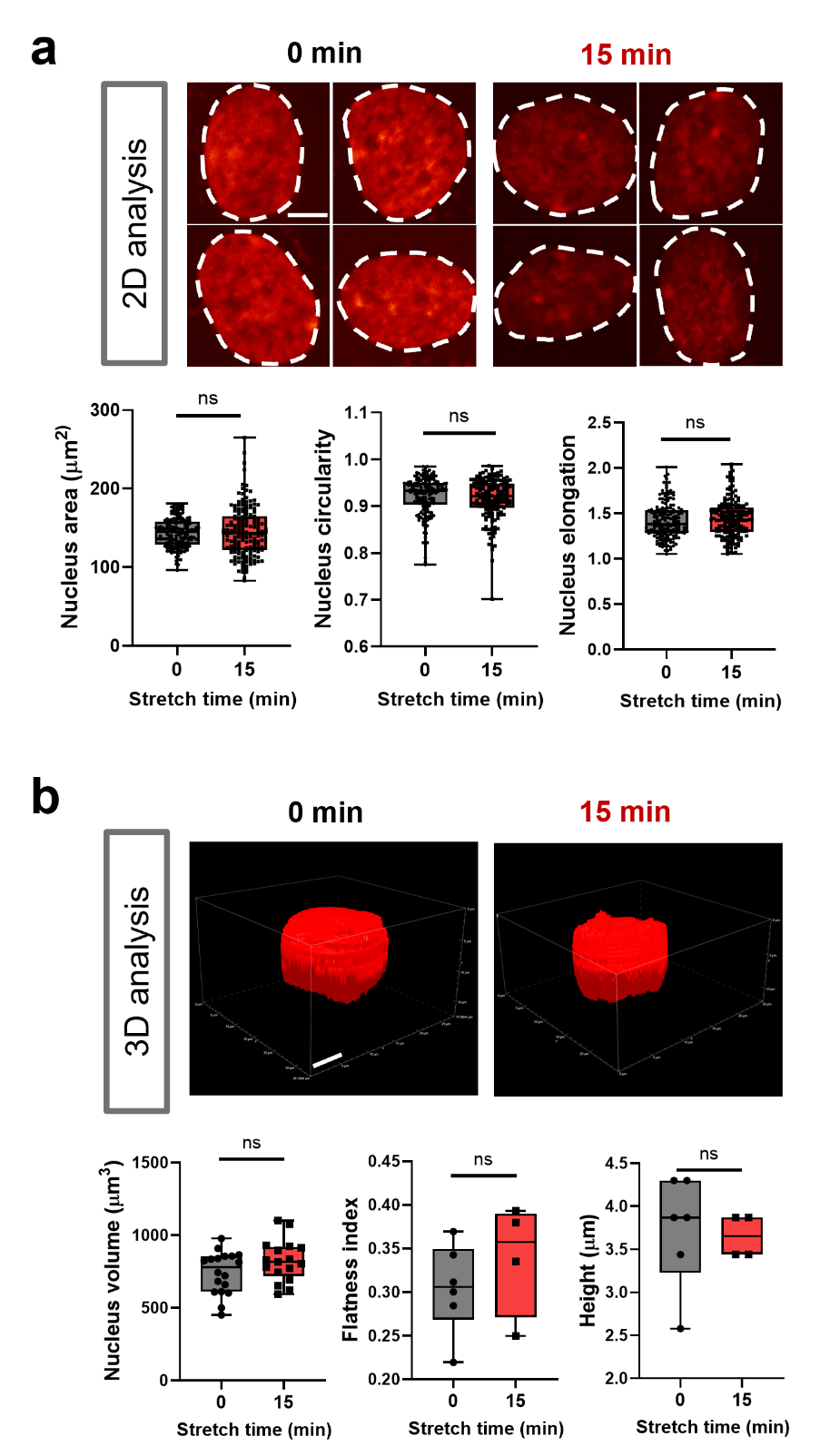


**Fig. S6.** Short-term stretch induces epigenetic changes without significantly altering nuclear morphology and volume. (a) Analysis of DAPI-stained nucleus 2D projection images and quantification graphs of nuclear area, circularity, and elongation, demonstrating stretch did not induce nucleus morphological changes. Scale bar: 5 µm. (*n*=170). (b) Examination of 3D nucleus images via confocal z-stack imaging and quantification graphs of nucleus volume (*n*=22), flatness index (*n*>4), and height (*n*>4) revealing no significant alterations in 3D nucleus volume and morphology. (ns: not significant *p*>0.05, by student’s t-test).


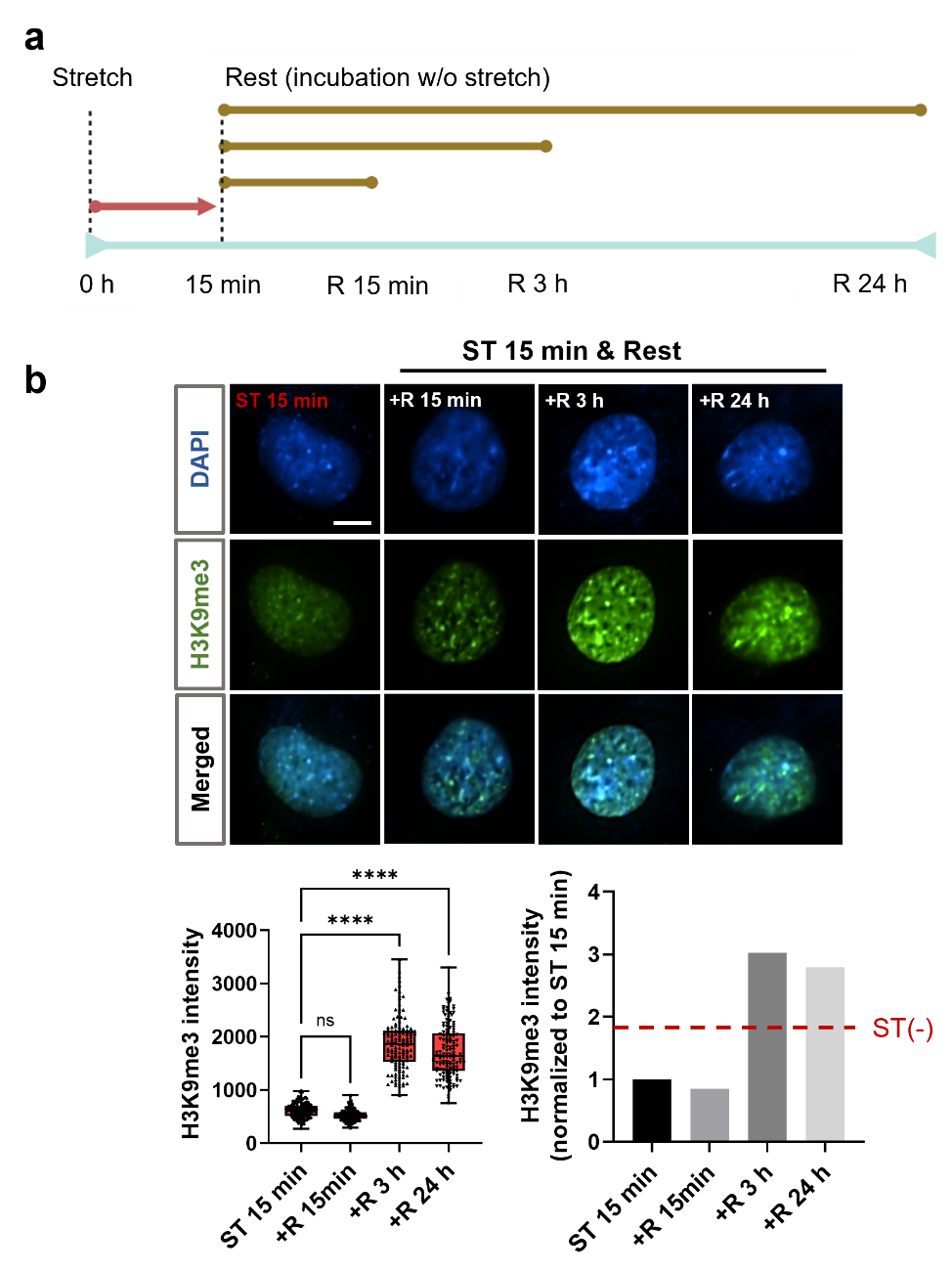


**Fig. S7.** Stretch-induced H3K9me3 decrease gradually recovers during resting periods (lasting up to 24 h). (a) A schematic showing the timeline of the stretch-and-rest experiment. (b) Immunofluorescence staining of H3K9me3 at various rest periods and semi-quantification of intensity reveals that the recovery effect becomes more pronounced with longer resting durations (3 h and 24 h, not 15 min). Scale bar: 5 µm. (*n*=139; *****p*<0.0001, ns: not significant *p*>0.05, by ANOVA with Tukey’s post hoc test). Red dot line (‘ST(-)’) indicates pre-stress value (0 min) of H3K9me3 for comparison.


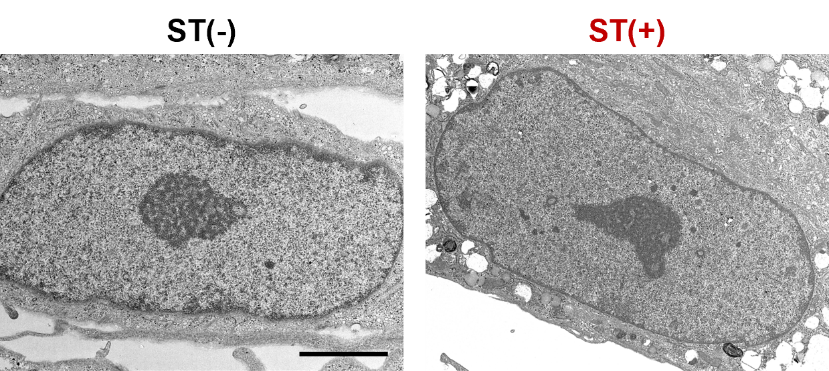


**Fig. S8.** Low-magnification images of transmission electron microscopy (TEM), used to analyze the nuclear periphery. Scale bar: 2 µm.


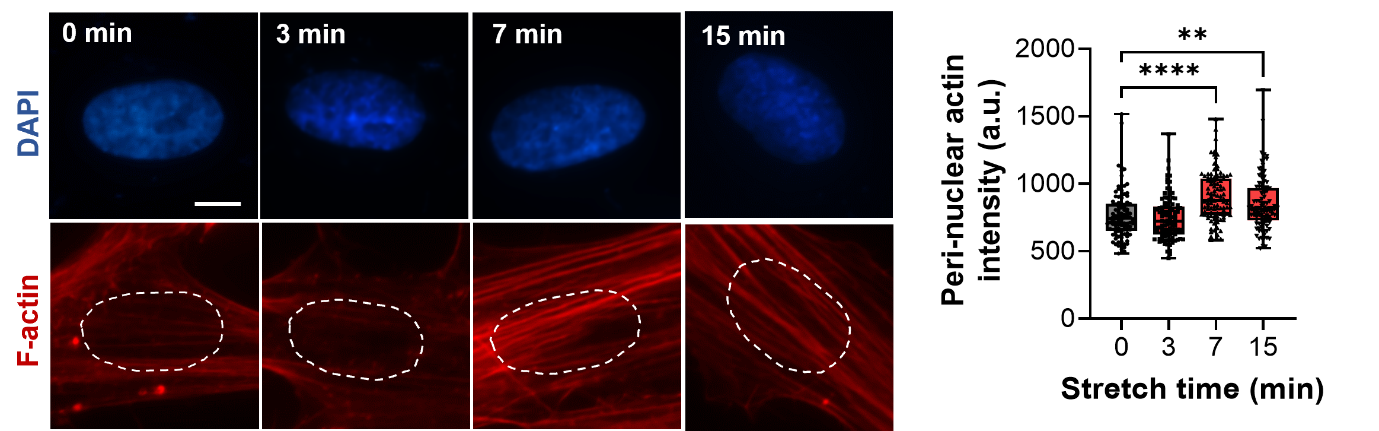


**Fig. S9.** DAPI (nucleus) and phalloidin (F-actin) staining images captured over various stretch durations, alongside semi-quantification of perinuclear actin, demonstrate rapid stretch-induced actin remodeling, reaching its peak as early as 7 min. (*n*>100; *****p*<0.0001, ***p*<0.01, by ANOVA with Tukey’s post hoc test). Scale bar: 5 µm.


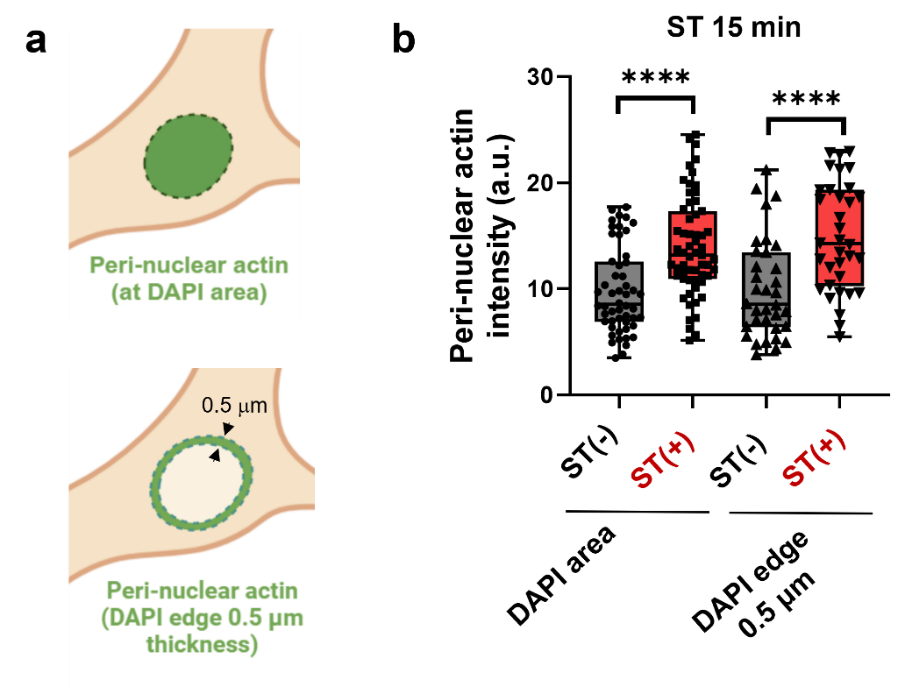


**Fig. S10.** Phalloidin-stained perinuclear actins were analyzed either within the DAPI area or at the DAPI periphery with a 0.5 µm thickness; (a) Schematic showing the analysis area, and (b) semi-quantified intensities, showing significantly higher levels with stretch (15 min) at both regions (*n*>34; *****p*<0.0001, by Student’s t-test).


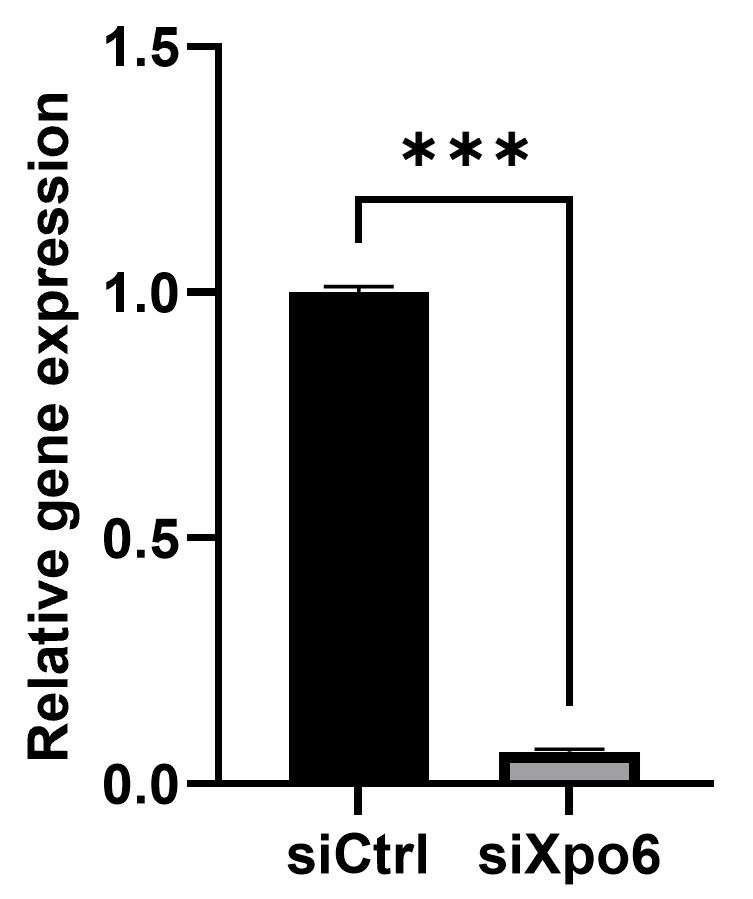


**Fig. S11.** The efficiency of exportin-6 gene expression suppression by siXpo6 was verified by qPCR, with gene expression downregulated by more than 90% (****p*<0.001, by Student’s t-test).


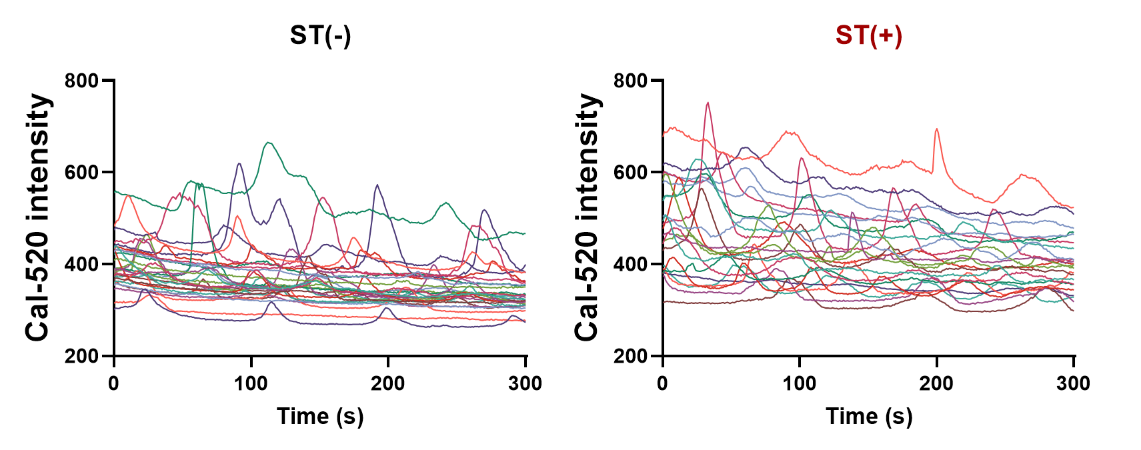


**Fig. S12.** Quantification graph of the Cal-520 AM live imaging heatmap (in Fig. 3a) showing highly increased frequency of Ca^2+^ release under stretched condition.


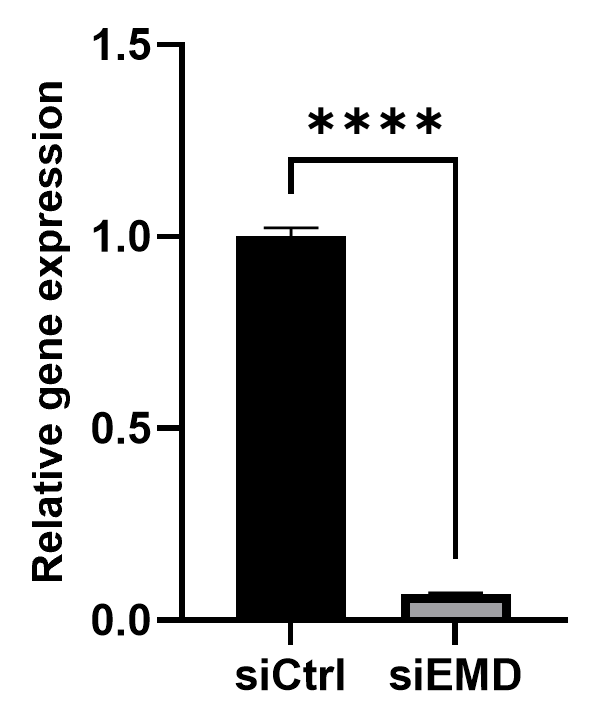


**Fig. S13.** The efficiency of emerin gene expression suppression by siEMD was verified by qPCR, with gene expression downregulated by more than 90% (*****p*<0.0001, by Student’s t-test).


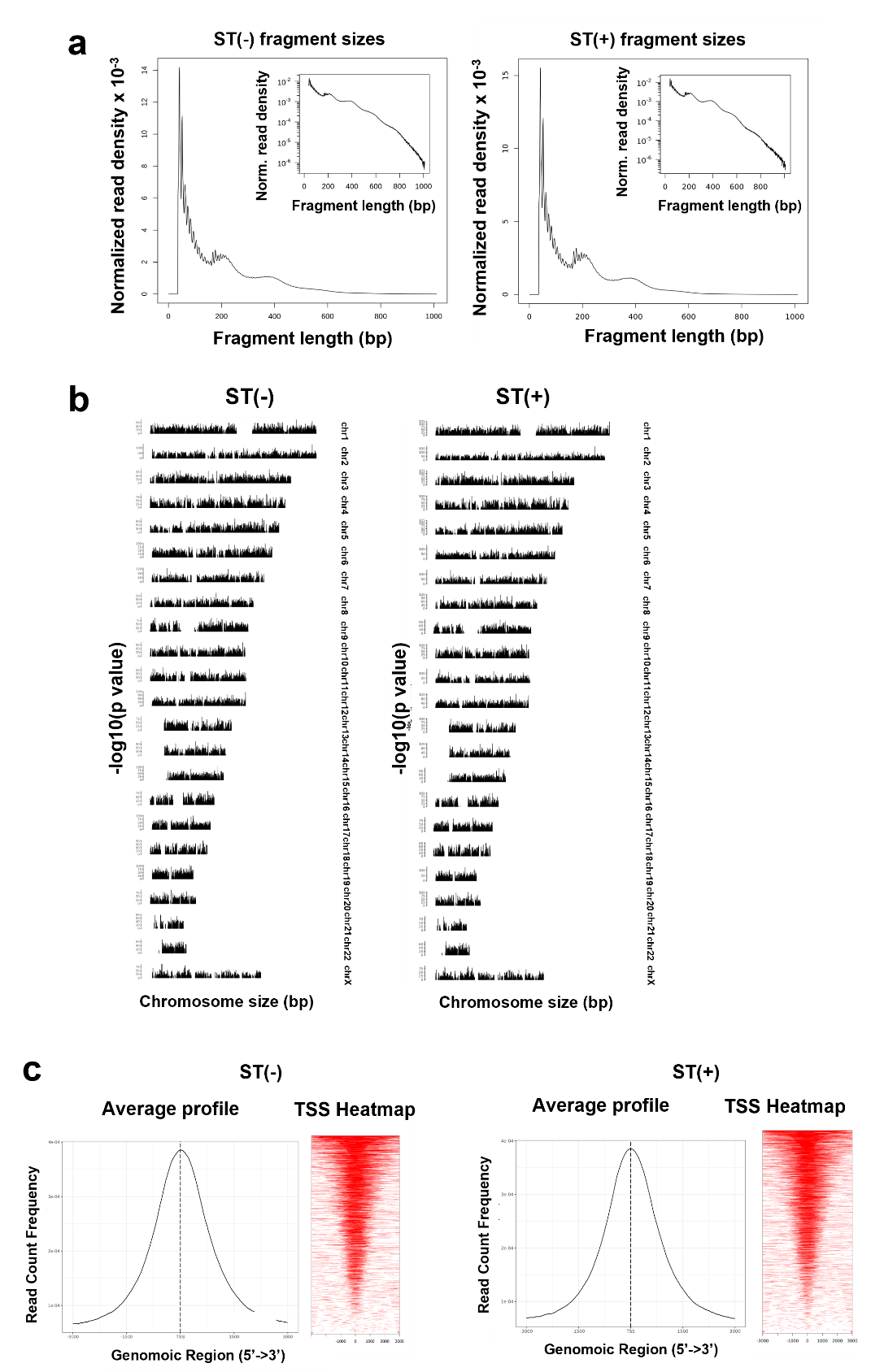


**Fig. S14.** Overview of assay for transposase-accessible chromatin using sequencing (ATAC-seq) datasets. (a) A typical fragment size distribution plot showing enrichment around 100 and 200 bp, indicating nucleosome-free and mono-nucleosome-bound fragments. Additional periodic peaks at 400 and 600 bp, corresponding to di- and tri-nucleosomes, were similarly detected in both groups, confirming the successful execution of the ATAC-seq experiment. (b) Coverage plot illustrating global chromatin accessibility in ST(-) and ST(+). (c) Average profile, TSS heatmap normalized to counts per million mapped reads, coverage plot, and fragment size distribution. The heatmap shows global chromatin accessibility in ST(-) and ST(+). TSS: transcription start site.


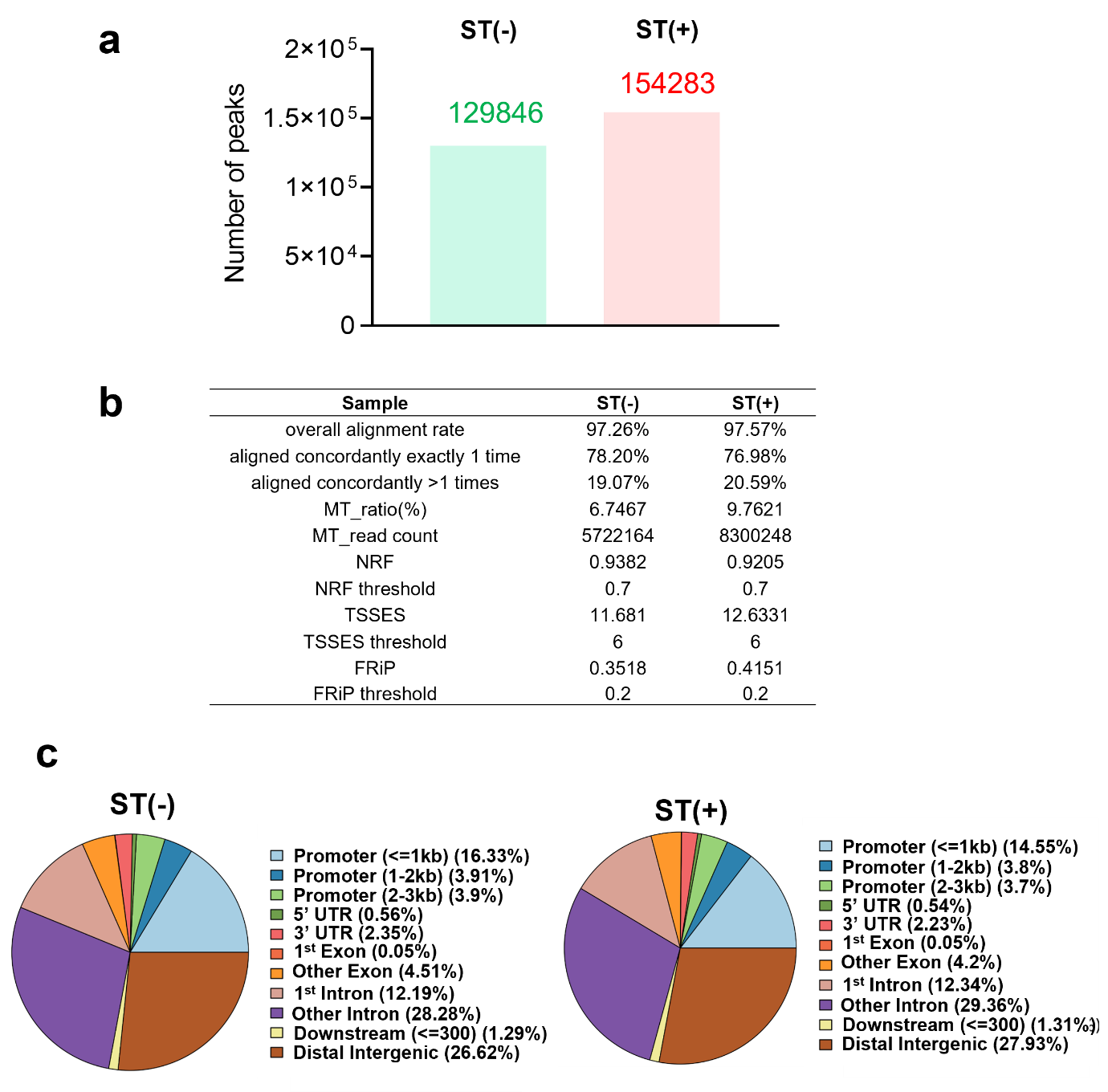


**Fig. S15.** Overview of the assay for transposase-accessible chromatin using sequencing (ATAC-seq) datasets. (a) Peak calling numbers for each group determined by MACS2 (version 2.1.1.20160309). (b) ATAC-seq quality control metrics from DAVID analysis. (c) Typical peak annotation pie chart showing that more than half of the peaks are located in enhancer regions (distal intergenic and intronic regions), with only around 23% in promoter regions, similarly detected in both groups.


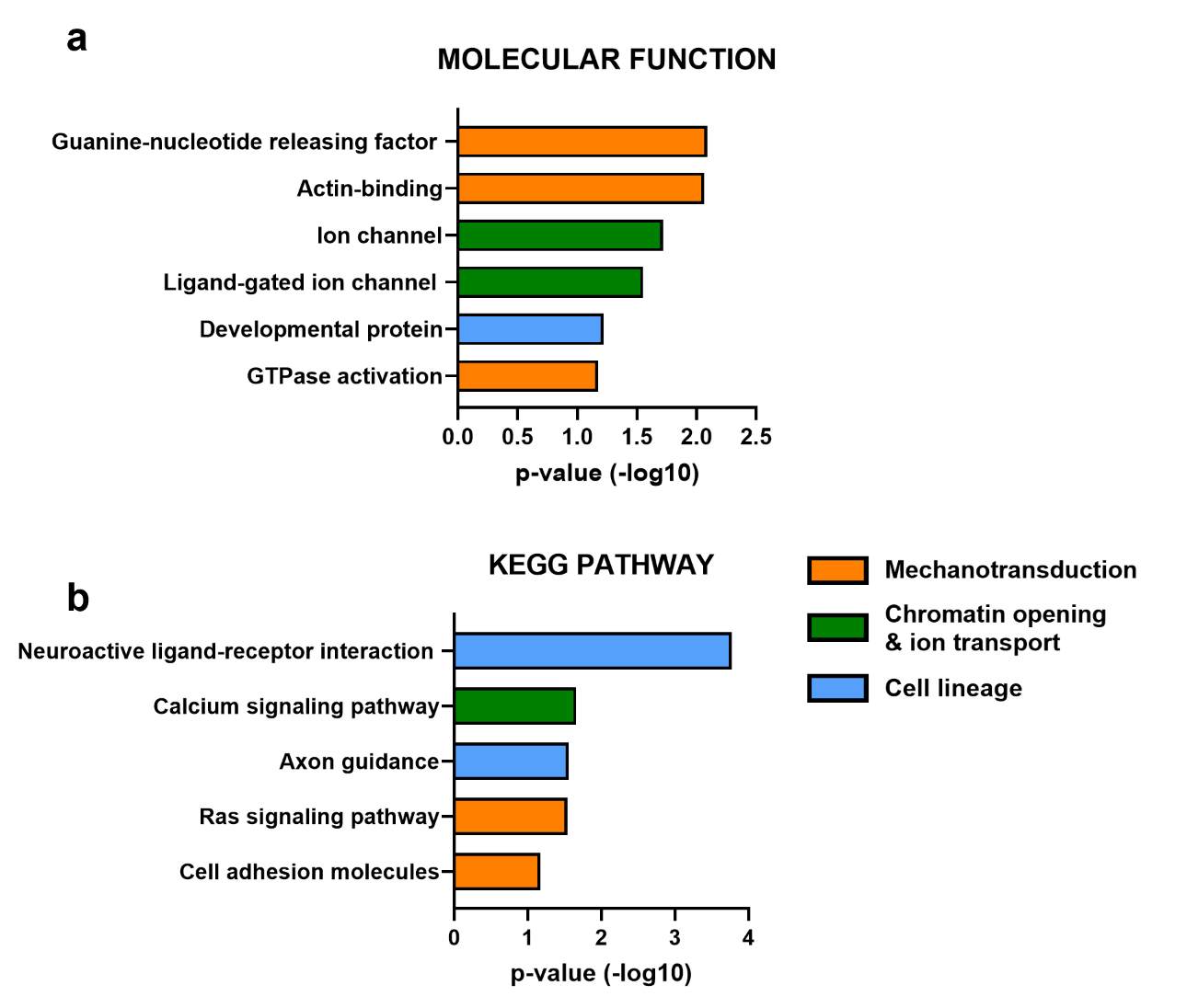


**Fig. S16.** DAVID analysis of highly accessible genes in ST(+) versus ST(-). (a) Molecular function and (b) KEGG pathway analysis identifies those associated with enhanced mechanotransduction, increased multipotency for lineage specification, and chromatin opening.


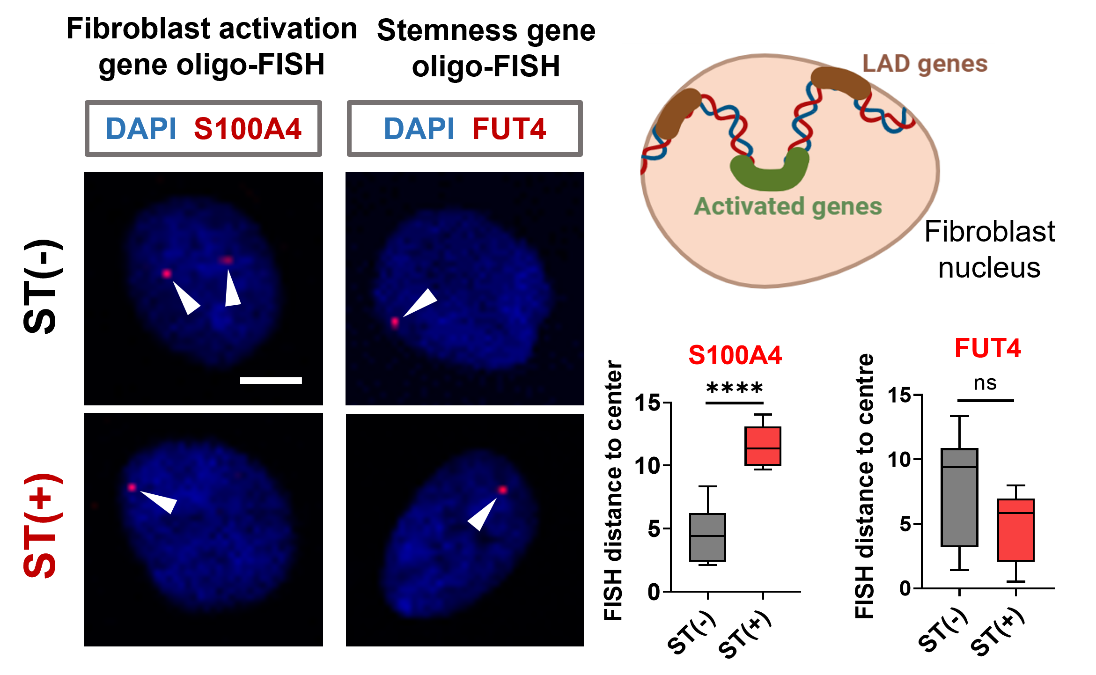


**Fig. S17.** DNA oligonucleotide fluorescence in situ hybridization (FISH) experiments show that the fibroblast activation gene (*S100A4*), rather than stemness gene (*FUT4*), becomes located at the nuclear edge by stretch, indicating fibroblast gene repression. (*****p*<0.0001, ns: not significant *p*>0.05, by Student’s t-test). Scale bar: 5 µm.


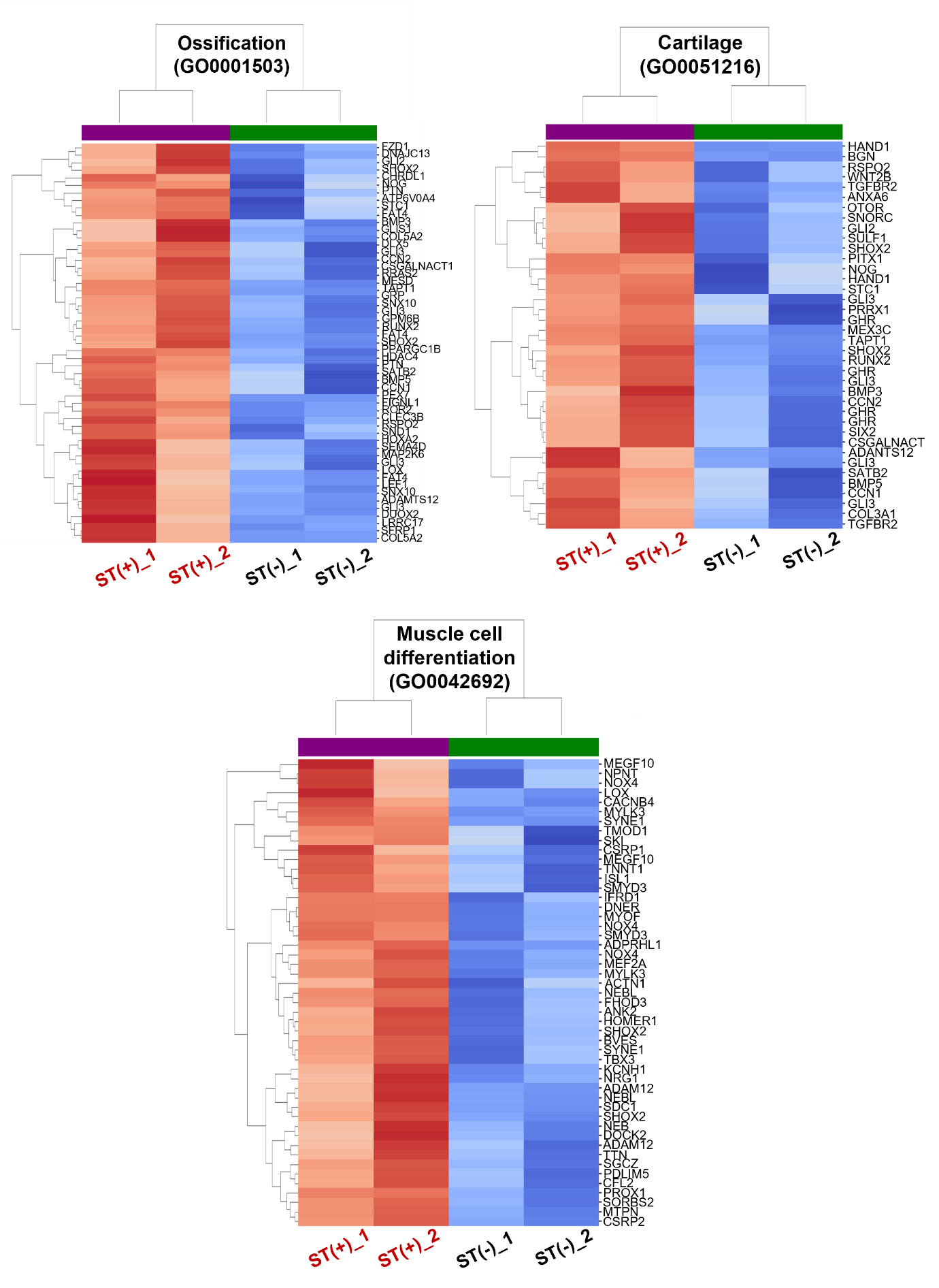


**Fig. S18.** Heatmap analysis of GO terms related to ‘ossification’, ‘cartilage’, and ‘muscle cell differentiation’, demonstrating stretched cells (ST(+)) had more accessible regions versus unstretched cells (ST(-)).


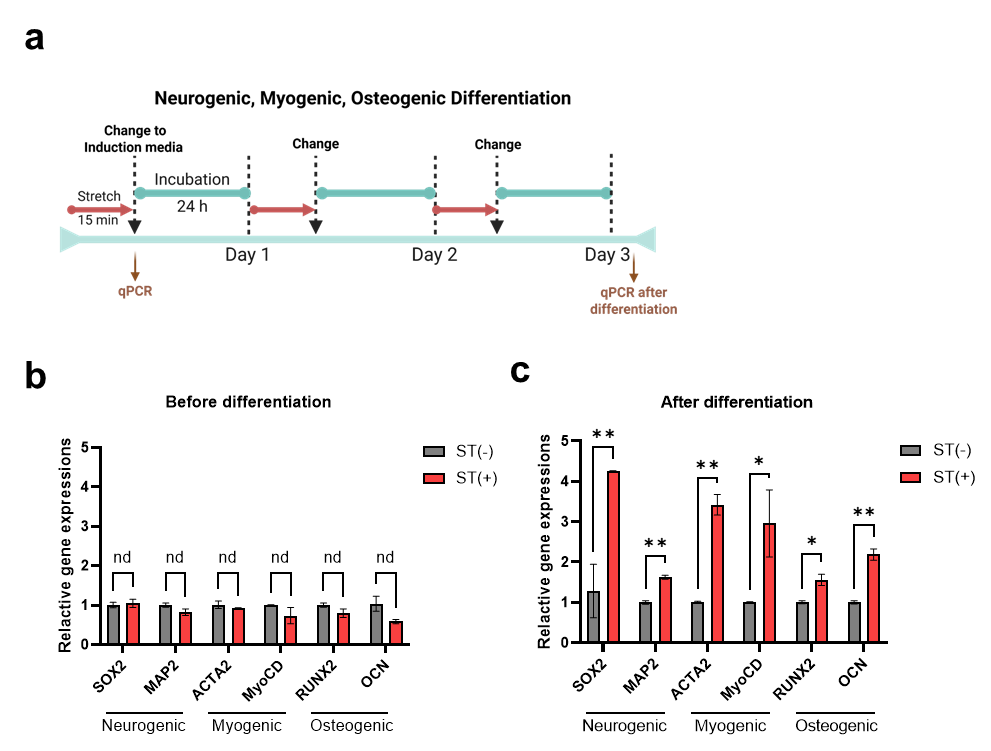


**Fig. S19 .** Capacity of stretched fibroblasts to exhibit traits of different lineages (neurogenic, myogenic, and osteogenic cells). (a) Schematic showing experimental schedule. Cells were cultured in lineage-specific (neurogenic, myogenic, or osteogenic) medium under stretch for 15 min per day. (b) qPCR results show that there is no significant change in lineage-specific gene expression before the addition of biochemical cues. (c) With appropriate biochemical cues, significantly higher expressions of neurogenic, myogenic, and osteogenic markers were noted in the stretched group. (*n*=3; nd: statistically not determined *p*>0.05, **p*<0.05, ***p*<0.01, by Student’s t-test).


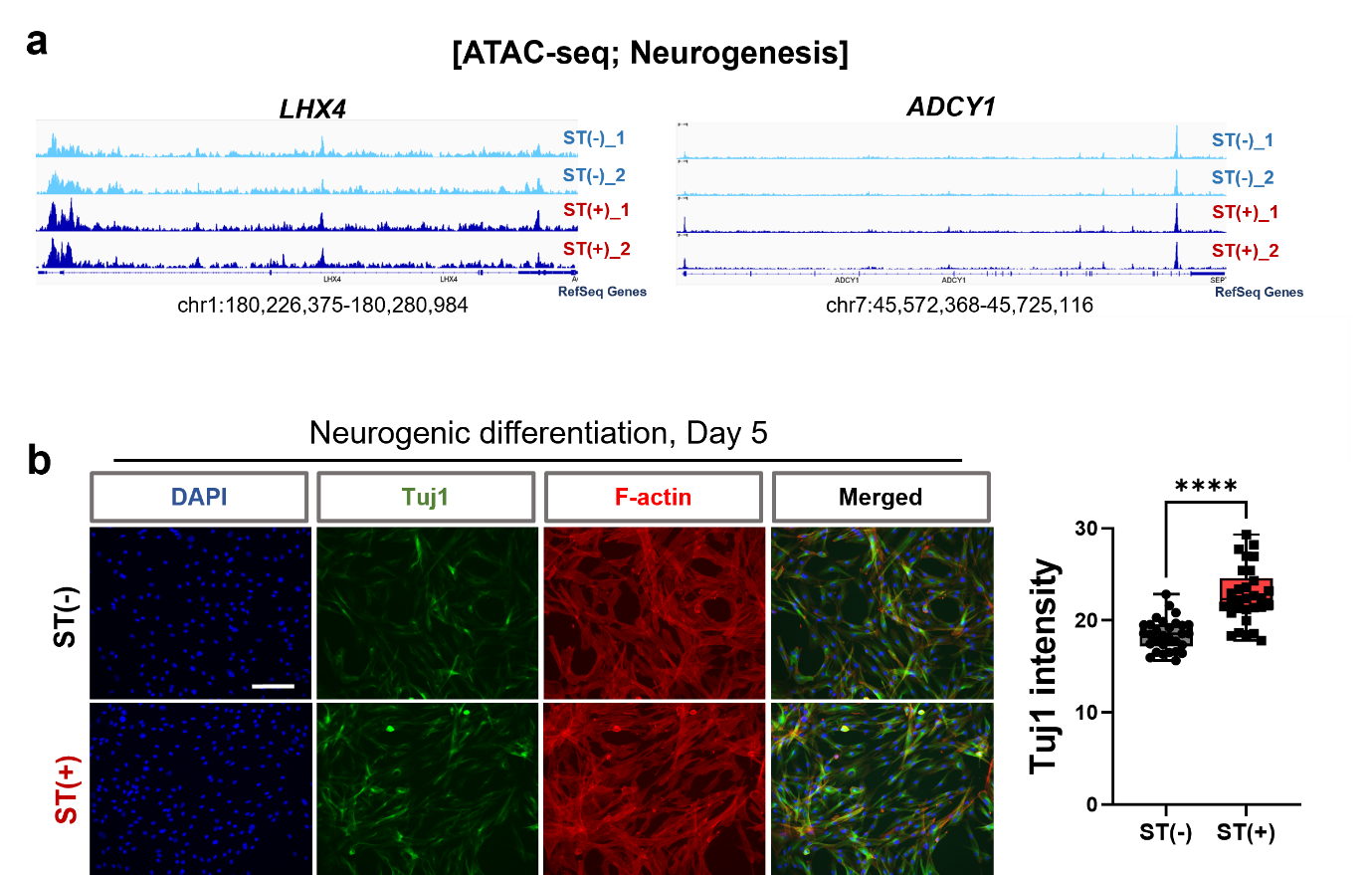


**Fig. S20.** Capacity of stretched fibroblasts to exhibit neurogenic trait. (a) Representative transcription factor genome browser tracks of regions associated with neurogenesis (*LHX4, ADCY1*), showing higher accessibility in stretched cells versus unstretched cells. (b) Tuj1 and F-actin immunofluorescence staining images and semi-quantification showing that the stretched group has higher Tuj1 expression compared to the unstretched group for 5 days of culture in neurogenic medium. Scale bar: 100 µm. (*n*=30; *****p*<0.0001, by Student’s t-test).


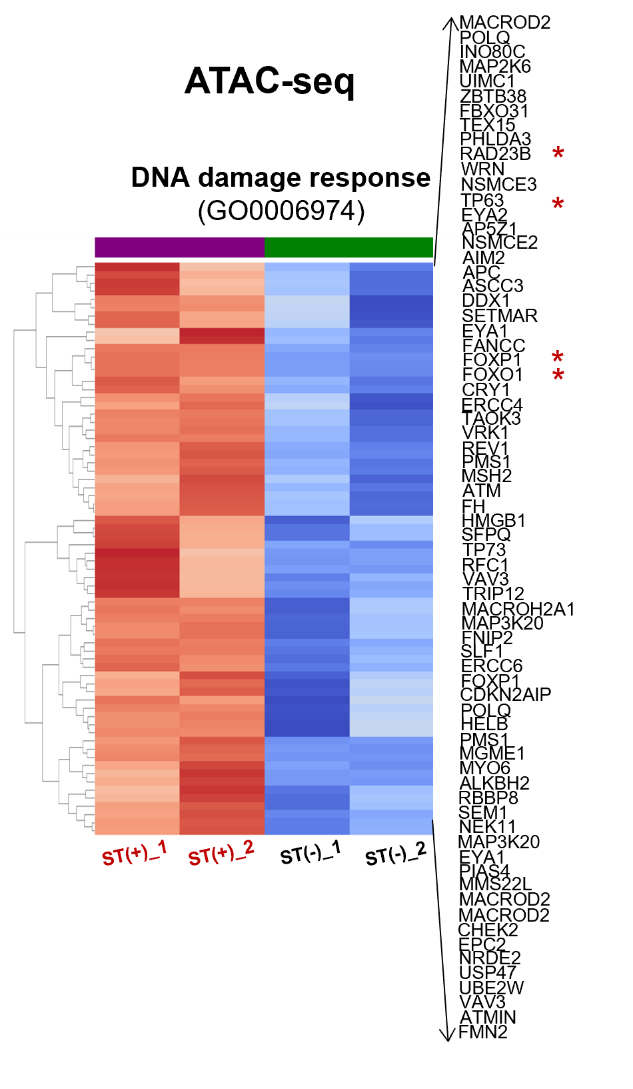


**Fig. S21.** ATAC-seq heatmap analysis of GO terms related to the DNA damage response, revealing increased accessibility in ST(+) compared to ST(-). Some representative genes are highlighted with asterisks.


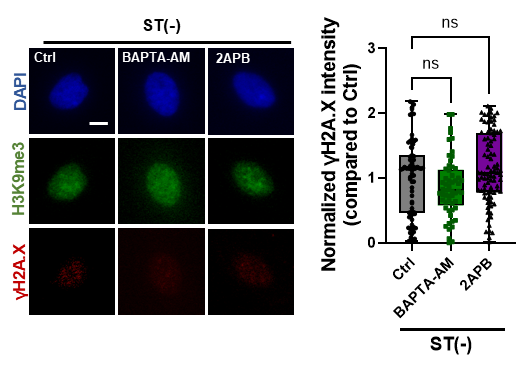


**Fig. S22.** Immunofluorescence staining and semi-quantitative analysis of H3K9me3 and γH2A.X intensity demonstrate that DNA damage does not occur in the absence of mechanical stretching, even when intracellular calcium release is blocked by chemicals (BAPTA-AM or 2APB). Scale bar: 5 µm. (*n*>60; ns: not significant, *p*>0.05, by ANOVA with Tukey's post hoc test).


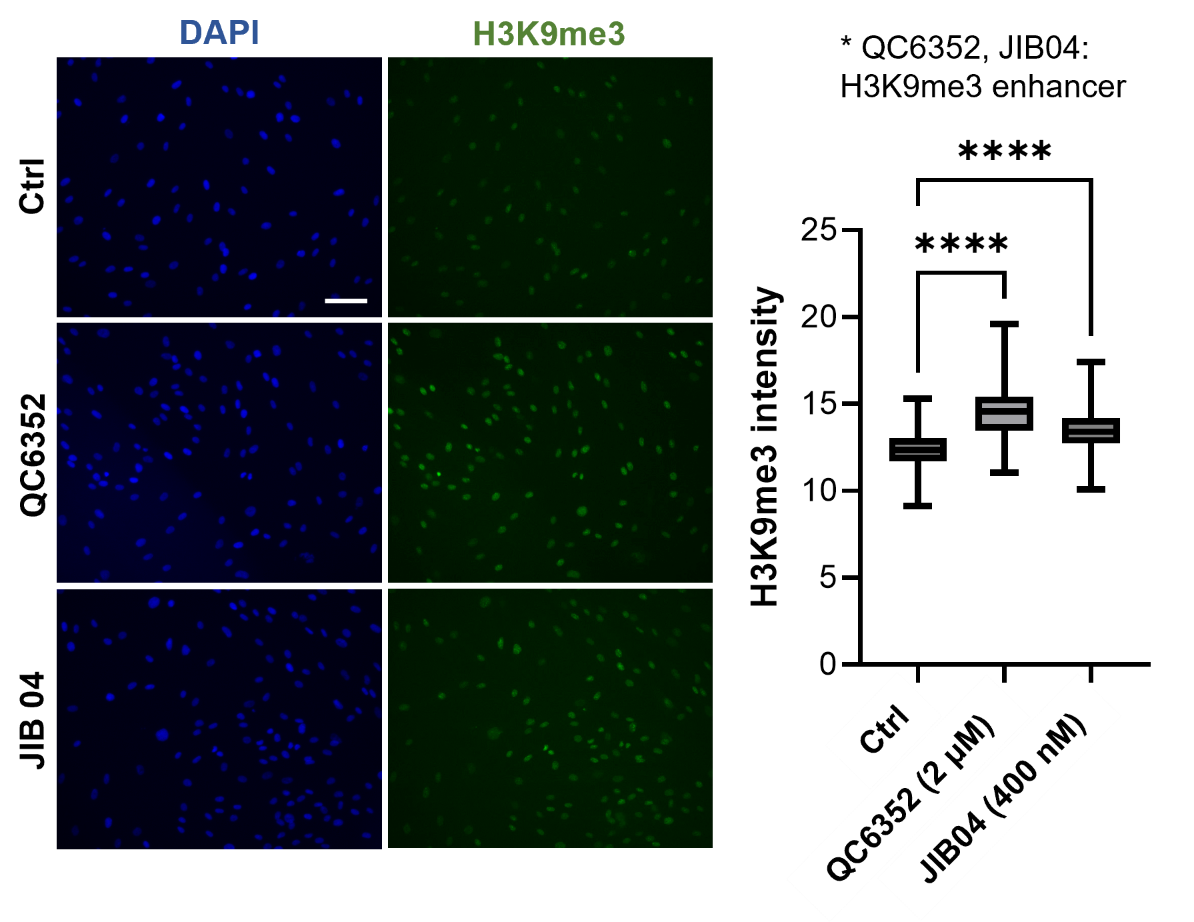


**Fig. S23.** H3K9me3 immunofluorescence staining images and semi-quantification showing the increase in H3K9me3 expression following the treatment of H3K9me3 enhancers, QC6352 (2 μM) and JIB04 (400 nM), compared to control (Ctrl). Scale bar: 100 µm. (*n*>216; *****p*<0.0001, by ANOVA with Tukey’s post hoc test).


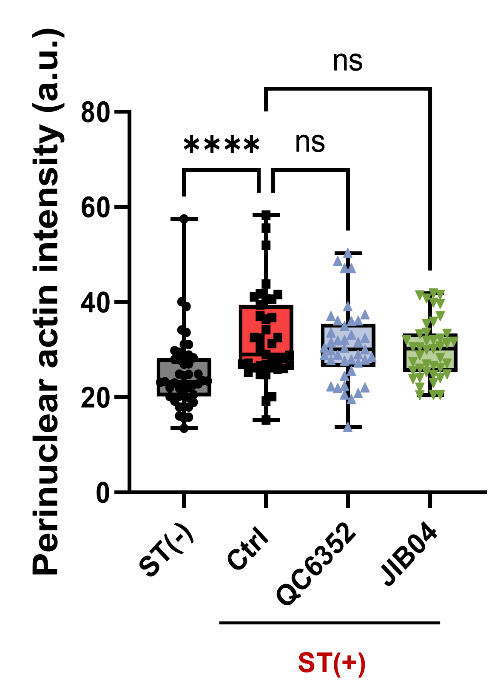


**Fig. S24.** Effect of H3K9me3 enhancers (QC6352 and JIB04) on perinuclear actin formation (*n*>40; *****p*<0.0001, ns: not significant, *p*>0.05, by ANOVA with Tukey’s post hoc test).


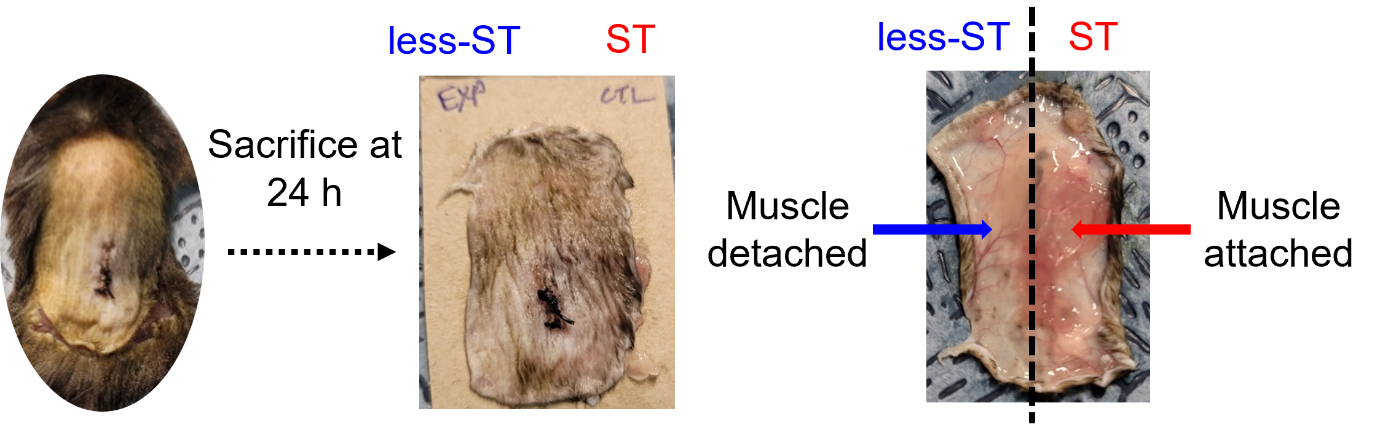


**Fig. S25.** Surgical procedures to obtain stretched (“ST”, normal) and less-stretched (“less-ST”, due to physical detachment of the underneath muscle) skin tissue. To create the less-ST condition, a surgical intervention was performed to detach the underlying muscle from the skin, thereby reducing the mechanical tension typically generated by muscle contraction and physical contact. The ST and less-ST skin tissues were harvested from the same individual to enable direct comparison (*n*=3). Mice were sacrificed 24 h post-surgery.


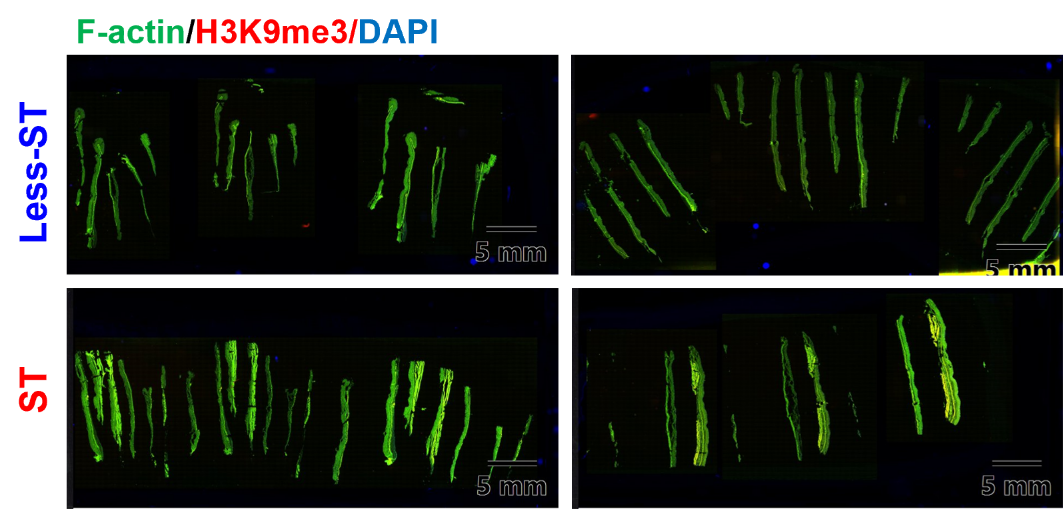


**Fig. S26.** Scanning images of stretched (ST) and less-stretched (less-ST) skin tissue samples, after staining for F-actin (green), H3K9me3 (red), and DAPI (blue). Mice were sacrificed 24 h post-surgery (*n*=3).


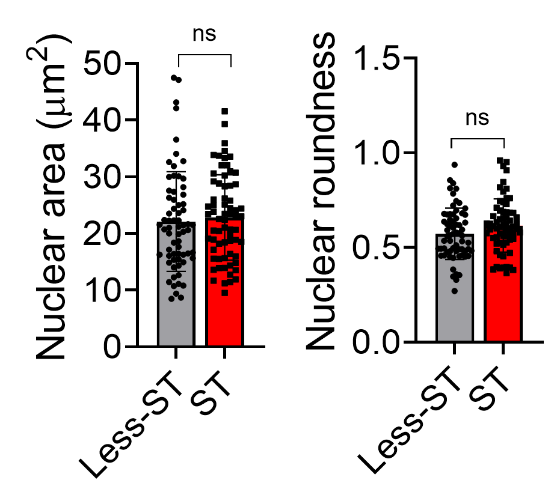


**Fig. S27.** Nucleus area and roundness of dermal fibroblasts in *in vivo* conditions. Quantification of 2D nucleus-stained images (DAPI) from stretched (ST) and less-stretched (less-ST) skin tissue. Surgical intervention (less-ST) induced no significant changes in nucleus area and roundness, similar to the in vitro studies. (ns: not significant *p*>0.05, by Student’s t-text).


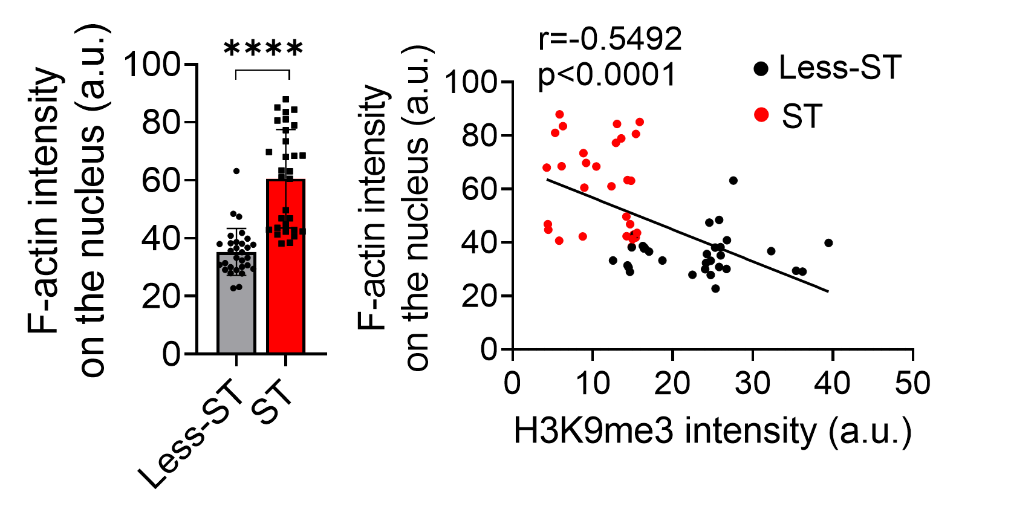


**Fig. S28.** F-actin intensity on nucleus and its correlation with H3K9me3 intensity. Semi-quantification of staining images (F-actin and H3K9me3) from stretched (ST) and less-stretched (less-ST) skin tissue. The surgical intervention (less-ST) resulted in decreased perinuclear F-actin intensity, which showed an inverse correlation with H3K9me3 expression levels. (*****p*<0.0001, by Student’s t-test).


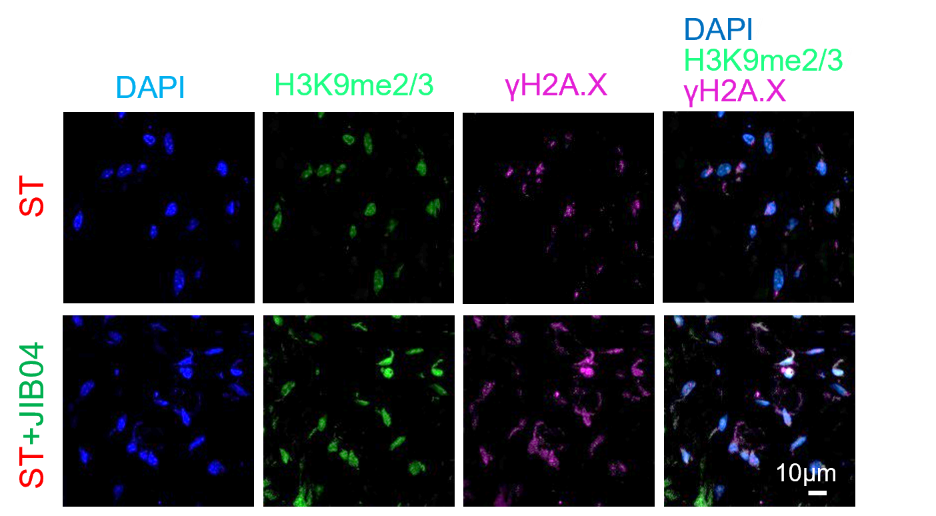


**Fig. S29.** Representative co-staining images of the DNA damage marker γH2AX and heterochromatin marker H3K9me2/3 (used in place of H3K9me3 due to antibody host compatibility), with DAPI as the nuclear counterstain. Tissue cells were subjected to physiological mechanical stretch (ST) or combined with treatment of H3K9me3 enhancer JIB04 (ST+JIB04).


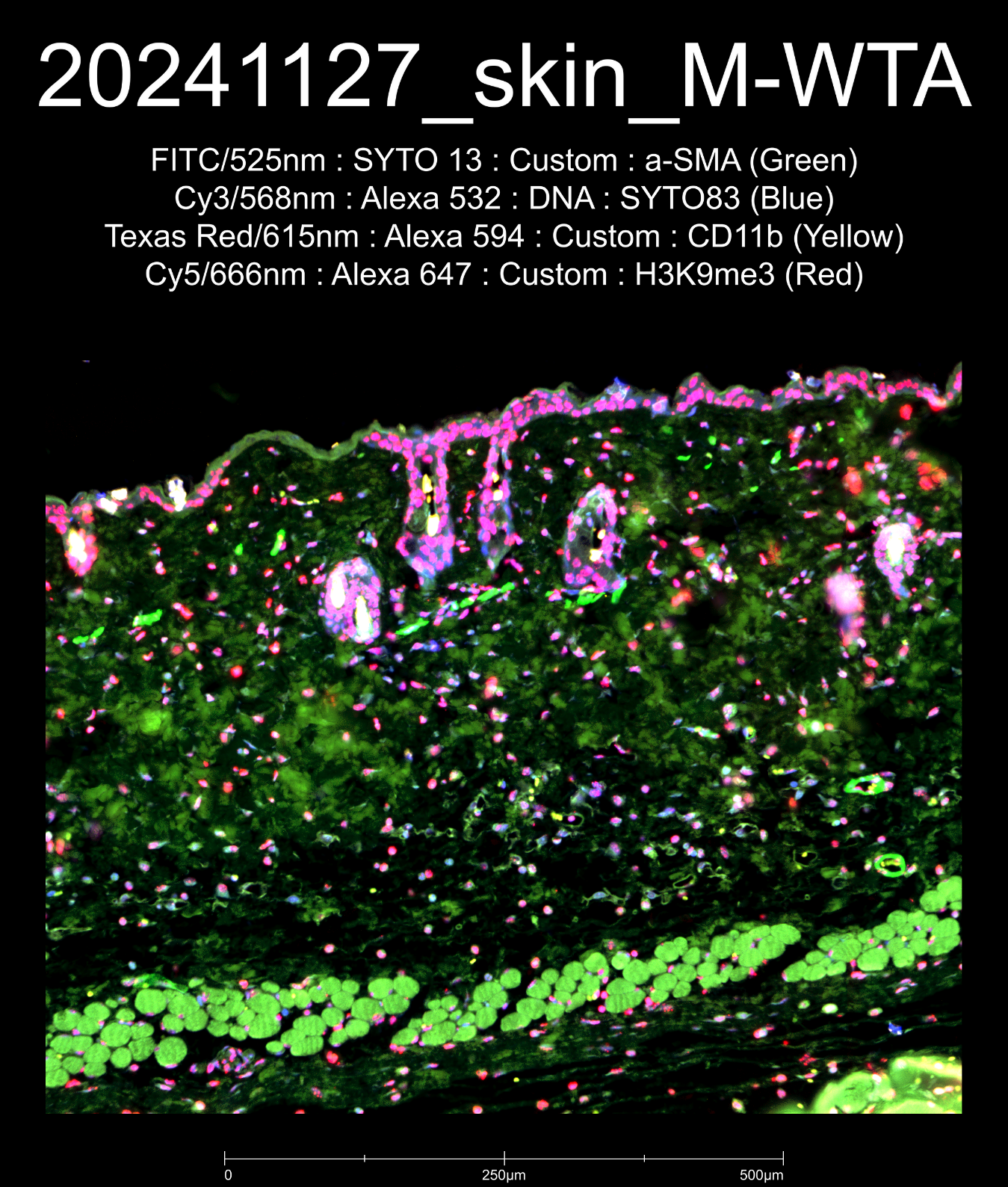


**Fig. S30.** High-power view of representative in vivo dermal fibroblasts immunostained and analyzed by GeoMx spatial transcriptomics (enlarged region from Fig. 9i).


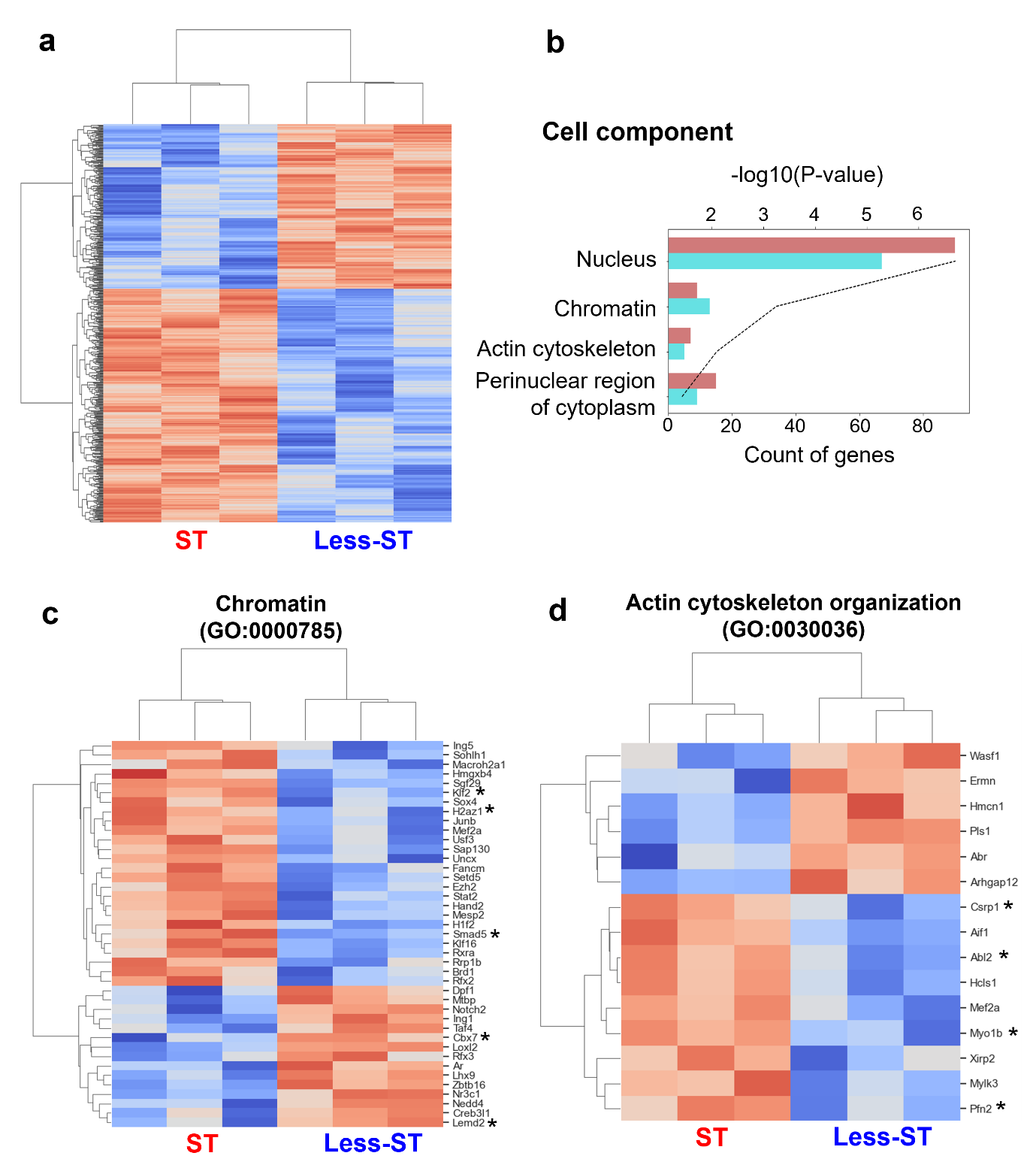


**Fig. S31.** Transcriptomic analysis of dermal fibroblasts from stretched and less-stretched skin using GeoMx. (a) Hierarchical clustering heatmap of differentially expressed genes (fold change > 1.3, normalized log2 > 2, *p*< 0.05, *n*=3). (b) Cellular component enrichment analysis using DAVID. (c) Gene ontology heatmap highlighting chromatin and actin cytoskeleton-related pathways. Asterisks denote key regulatory genes.


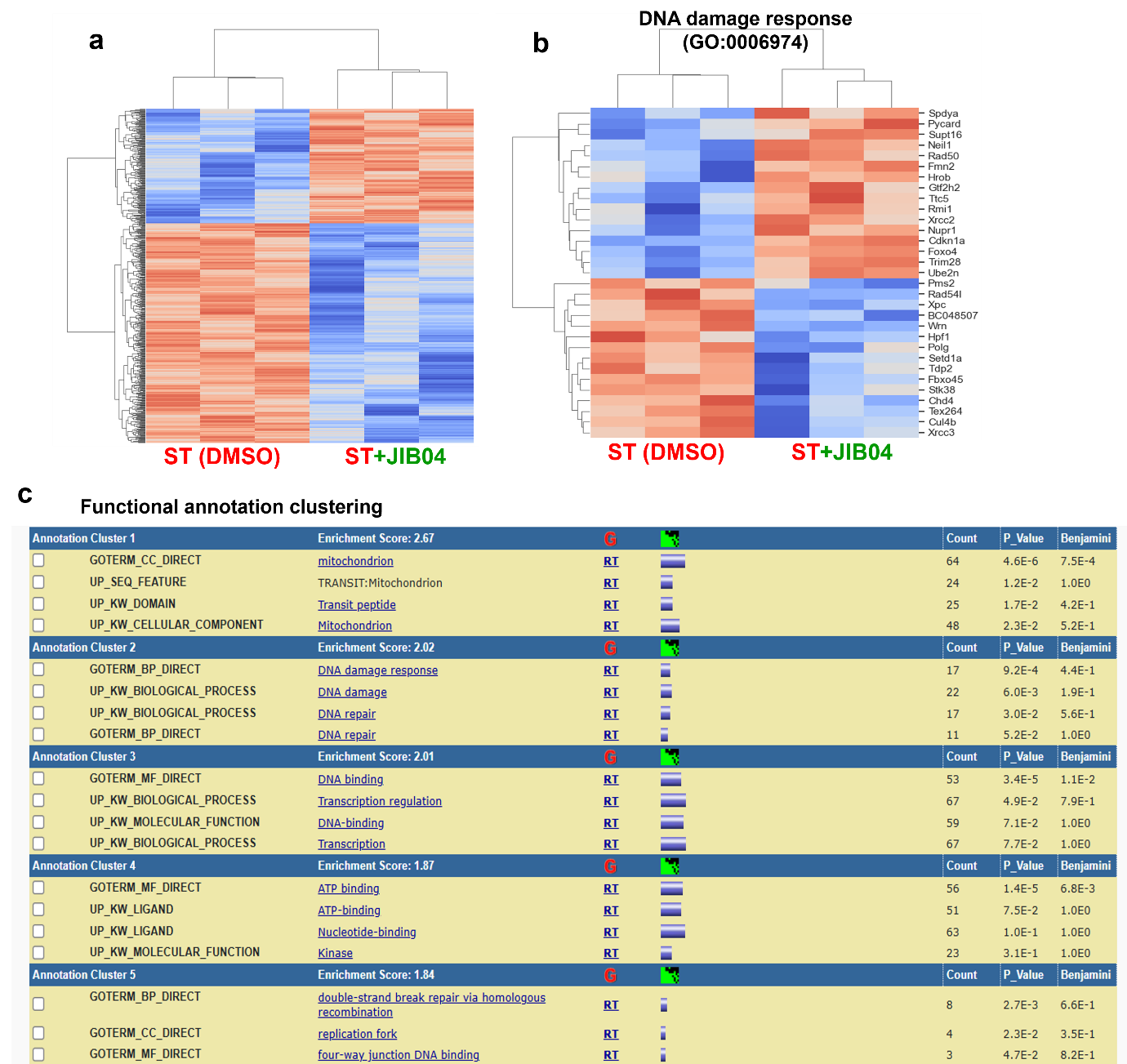


**Fig. S32.** Transcriptomic comparison of dermal fibroblasts from stretched skin tissue treated with DMSO (control) or JIB04. (a) Hierarchical clustering heatmap illustrating differentially expressed genes between the control group (ST(DMSO)) and the JIB04-treated group (ST+JIB04). Inclusion criteria: fold change > 1.3, normalized log2 expression > 2, and *p*< 0.05 (*n*=3). (b) Gene ontology heatmap showing pathways enriched for DNA damage response processes, with notable variations between the two treatment groups. (c) Top five functional annotation clusters, emphasizing the involvement of DNA damage response pathways and their modulation by JIB04.

**II. Supplementary Methods (Method S1~S2)**

**Method S1: Procedures for computational modeling**

In this Supplementary Method section, we provide a detailed description of our model for nucleus bursting by compression force from stretched F-actin cables and compression force dissipation in a viscoelastic nucleus with changes in chromatin status. This includes a discussion of the main assumptions, equations, and methods employed in our computational models, which build upon recent advances in modeling the mechanical behavior of cells and nuclei^1^.

Our experiments revealed that substrate stretching up to 10% strain for 15 min did not induce DNA damage when accompanied by a loss of the repressive histone mark H3K9me3. However, when the loss of H3K9me3 was interrupted by chemical treatment (H3K9me3 enhancer), DNA damage was detected. These findings align with recent studies highlighting the role of chromatin modifications in regulating nuclear mechanics and DNA damage response^2^. To elucidate the role of H3K9me3 loss in mitigating stretch-induced DNA damage, we first modeled the compression force on the nucleus exerted by elastic F-actin cables. Our computational approach is inspired by previous work that modeled the mechanical interplay between the cytoskeleton and the nucleus^3^.

Computational simulations revealed that stretch-induced increases in F-actin cable number and total thickness could generate enhanced compression force, resulting in nuclear bursting when the nucleus was considered an elastic material. This aligns with experimental observations of nuclear deformation and rupture under extreme mechanical stress^4,5^. However, when the viscoelastic properties of the nucleus were adjusted to account for a reduced (~50%) nuclear stiffness due to the rapid loss of H3K9me3, compression force dissipation occurred, preventing DNA damage. This finding is supported by recent studies demonstrating the role of chromatin modifications in modulating nuclear viscoelasticity and mechanical stability^6^.

Our computational modeling approach provides a mechanistic framework for understanding how chromatin modifications, such as the loss of H3K9me3, can protect the genome from mechanical damage.


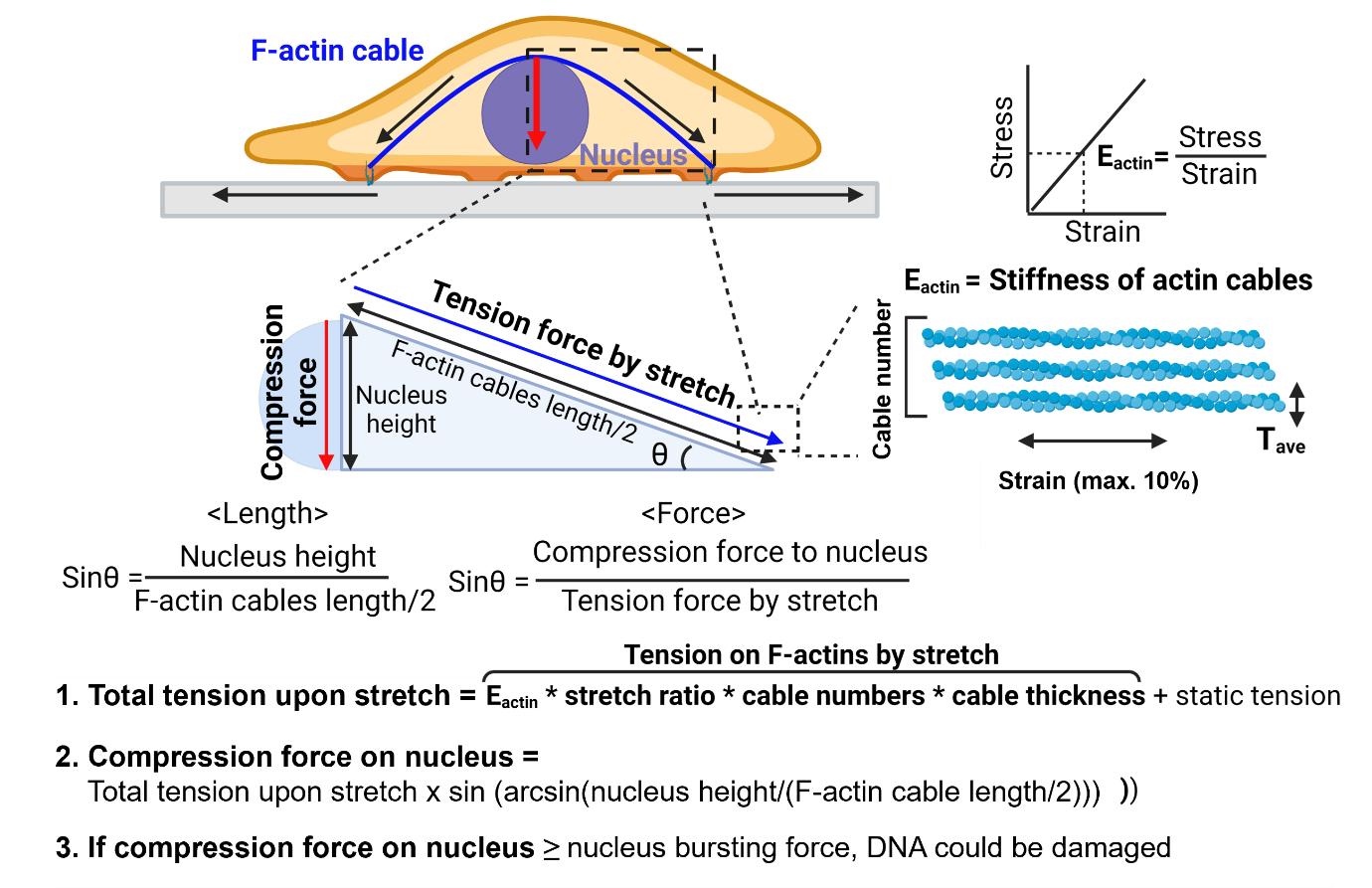


**Figure: Physical model of compression force on the nucleus by F-actin cables driving nucleus bursting.** A computational model assums that the compression force exerted on the nucleus during substrate stretching originates from F-actin cables connecting the top surface of nucleus (actin caps) to the substrate through integrins at both ends. As the substrate stretches up to 10% strain, the F-actin cables elongate (~10%), increasing the tension due to elastic properties. The number and thickness F-of actin cables can further amplify this tension. This tensional force is transmitted to the nucleus, resulting in a compression force that could potentially cause the nucleus to burst and damage the DNA.

To investigate the potential for DNA damage in the cell nucleus under substrate stretch, we developed a computational model to predict the compression force exerted on the nucleus by the stretched F-actin cables (actin caps) above it. The model assumes that elastic F-actin cables form a cap above the nucleus and are anchored to the cell membrane and substrate through integrins at both ends. As the substrate stretches up to 10%, the F-actin cables elongate (~10%), increasing the tension force due to their elastic properties. This tensional force is transmitted to the nucleus, resulting in a compression force that could potentially cause the nucleus to burst and damage the DNA.

The total tensional force (F tension) exerted by the stretched F-actin cap is calculated as the sum of two components: (1) the tension force generated by the stretch of the F-actin cables (F_stretch), and (2) the static tensional force (F_static) present in the cables prior to stretching. The tensional force due to stretching is proportional to the Young’s modulus of F-actin (E_actin_), the strain (λ), the number of cables (N_cable), and the averaged cross-sectional area of the each F-actin cable (AveA_cable). This relationship is expressed as:

F_stretch (N)= E_actin (N/m^2^) × λ x N_cable × AveA_cable (m^2^) (1)

N_cable = initial_cable_number * Δ cable_number (2)

AveA_cable (m^2^) = initial_cable_area (m^2^) * Δ cable_thickness (3)

where E_actin is 10^-9^ Pa, initial_cable_number is 10, initial_cable_area is calculated from π * (initial_cable_diameter/2)^2, and initial cable diameter is 0.2 𝜇m. Ratio of cable number and cable thickness (Δ cable_number and Δ cable_thickness) compared to initial cable number and diameter was given 1~2.5 respectively. 1 is before stretch and ~2.5 reflected enhanced F-actin cables parameters observed in this study.

The total tensional force on F-actin cables is then calculated by adding the static tensional force (F_static) before stretching where F_static (N) = original_tension (N/m^2^) * initial_cable_area (m^2^) * initial_cable_number. The compression force (F_compression) exerted on the nucleus is determined by the total tensional force and the angle (θ) between the F-actin cables and the substrate surface. This angle is calculated using the arcsine of the ratio of the nuclear height (h_nucleus_stretch) under stretch to half the length of the F-actin cables (L_cable/2).

F_tension = F_stretch + F_static (4)

F_compression = F_tension × sin(arcsin(h_nucleus_stretch/(L_cable/2))) (5)

For calculating the nucleus height under stretch (h_nucleus_stretch), the stretched cable length is calculated by initial_cable_length * (1 + stretch_ratio). Initial_cable_length was the average of F-actin cables length. Finally, nucleus_height_stretched is calculated by initial_nucleus_height * (1 - nucleus_height_reduction * stretch_ratio / 0.1) where nucleus_height_reduction is ratio of height reduction under stretch based on the assumption that nucleus_height_stretched is proportionally changed by stretch.

If the compression force exceeds the critical force required for nuclear bursting (F_burst), set at 9 μN based on experimental data, the nucleus is predicted to burst, potentially leading to DNA damage.


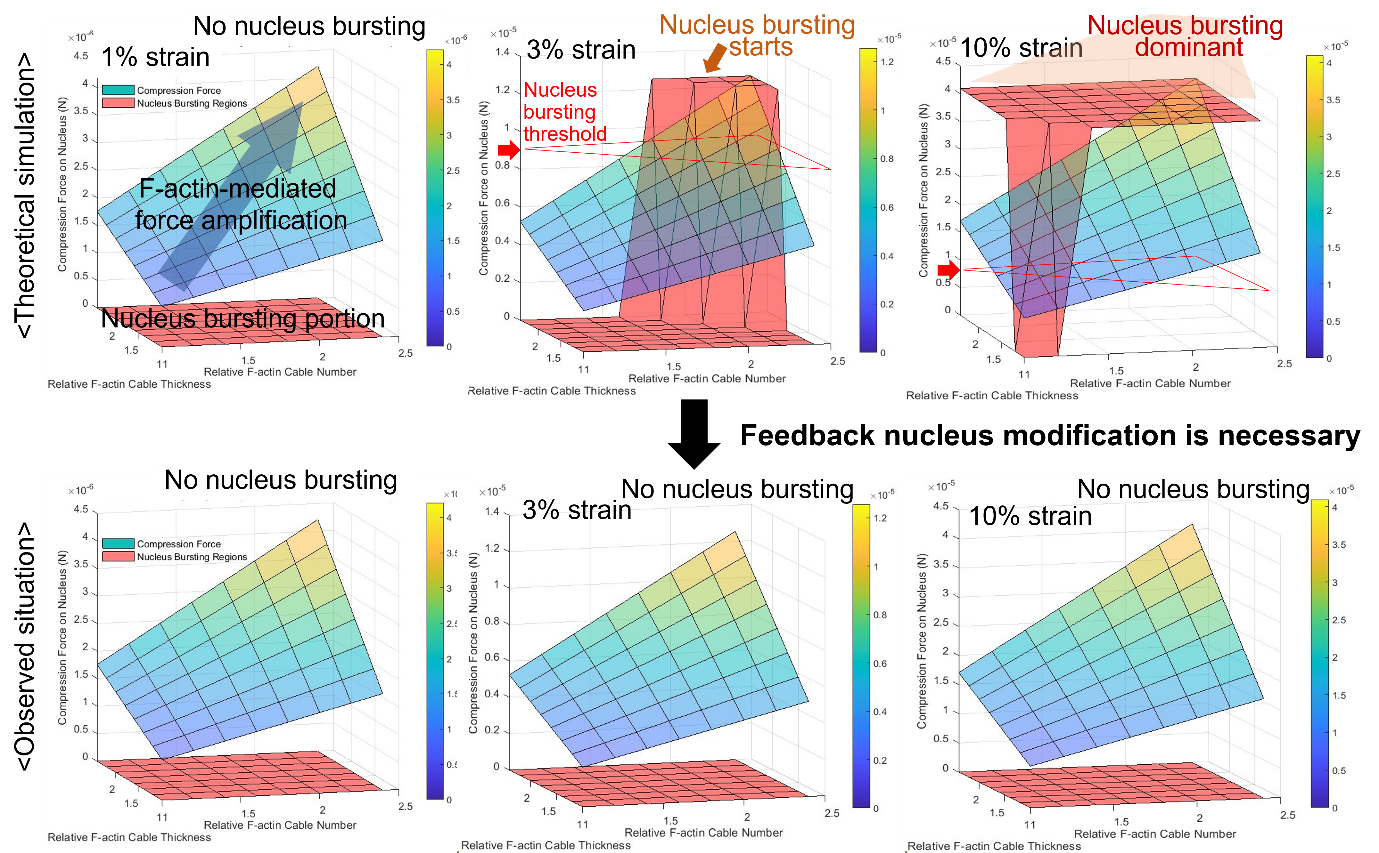


**Figure: Theoretically simulated nucleus bursting under stretch.** Depending on the strain and responsive F-actin cable number (Δ cable_number, x-axis) and their total thickness (Δ cable_thickness, y-axis), compression force (z-axis) on the nucleus could reach the nucleus bursting force (0.9 x 10^-5^ , red arrow in z-axis). The modeling results, visualized with3D surface plots, provide insights into the F-actin cable parameters most likely to amplify stretch-induced compression force, possibly causing DNA damage without feedback modification. The discrepancy between the upper panel (theoretical simulation without feedback nucleus modification) and the lower panel (observed situation) demonstrates the necessity of feedback in nucleus modification to explain the absence of DNA damage under stretch in experiments.

The model was implemented in MATLAB, and simulations were performed for a range of strain ratios (1% to 10%), relative F-actin cable numbers (1 to 2.5), and relative F-actin cable thicknesses (1 to 2.5). The initial_nuclear_height was set to 3.5 μm, and a 3% reduction in nuclear height was assumed under 10% stretch. The F-actin initial cable length was set to 30 μm, and the Young's modulus of F-actin was set to 1 GPa. The initial number of F-actin cables was set to 10, and the static tensional force was set to 100 Pa.

The simulation results were visualized with 3D surface plots, which display the compression force on the nucleus as a function of the relative F-actin cable number and thickness for each strain. Regions where the compression force exceeds the critical bursting force are highlighted in red, indicating the potential for DNA damage. These plots provide insight into the combinations of strain, F-actin cable number, and cable thickness that are most likely to cause nuclear bursting and DNA damage.

In summary, our computational model demonstrates the potential for DNA damage in cells under stretch due to the compression force exerted on the nucleus by the stretched F-actin cap. The model provides a valuable tool for predicting the conditions under which nuclear bursting and DNA damage are most likely to occur, and may inform future experimental studies aimed at understanding the mechanobiology of cell injury and repair.

All model parameters are summarized in the Table, as shown below:

**Table.** Model parameters used in the Supplementary Method S1.

| **Parameter** | **Description** | **value** | **ref** |
| --- | --- | --- | --- |
| F_stretch | Tension force generated by the stretch of the F-actin cables (N) | calculated | - |
| E_actin | Young's modulus of F-actin (Pa, N/m^2^) | 10^-9^ | PMC45560 |
| λ | strain | 0.01~0.1 | Variables |
| N_cable | the number of F-actin cables | calculated | Exp data |
| Δ cable_number | Ratio of cable number under stretch | 1~2.5 | Variables |
| initial_cable_number | initial F-actin cable numbers | 10 | Exp data |
| AveA_cable | the averaged cross-sectional area of the each F-actin cable (m^2^) | calculated | - |
| Δ cable_thickness | Ratio of cable thickness under stretch | 1~2.5 | Variables |
| initial_cable_area | π * (initial_cable_diameter/2)^2 (m^2^) | calculated | - |
| initial_cable_diameter | initial F-actin cable diameter (m) | 0.2 * 10^-6^ | PMC10046292 |
| F_static | the static tension force before stretch (N) | calculated | - |
| original_tension | cellular contraction without stretch (Pa) | 300 | PMC1301364 |
| F_compression | The compression force exerted on the nucleus (N) | calculated | - |
| h_nucleus_stretch | the nuclear height under stretch (m) | calculated | - |
| θ | the angle between the F-actin cables above nucleus and the substrate surface (radian) | calculated | - |
| initial_cable_length | the average of F-actin cables length (m) | 30 * 10^-6^ | Exp data |
| nucleus_height_reduction | ratio of height reduction under stretch | 0.03 | PMID 33667883 |
| F_burst | nuclear bursting force (N) | 9 * 10-6 | PMID 15958226 |
| initial_nuclear_height | initial nuclea height (m) | 3.5 * 10-6 | Exp data |

**Method S2. Model of compression force dissipation in viscoelastic nucleus with change of chromatin status**

To investigate the role of chromatin modifications in modulating nuclear mechanics and preventing DNA damage under mechanical stress, we developed a computational model that predicts the compression force dissipation in a viscoelastic nucleus with changes in chromatin status. The model assumes that the nucleus behaves as a viscoelastic material, which can be described using the Burgers model^7^. The Burgers model consists of a Maxwell element (a spring and a dashpot in series) and a Kelvin-Voigt element (a spring and a dashpot in parallel) connected in series.

**
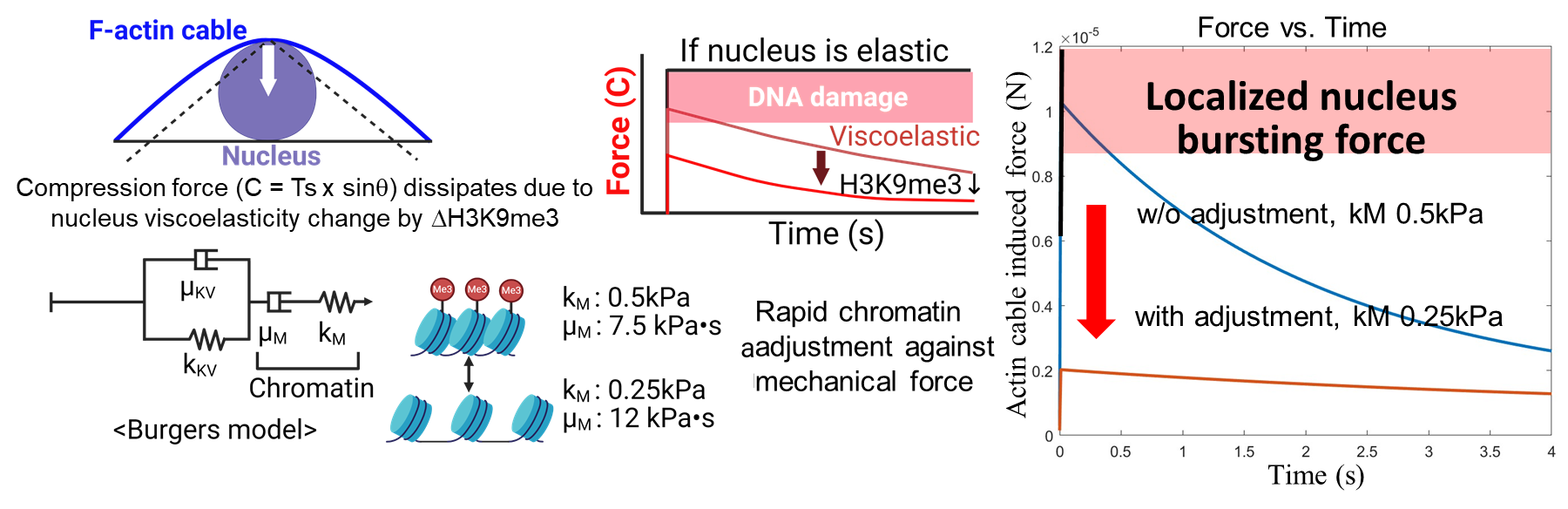
**

**Figure: Model of compression force dissipation in a viscoelastic nucleus with changes in chromatin status.** The strain response of the viscoelastic nucleus under a constant compression force by stretch is calculated based on the 4-element Burgers model.

The strain response of the viscoelastic nucleus under a constant compression force by stretch is calculated based on the Burgers model.

ε(t) = ε0 + ε0 × [(F_compression_N / kM) × (kM / kKV) × (1 - exp(-t/τ)) + (t / μM) × kM] (1)

where ε(t) is the strain at time t, ε0 is the initial strain (0.03), F_compression_N is the compression force under stretch to overall nucleus area, kM and μM are the stiffness and viscosity of the Maxwell element, kKV and μKV are the stiffness and viscosity of the Kelvin-Voigt element, and τ is the relaxation time constant (τ = μKV / kKV). F_compression_N (Pa) is given as compression_force (N) / (Nucleus_area (m)*10000).

Here, the initial strain, representing a 3% reduction in nuclear height under compression at 10% strain by live imaging analysis, was implicated in formular by considering the following assumptions:

1. The nucleus is treated as a homogeneous and isotropic viscoelastic material that can be described using the Burgers model.

2. The compression force applied to the nucleus is assumed to be constant and uniformly distributed across the nuclear cross-sectional area.

3. The initial strain (ε0) represents the instantaneous elastic deformation of the nucleus upon the application of the compression force. This assumption is based on the experimental observation of a 3% reduction in nuclear height under compression at 10% strain by live imaging analysis.

4. The subsequent time-dependent strain response is governed by the viscoelastic properties of the nucleus, which are characterized by the Maxwell and Kelvin-Voigt elements in the Burgers model.

By incorporating the initial strain (ε0) into the Burgers model, we can account for the instantaneous elastic deformation of the nucleus observed in the experimental data. The first term (ε0) represents the initial elastic strain due to the instantaneous deformation of the nucleus upon the application of the compression force. The second term (ε0 × [...]) describes the time-dependent viscoelastic response of the nucleus, which is scaled by the initial strain (ε0). This term consists of two components:

- The first component (ε0 × [(F_compression_N / kM) × (kM / kKV) × (1 - exp(-t/τ))]) represents the delayed elastic strain due to the Kelvin-Voigt element, which exhibits a time-dependent response characterized by the relaxation time constant (τ).

- The second component (ε0 × [(t / μM) × kM]) represents the viscous strain due to the Maxwell element, which exhibits a linear time-dependent response governed by the viscosity (μM) and stiffness (kM) of the Maxwell element.

By combining the initial elastic strain and the time-dependent viscoelastic response, the modified Burgers model provides a more accurate description of the nuclear deformation observed in the experimental data. This modification allows us to capture the instantaneous elastic deformation of the nucleus and the subsequent time-dependent strain response, which is influenced by the viscoelastic properties of the nuclear material.

The force generated by the nucleus in response to the applied strain is calculated as:

F(t) = kM × ε(t) × A_nucleus + kKV × (1 - exp(-t/τ)) × ε(t) × A_nucleus + F_viscous (2)

where F(t) is the force at time t, A_nucleus is the cross-sectional area of the nucleus, and F_viscous is the viscous force, which is calculated as: F_viscous = μM × [0, diff(ε(t)) / dt] × A_nucleus

To model the effect of chromatin modifications on nuclear mechanics, we considered two scenarios: (1) normal cells with a higher initial nuclear stiffness (kM = 0.5 kPa) and (2) cells with a lower initial nuclear stiffness (based on half of nucleus stiffness under stretch, measured by bio AFM, kM = 0.25 kPa) due to the rapid loss of H3K9me3. The other viscoelastic parameters were set as follows: μM = 7500 Pa·s for normal cells and 12000 Pa·s for cells with lower stiffness, kKV = 700 Pa, and μKV = 1500 Pa·s for both scenarios. Initial kM value and others viscoelastic parameters were adopted from^7^. Parameters for normal cells are from WT cells and those from stretched cells are from HDACi-treated cells. All model parameters are summarized in the Table as below:

**Table**. Model parameters used in the Supplementary Method S2.

| **Parameter** | **Description** | **value** | **ref** |
| --- | --- | --- | --- |
| kM_normal | stiffness of the Maxwell element (Pa) | 500 | PMC7175345 |
| μM_normal | viscosity of the Maxwell element (Pa*sec) | 7500 |  |
| kKV_normal | stiffness of the Kelvin-Voigt element (Pa) | 700 |  |
| μKV_normal | viscosity of the Kelvin-Voigt element (Pa*sec) | 1500 |  |
| τ_normal | relaxation time constant (μKV / kKV) (sec) | calculated |  |
| kM_stretch | stiffness of the Maxwell element under stretch (Pa) | 250 |  |
| μM_stretch | viscosity of the Maxwell element under stretch (Pa*sec) | 12000 |  |
| kKV_stretch | stiffness of the Kelvin-Voigt element under stretch (Pa) | 700 |  |
| μKV_stretch | viscosity of the Kelvin-Voigt element under stretch (Pa*sec) | 1500 |  |
| τ_stretch | relaxation time constant under stretch (μKV_stretch/ kKV_stretch) (sec) | calculated |  |
| ε0 | initial nucleus strain reduction at 10% stretch (after 10~30s, ~0.03) | 0.01 | PMID 33667883 |
| initial_cable_diameter | average diameter of actin contacting nucleus (m) | 0.2 x 10-6 | PMC10046292 |
| L_actin_N | length of actin cable contacting nucleus (m) | 7 x 10^-6^ | Exp data |
| F_burst | constant compression force from actin cables (N) | 9 * 10-6 | PMID 15958226 |
| I_actin_N | individual actin cable induced force (N) | calculated | - |

The model was implemented in MATLAB, and simulations were performed for a constant compression force of 9 μN applied to overall nucleus with a cross-sectional area of 150 μm^2^. The initial strain (ε0) was set to 0.03, representing a 3% reduction in nuclear height under compression at 10% strain. The time step (dt) was set to 0.01 s, and the simulations were run for a total of 4 s. 4 s simulation was based on cycle of stretch recipe (4s stretching + 6s rest). DNA damage area from actin cable induced force (N) is based on 9 μN (F_burst) from 60 μm diameter indentor. For calculating individual actin cable induced force (I_actin_N), we assume pressure (Pa) is same between overall and actin cable. Area of actin cable is calculated from ​​the outer surface of a cylinder cut vertically in half contacting top surface nucleus. Diameter of actin and length of actin cable contacting nucleus is given as 0.2 μm (initial_cable_diameter) and 7 μm (L_actin_N) based on reference and nucleus diameter measured in this experiment^8^.

I_actin_N = F_burst / (Area of 60 μm diameter indentor) x (area of ​​the outer surface of a cylinder cut vertically in half (d = 0.2 μm x h=7 μm)) (3)

The simulation results were visualized using plots of force vs. time and force vs. strain for both normal cells and cells with lower initial nuclear stiffness. The plots demonstrate that cells with lower initial nuclear stiffness (representing the loss of H3K9me3) exhibit greater force dissipation compared to normal cells with higher initial nucleus stiffness. This suggests that the loss of H3K9me3 can help prevent DNA damage by allowing the nucleus to deform more easily and dissipate the applied compression force.

References

1 Indana, D. *et al.* Lumen expansion is initially driven by apical actin polymerization followed by osmotic pressure in a human epiblast model. *Cell Stem Cell* **31**, 640-656 e648, doi:10.1016/j.stem.2024.03.016 (2024).

2 Nava, M. M. *et al.* Heterochromatin-Driven Nuclear Softening Protects the Genome against Mechanical Stress-Induced Damage. *Cell* **181**, 800-817 e822, doi:10.1016/j.cell.2020.03.052 (2020).

3 Versaevel, M., Grevesse, T. & Gabriele, S. Spatial coordination between cell and nuclear shape within micropatterned endothelial cells. *Nat Commun* **3**, 671, doi:10.1038/ncomms1668 (2012).

4 Lomakin, A. J. *et al.* The nucleus acts as a ruler tailoring cell responses to spatial constraints. *Science* **370**, doi:10.1126/science.aba2894 (2020).

5 Venturini, V. *et al.* The nucleus measures shape changes for cellular proprioception to control dynamic cell behavior. *Science* **370**, doi:10.1126/science.aba2644 (2020).

6 Kalukula, Y., Stephens, A. D., Lammerding, J. & Gabriele, S. Mechanics and functional consequences of nuclear deformations. *Nat Rev Mol Cell Biol* **23**, 583-602, doi:10.1038/s41580-022-00480-z (2022).

7 Wintner, O. *et al.* A Unified Linear Viscoelastic Model of the Cell Nucleus Defines the Mechanical Contributions of Lamins and Chromatin. *Adv Sci (Weinh)* **7**, 1901222, doi:10.1002/advs.201901222 (2020).

8 Rajan, S., Kudryashov, D. S. & Reisler, E. Actin Bundles Dynamics and Architecture. *Biomolecules* **13**, doi:10.3390/biom13030450 (2023).

**III. Supplementary Table (Table S1~S5)**

**Table S1.** Antibody information

| **Antibody name** | **Company** | **Catalog number** | **Concentration (note)** |
| --- | --- | --- | --- |
| H3K27me3 | Cell signaling | 9733s | 1:800 |
| H3K9me3  H3K9me3 | Novus  Cell signaling | NBP1-30141  13969 | 1:200  1:100 (in vivo) |
| acetyl-Histone H3 | Sigma | 06-599 | 1:100 |
| Emerin | cell signaling | 30853s | 1:400 |
| p-histone H2A.X | Santa Cruz | sc-517348 | 1:50 |
| Phospho-Myosin Light Chain 2 (Ser19)  Phospho-Myosin Light Chain 2 (Ser19) | Cell signaling  Cell signaling | 3675s  3671s | 1:200  1:100 (in vivo) |
| Nesprin 1 | abcam | ab192234 | 1:100 |
| Tubulin β 3  Anti-gamma H2A.X  Anti-Rabbit IgG (H + L) | BioLegend  Abcam  Jackson | 801202  11174  11-095-152 | 1:500  1:100 (in vivo)  1:400 (in vivo) |
| Alpha-SMA | Thermo | 53-9760-82 | 1:200 (in vivo) |
| Syto83 | Thermo | S11364 | 1:1000 (in vivo) |
| CD11b | BioLegend | 101254 | 1:200 (in vivo) |
| H3K9me3 | Abcam | ab288339 | 1:200 (in vivo) |
|  |  |  |  |
|  |  |  |  |

**Table S2.** Primer sequence

| **Gene** | **Forward primer (5'-3')** | **Reverse primer (5'-3')** |
| --- | --- | --- |
| *KDM3A(JHDM2A)* | GCCAACATTGGAGACCACTTCTG | CTCGAACACCTTTGACAGCTCG |
| *KDM3B(JMJD1B)* | GCTCGTAATGTCTGAGAAGGAGG | CACATTTGCGACAAACCCAGTGG |
| *KDM4C(JMJD2C)* | CCGATGACTCTTGTGAAGCAGC | GACTTCGTCTGCCAAAGGTGGA |
| *KDM6B(JMJD3)* | GACCCTCGAAATCCCATCACAG | GTGCGAACTTCCACGGTGTGTT |
| *KDM6A(UTX)* | AGCGCAAAGGAGCCGTGGAAAA | GTCGTTCACCATTAGGACCTGC |
| *KMT1A(SUV39H1)* | CCGCCTACTATGGCAACATCTC | CTTGTGGCAAAGAAAGCGATGCG |
| *KMT1C(G9a)* | GGTGAACAACCACCTGGAGGTA | AGGCTGACCATCTCCAAGTTCC |
| *KMT6(EZH2)* | GACCTCTGTCTTACTTGTGGAGC | CGTCAGATGGTGCCAGCAATAG |
| *KMT1D(GLP)* | GCTTCAGAAGGTGCTCCTCATG | CTGAACCAGCATGTGGCAGATG |
| *KMT8(RIZ1)* | TTGGGCTTGCTCAGGAGAAGAG | GCTGCTATCTCAGGGTTGTCTTC |
| *LMNA* | GACGAGGATGAGGATGGAGA | GAGTGACCGTGACACTGGAG |
| *XPO6* | CACCATAAAACCTTACCTT | AAGGTAAGGTTTTATGGTG |
| *EMD* | CTCTGACTTGAATTCGACT | AGTCGAATTCAAGTCAGAG |
| *SOX2* | TTGCTGCCTCTTTAAGACTAGGA | CTGGGGCTCAAACTTCTCTC |
| *MAP2* | GAAGGGCAACAGAGCTAAA | CCAGACTCAACACCCATAAA |
| *ACTA2* | CTATGCCTCTGGACGCACAACT | CAGATCCAGACGCATGATGGCA |
| *MyoCD* | GCAACACCGATTCAGCTACCTAG | GGTATTGCTCAGTGGCGTTGAAG |
| *RUNX2* | GCTTCATTCGCCTCACAAAC | GTAGTGACCTGCGGAGATTAAC |
| *OCN* | GGCACCCTTCTTTCCTCTT | CACAGATTCCTCTTCTGGAGTT |

**Table S3.** DNA probe sequence

| **Gene** | **Sequence** |
| --- | --- |
| *S100A4* | DNA-[Cy5]CCAGGGCCTCCCAGGCCTTGCCT |
| *FUT4* | DNA-[Texas Red]CGGGGCAGCCGGTGCCCGAAA |

**Table S4.** List of chemicals

| **Name** | **Company** | **Working concentration** | **Function** |
| --- | --- | --- | --- |
| EGTA  (ethylene glycol tetraacetic acid) | Sigma | 2 mM | Inhibition of integrin-ligand binding |
| blebbistatin | Tocris | 40 μM | myosin II inhibitor |
| ML-7 | Tocris | 25 μM | Myosin light chain kinase inhibitor |
| Y-27632 | Tocris | 200 μM | selective ROCK (Rho-associated coiled coil forming protein serine/threonine kinase) inhibitor |
| Cytochalasin D | Tocris | 100 nM | Actin Polymerization Inhibitor |
| CK666 | Sigma | 200 μM | Arp2/3 inhibitor |
| QC6352 | Merck | 2 µM | KDM4 Inhibitor (H3K9me3 enhancer) |
| JIB 04 | Tocris | 400 nM | Histone Demethylase inhibitor (H3K9me3 enhancer) |
| BAPTA-AM | Tocris | 10 µM | intracellular calcium chelator |
| BAPTA | Tocris | 2 mM | extracellular calcium chelator |
| GdCl_3_(Gadolinium(III) chloride) | Tocris | 25 µM | Blocking mechanosensitive ion channels |
| 2APB | Tocris | 100 µM | IP3 receptor inhibitor |

**Table S5.** Summary of analysis timepoints after stretching at different conditions for all experiments

| **Figure** | **Strain (%)** | **Frequency (Hz)** | **Stretch time** | **Rest time** |
| --- | --- | --- | --- | --- |
| Fig. 1b, c | 10% | 0.1 Hz | 0-360 min | - |
| Fig. 1d, e | 10% | 0.1 Hz | 15, 30 min | - |
| Fig. 1f | 5, 10% | 0.1 Hz | 3, 7, 15 min | - |
| Fig. 1g, h | 10% | 0.1 Hz | 3, 15 min | - |
| Fig. 1i – 1j | 10% | 0.1 Hz | 15 min | - |
| Fig. 2 - 4 | 10% | 0.1 Hz | 15 min | - |
| Fig. 5a-5f | 10% | 0.1 Hz | 3, 7, 15 min | - |
| Fig. 5g – 5i | 10% | 0.1 Hz | 15 min | - |
| Fig. 6 - 7 | 10% | 0.1 Hz | 15 min | - |
| Fig. S2 | 10% | 0.1 Hz | 0~360 min | - |
| Fig. S3a | 5, 10% | 0.1 Hz | 3, 7, 15 min | - |
| Fig. S3b | 5, 10, 20% | 0.05, 0.1, 0.2, 0.5 Hz | 15 min | - |
| Fig. S3c | 10% | 0.1 Hz | 3, 15 min | - |
| Fig. S4 – S6 | 10% | 0.1 Hz | 15 min | - |
| Fig. S7 | 10% | 0.1 Hz | 15 min | 15 min, 3 h, 24 h |
| Fig. S8 | 10% | 0.1 Hz | 3, 7, 15 min | - |
| Fig. S9 – S15 | 10% | 0.1 Hz | 15 min | - |
| Fig. S16 | 10% | 0.1 Hz | 15 min per day | 24 h per day |
| Fig. S17a | 10% | 0.1 Hz | 15 min | - |
| Fig. S17b | 10% | 0.1 Hz | 15 min per day | 24 h per day |
| Fig. S18 – S20 | 10% | 0.1 Hz | 15 min | - |
